# Genome-wide association study of childhood B-cell acute lymphoblastic leukemia reveals novel African ancestry-specific susceptibility loci

**DOI:** 10.1038/s41467-025-64337-7

**Published:** 2025-10-22

**Authors:** Cindy Im, Andrew R. Raduski, Lauren J. Mills, Kashi Raj Bhattarai, Robert J. Mobley, Kelly R. Barnett, Zhanni Lu, Kenneth Liao, Nathan Anderson, Rebecca A. Johnson, Erica Langer, Anthony J. Hooten, Alix E. Seif, Kathrin M. Bernt, Matthew Tsang, Brandon A. Mamou, Luis Gil-de-Gómez, Julie A. Wolfson, Danielle N. Friedman, Neerav Shukla, Laura J. Klesse, Erin L. Marcotte, Lingyun Ji, Alice Dang, Minjie Luo, Yiming Zhong, Jalen Langie, Charleston W. K. Chiang, Adam de Smith, Joseph L. Wiemels, Andrew DeWan, Xiaomei Ma, Catherine Metayer, Zhaoming Wang, Heather H. Nelson, Nathan Pankratz, Tianzhong Yang, Saonli Basu, Lucie M. Turcotte, Jun J. Yang, Daniel Savic, Michael E. Scheurer, Logan G. Spector

**Affiliations:** 1 Department of Pediatrics, University of Minnesota, Minneapolis, MN USA; 2 Department of Pharmacy and Pharmaceutical Sciences, St. Jude Children’s Research Hospital, Memphis, TN USA; 3 Division of Oncology, Children’s Hospital of Philadelphia, Philadelphia, PA USA; 4 Department of Molecular Biology, University of Cantabria-IDIVAL, Santander, Cantabria Spain; 5 Institute for Cancer Outcomes and Survivorship, University of Alabama at Birmingham, Birmingham, AL USA; 6 Department of Pediatrics, Memorial Sloan Kettering Cancer Center, New York, NY USA; 7 Harold C. Simmons Comprehensive Cancer Center and the Department of Pediatrics, University of Texas Southwestern Medical School, Dallas, TX USA; 8 Department of Population and Public Health Sciences, Keck School of Medicine, University of Southern California, Los Angeles, CA USA; 9 Children’s Oncology Group, Monrovia, CA USA; 10 Division of Genomic Diagnostics, Children’s Hospital of Philadelphia, Philadelphia, PA USA; 11 Center for Genetic Epidemiology, Department of Population and Public Health Sciences, University of Southern California Keck School of Medicine, Los Angeles, CA USA; 12 Department of Chronic Disease Epidemiology, Yale School of Public Health, New Haven, CT USA; 13 School of Public Health, University of California, Berkeley, CA USA; 14 Department of Epidemiology and Cancer Control, St. Jude Children’s Research Hospital, Memphis, TN USA; 15 Division of Epidemiology and Community Health, University of Minnesota, Minneapolis, MN USA; 16 Department of Laboratory Medicine and Pathology, School of Medicine, University of Minnesota, Minneapolis, MN USA; 17 Division of Biostatistics and Health Data Science, School of Public Health, University of Minnesota, Minneapolis, MN USA; 18 Department of Pediatrics, Section of Hematology-Oncology, Baylor College of Medicine, Houston, TX USA; 19 Texas Children’s Cancer and Hematology Center, Texas Children’s Hospital, Houston, TX USA

**Keywords:** Genome-wide association studies, Cancer genetics, Paediatric cancer, Acute lymphocytic leukaemia, Cancer epidemiology

## Abstract

B-cell acute lymphoblastic leukemia (B-ALL) is the most common pediatric malignancy. Given racial/ethnic differences in incidence and outcomes, B-ALL genome-wide association studies among children of African ancestry are needed. Leveraging multi-institutional datasets with 840 African American children with B-ALL and 3360 controls, nine loci achieved genome-wide significance (*P* < 5 × 10^−8^) after meta-analysis. Two loci were established trans-ancestral susceptibility regions (*IKZF1*, *ARID5B*), while the remaining novel loci were specific to African populations. Five-year overall survival among children carrying novel risk alleles was significantly worse (83% versus 96% in non-carriers, *P* = 4.8 × 10^−3^). Novel risk variants were also associated with subtype-specific disease (*P* < 0.05), including higher susceptibility for a subtype overrepresented in African American children (*TCF3-PBX1*) and lower susceptibility for a subtype with excellent prognosis (*ETV6-RUNX1*). Functional experiments revealed novel B-ALL risk variants had allele-specific differences in transcriptional activity (*P* < 0.05) in B-cell and leukemia cell lines. These findings shed insights into ancestry-related differences in leukemogenesis and prognosis.

## Abstract (web summary)

Disparities in risk and outcomes of pediatric acute lymphoblastic leukemia (ALL) are apparent between different ancestries. Here the authors identify genetic variants with African ancestry-specific risks for developing pediatric B-cell ALL that are also linked to greater 5-year mortality risk.

## Introduction

Although acute lymphoblastic leukemia (ALL) is a rare hematological malignancy, it is the most prevalent cancer type among children^1,2^. ALL of B-cell precursor lineage (B-ALL) is the predominant type, accounting for 85-90% of cases^3^. Given the early age of onset and lack of evidence for strongly associated environmental risk factors^4^, elucidating the basis of inherited genetic susceptibility of childhood B-ALL may be instrumental for identifying children at risk for developing B-ALL or experiencing adverse outcomes. B-ALL includes heterogeneous subtypes typically defined by somatic mutations in leukemic cells identified by cytogenetic or molecular testing, but much remains unclear as to how inherited susceptibility and somatic alterations drive leukemogenesis^5^.

Conducting genomic analyses among individuals of diverse ancestry is critically important to assure the benefits of precision medicine are shared equally by all^6^. Although African American children have a lower incidence of B-ALL compared to other racial/ethnic groups, their outcomes are significantly worse^7–11^. These differences in incidence are not fully explained by perinatal risk factors^12^, and disparities in outcomes are not fully explained by high-risk disease indicators (e.g., unfavorable cytogenetics) or socioeconomic status^11^. Associations between African genetic ancestry and poorer ALL outcomes have been observed^9^, demonstrating the need for genomic analyses of B-ALL among African American children.

Knowledge of how inherited genetic variation contributes to the biology of childhood B-ALL has been informed by previous genome-wide association studies (GWASs), albeit largely in studies among individuals of European ancestry. To date, 24 B-ALL susceptibility loci have been identified^13–24^, including *IKZF1*^13,15^ (7p12.2), *GATA3*^18^ (10p14), *PIP4K2A*^13,20^ (10p12.2), *ARID5B*^21^ (10q21.2), *LHPP*^13,22^ (10q26.13), and *ERG*^13,23^ (21q22.2). Notable recent exceptions include a Japanese GWAS with 1,088 cases^24^ and a trans-ethnic meta-analysis with a discovery dataset with 76,317 participants, including 3482 cases^14^. The latter detected additional trans-ancestral candidate risk loci, including *MYB/HBS1L* (6q23.3), *NRBF2/JMJD1C* (10q21.3), and *TET1* (10q21.3), but primarily included Latino American and non-Latino White cases, while other racial/ethnic groups were poorly represented.

In this study, we show differences in the genetic architecture of childhood B-ALL risk in African ancestral populations with an analysis of 4200 African American children, including 840 B-ALL cases, as a part of the ADMIxture and Risk of Acute Leukemia *(*ADMIRAL) Study. Evidence of further statistical validation in ancestrally diverse B-ALL GWAS datasets was assessed, along with an experimental functional investigation to quantify differences in allele-specific transcriptional activity for novel B-ALL risk variants in relevant cell lines. Associations between novel risk alleles and B-ALL prognosis were evaluated to assess clinical implications.

## Results

### Childhood B-ALL GWAS meta-analysis in African Americans

Two ADMIRAL B-ALL datasets (Supplementary Fig. 1) with participants identifying as African American and with substantial inferred genome-wide (global) African genetic ancestry proportions^25^ (Supplementary Fig. 2) were analyzed. We conducted a discovery GWAS (*n* = 3280 participants, 656 cases), evaluating risk associations for ~11.9 million common genetic variants (sample minor allele frequency [MAF] ≥ 1%), including participants from Children’s Oncology Group (COG) frontline ALL clinical trials as cases and sex-/ancestry-matched controls (Supplementary Table 1). In these data, 46% of cases were female (Supplementary Table 2). A primary replication study was performed utilizing independent B-ALL cases of African ancestry obtained from six institutional biobanks (*n* = 920 participants; 184 cases, 46% female). Summary statistics were combined using a fixed-effects inverse variance-weighted meta-analysis approach^26^.

**Fig. 1:**
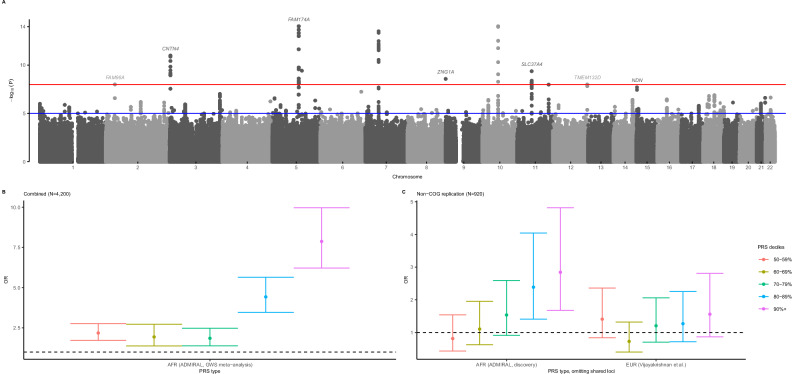
Top B-ALL risk loci in African American children and comparison of genetic ancestry-specific B-ALL polygenic risk scores. Panel **A** shows a Manhattan plot with meta-analysis p-values (y-axis) combining association test statistics from logistic regression models in the discovery and replication samples by variant genomic position (x-axis), with the red horizontal line signifying the genome-wide significance p-value threshold (*P* < 5 × 10^−8^). Novel B-ALL risk loci are annotated with nearest gene names. Odds ratios (ORs, circles) and corresponding 95% confidence intervals (CIs, whiskers) from comparable logistic regression models for increasing deciles of a B-ALL polygenic risk score (PRS) including replicated risk variants achieving GWS after ADMIRAL meta-analysis in African ancestry children are shown in the entire study sample (AFR PRS), with a reference group with median risk or less (panel **B**, *N* = 4200). ORs (circles) with 95% CIs (whiskers) are shown for a PRS including suggestively associated lead variants from the ADMIRAL discovery GWAS (10 variants with *P* < 5 × 10^−6^; AFR PRS) and a PRS based on the largest European B-ALL meta-analysis to date (Vijayakrishnan et al.; EUR PRS) are shown in the replication study sample (panel **C**, *N* = 920) where both PRSs omit overlapping trans-ancestral loci (*ARID5B*, *IKZF1*), also using a reference group with median risk or less. Decile increments of the African ancestry-specific PRS had a dose-response relationship with B-ALL risk in independent ADMIRAL replication data (P_trend_ =1.2 × 10^−6^) while the European counterpart did not (P_trend_= 0.20). All presented p-values are from two-sided statistical tests and are not adjusted for multiple testing.

**Fig. 2:**
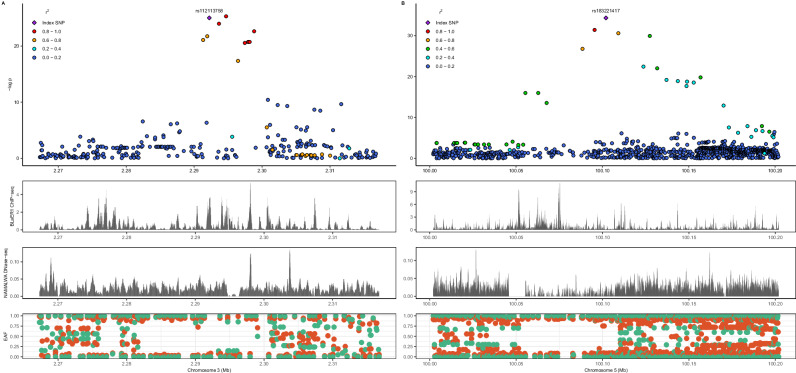
Novel African ancestry-specific childhood B-ALL risk loci at *CNTN4* (left) and *FAM174A* (right). LocusZoom plots of the genomic region surrounding index variants are shown with African ancestry linkage disequilibrium patterns, where variants are color-coded by their magnitude of linkage disequilibrium (LD, r^2^) with the index variant. All presented p-values are from two-sided statistical tests (logistic regression models) and are not adjusted for multiple testing. Window sizes around index variants for *CNTN4* (**A**) and *FAM174A* (**B**) are 50 and 200 kilobases, respectively. Peaks from CEBPA ChIP-seq of B-cell precursor leukemia BLaER1 cell line and DNase-seq of NAMALWA Burkitt’s lymphoma B-lymphocyte cell line are provided for the same genomic window. Effect allele frequencies (EAFs) in African (red) and European (green) 1000 Genomes continental ancestral groups are also provided.

Nine B-ALL risk loci achieved genome-wide significance (*P* < 5 × 10^−8^) after meta-analysis (*n* = 4,200, 840 cases; Table 1) and demonstrated risk associations in both ADMIRAL datasets (i.e., suggestive significance or *P* < 5 × 10^−6^ in the discovery GWAS data and independent nominal replication, *P* < 0.05). Manhattan and quantile-quantile plots are provided (Fig. 1; Supplementary Figs 3, 4), with the latter showing negligible evidence of test statistic inflation (genomic inflation factor [λ]=1.03). Among these, seven are new candidate B-ALL risk loci with large per-allele effect sizes (meta-analysis ORs: 1.87 to 2.94) and specific to African ancestral populations, i.e., rare (<0.01) or absent in other continental 1000 Genomes populations. Two novel risk loci, *CNTN4* (rs112113758, OR = 2.09, *P* = 1.4 x 10^−11^) and *FAM174A* (rs183221417, OR = 2.94, *P* = 1.4x10^−15^), were genome-wide significant in the discovery GWAS. Corresponding genomic regional plots for these novel genome-wide significant B-ALL risk loci are provided in Fig. 2. Regional plots for the other five novel B-ALL risk loci are shown in Supplementary Fig. 5; for the loci showing fewer variants in high linkage disequilibrium (LD) with the index variant, we further evaluated LD patterns in the African 1000 Genomes reference panel and found little evidence of inconsistencies in LD between our data and reference data (Supplementary Fig. 6). Not included in this tally are three independent loci with genome-wide significant variants in the discovery data (rs28568357, 4q35.1; rs115636216, 5q22.1; rs112269413, 11q11) but which did not replicate in ADMIRAL (Supplementary Table 3). No additional conditionally independent B-ALL risk variants were identified in the discovery data.

**Table 1:** Genome-wide significant B-ALL risk loci after meta-analysis identified among African American children^a^

						Meta-analysis (*n* = 4200, 840 cases)		Discovery (COG) *n* = 3280 (656 cases)		Replication (Non-COG) *n* = 920 (184 cases)		1000 Genomes EAFs^b^				
rsid	CHR	BP	EA	NEA	Closest gene	OR (95% CI)	*P*	OR (95% CI)	*P*	OR (95% CI)	*P*	AFR	AMR	EAS	EUR	SAS
Known loci																
rs17133807	7	50409989	A	G	*IKZF1* (+4.9 kb)	1.62 (1.49-1.74)	3.3 x 10^−14^	1.72 (1.50-1.98)	2.1 x 10^−14^	1.27 (0.96-1.67)	0.093	0.16	0.23	0.11	0.32	0.29
rs62445869^c^	7	50409913	A	G	*IKZF1* (+4.9 kb)	1.67 (1.53-1.81)	3.4 × 10^−13^	1.75 (1.50-2.05)	9.5 x 10^−13^	1.37 (1.00-1.87)	0.046	0.09	0.23	0.10	0.32	0.29
rs7090445	10	61961417	C	T	*ARID5B* (0 kb)	1.72 (1.41-2.17)	3.1 x 10^−18^	1.82 (1.45-2.44)	1.1 x 10^−17^	1.37 (1.06-1.79)	0.016	0.19	0.47	0.36	0.33	0.52
Novel loci																
rs77632976	2	34828430	T	C	*FAM98A* (-1229.0 kb)	1.87 (1.66-2.09)	1.0 x 10^−8^	1.87 (1.47-2.39)	5.3 x 10^−7^	1.86 (1.20-2.90)	5.8 x 10^−3^	0.08	*	–	–	–
rs112113758	3	2292232	T	G	*CNTN4* (0 kb)	2.09 (1.88-2.31)	1.4 × 10^−11^	2.20 (1.73-2.81)	1.7 x 10^−10^	1.75 (1.10-2.77)	0.017	0.09	0.01	–	–	–
rs183221417	5	100102043	C	T	*FAM174A* (-433.3 kb)	2.94 (2.68-3.21)	1.2 x 10^−15^	3.04 (2.26-4.08)	1.9 x 10^−13^	2.60 (1.44-4.68)	1.5 x 10^−3^	0.06	*	–	*	–
rs867166159	9	191139	T	G	*ZNG1A* (-12.0 kb)	2.69 (2.36-3.02)	2.7 x 10^−9^	2.51 (1.73-3.65)	1.4 x 10^−6^	3.36 (1.72-6.55)	3.9 x 10^−4^	0.05	*	–	–	–
rs76135126	11	119033074	C	T	*SLC37A4* (-2.2 kb)	2.02 (1.78-2.26)	1.1 × 10^−8^	2.03 (1.55-2.66)	2.5 x 10^−7^	1.99 (1.15-3.44)	0.014	0.08	*	–	–	–
rs113299167	12	129961841	T	C	*TMEM132D* (-57.8 kb)	1.99 (1.76-2.23)	1.1 x 10^−8^	2.00 (1.53-2.62)	5.2 x 10^−7^	1.96 (1.21-3.17)	6.1 x 10^−3^	0.07	*	–	–	–
rs116677565	15	24174608	C	T	*NDN* (-487.3 kb)	2.39 (2.08-2.69)	2.1 x 10^−8^	2.33 (1.63-3.33)	3.0 x 10^−6^	2.54 (1.41-4.58)	1.9 x 10^−3^	0.05	*	–	–	–

*rsid* genetic variant identifier using dbSNP build 151, *CHR* chromosome, *BP* base position, GRCh38 (hg38) build, *EA* effect (risk) allele, *NEA* non-effect (reference) allele, *EAF* effect allele frequency, *COG* Children’s Oncology Group, *OR* odds ratio, *CI* confidence interval, *P* p-value. Odds ratios are from logistic regression models adjusted for the first 3 African ancestry principal components; all presented p-values are two-sided and uncorrected for multiple testing.

^a^Includes loci with genome-wide significant p-values (GWS *P* < 5 x 10^−8^) after meta-analysis showing associations in both datasets (discovery *P* < 5 x 10^−6^ and replication *P* < 0.05).

^b^Asterisks (*) represent EAFs ≥0.001 and <0.01 and dashes (-) represent EAFs <0.001.

^1^Variant within a 1-Mb window closest to index with high LD (R^2^ = 0.71).

Our data showed robust replication of two well-established B-ALL risk loci^13,14,16,22^, *IKZF1* (rs17133807 OR = 1.62, *P* = 3.3 x 10^−14^) and *ARID5B* (rs7090445 OR = 1.72, *P* = 3.1 x 10^−18^). Effect sizes were comparable to previous reports^13,14^ (*IKZF1* ORs=1.43 to 1.65; *ARID5B* ORs=1.64 to 1.80; Supplementary Table 4). Of the 24 evaluable B-ALL risk variants identified from the most recent trans-ancestral meta-analysis^14^ and the largest European ancestry meta-analysis^13^ to date (*n* = 5321 cases), 22 variants had the same directions of association (Supplementary Table 4). A total of 16 variants had lower effect allele frequencies (EAFs) in the 1000 Genomes African versus European populations; of these, 14 variants had cross-population EAF decrements ≥5%. Overall, 12 variants replicated (*P* < 0.05) with similar effect sizes (loci: *IKZF1*, 8q24, *ARID5B*, *GATA3*, *PIP4K2A*, *NRBF2*, *LHPP*, *ERG*). A representative example is *GATA3* variant rs3824662, which is associated with Philadelphia chromosome-like (Ph-like) ALL, a high-risk B-ALL subtype with poor prognosis^18^. Interestingly, rs3824662 shows comparable effect sizes in European ancestry data^13^ (OR = 1.29, *P* = 3.6x10^−14^), trans-ethnic data^14^ (*P* = 1.21, *P* = 1.8 x 10^−9^), and our data (OR = 1.24, *P* = 9.4 x 10^−3^), but considerable cross-population variation in EAFs (8% in African, 19% in European, 27% in East Asian, and 37% in Admixed American 1000 Genomes populations).

### Polygenic risk of B-ALL and ancestry

We evaluated a B-ALL polygenic risk score (PRS) including top index variants after meta-analysis in the combined ADMIRAL study data. African ancestry children in the top PRS-based risk decile had a ~7.9-fold greater odds of B-ALL (95% CI: 6.22-9.97) than those with median risk or less (Fig. 1, Supplementary Table 5). However, this African ancestry-based B-ALL PRS requires validation in external GWAS datasets. To assess cross-population PRS prediction performance, we compared a B-ALL PRS with prioritized index variants from the discovery GWAS (*P* < 5 × 10^−6^) and a B-ALL PRS comprised of genome-wide significant variants identified in the largest European ancestry meta-analysis to date (Vijayakrishnan et al.^13^), each omitting shared loci (*IKZF1, ARID5B*). Decile increments of the African ancestry-specific PRS had a dose-response relationship with B-ALL risk in independent ADMIRAL replication data (Fig. 1, P_trend_ = 1.2 x 10^−6^; top decile vs. ≤median: OR = 2.84, 95% CI: 1.68-4.82); the European counterpart did not (P_trend_=0.20; top decile vs. ≤median: OR = 1.56, 95% CI: 0.86-2.81).

### B-ALL risk alleles and global and local African ancestry

**Fig. 3:**
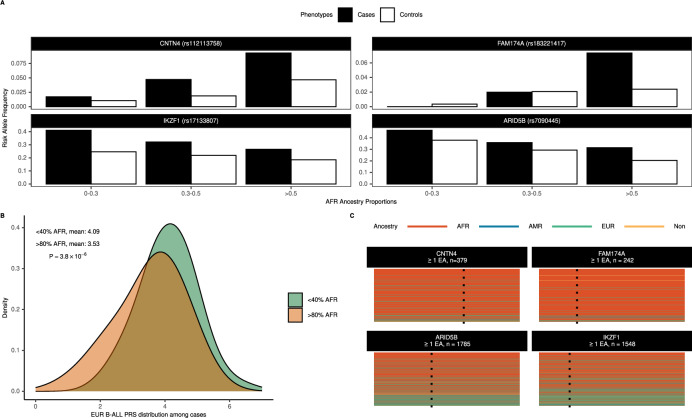
Admixture and relevance of global and local African ancestry on B-ALL risk alleles. Contrasts in risk allele frequencies for index variants in African ancestry cases (filled) and controls (unfilled) stratified by global African ancestry proportions at novel African ancestry-specific loci (*CNTN4, FAM174A*) versus known trans-ancestral loci (*ARID5B, IKZF1*) are shown in panel **A**. Panel **B** shows the distribution of a B-ALL PRS from the largest European meta-analysis to date (Vijayakrishnan et al.) among ADMIRAL B-ALL cases with lower (<40%, green) versus higher (>80%, orange) African global ancestry proportions. The p-value (*P* = 3.8 × 10^−6^) is from a two-sided t-test to assess the difference in means between groups. Differences in the local ancestry tract composition in genomic regions overlapping index variants (dashed line) for African ancestry ADMIRAL participants with at least one effect allele (EA) at novel African ancestry-specific loci (*CNTN4, FAM174A*) versus known trans-ancestral loci (*ARID5B, IKZF1*) are illustrated in panel **C**.

Given that all novel B-ALL risk alleles were largely absent in other continental ancestral groups, we characterized differences in ancestry-specific risk alleles by their associations with individual proportions of global African ancestry. While all top B-ALL risk variants were associated with African global ancestry levels (Supplementary Table 6), increasing doses of previously identified B-ALL risk alleles (*IKZF1* and *ARID5B*) were associated with decreasing proportions of global African ancestry (*P* < 4  ×  10^−6^). In contrast, all novel African ancestry-specific B-ALL risk alleles showed a positive relationship, as expected (Fig. 3, Supplementary Table 6). Aligned with these observations, mean values for the European ancestry-based B-ALL PRS^13^ were higher among cases with lower (<40%) global African ancestry proportions than those with higher (>80%) global African ancestry proportions (Fig. 3; mean_<40%_ = 4.09 versus mean_>80%_ = 3.53, *P* = 3.8  ×  10^−6^).

Next, we investigated the impact of locus-specific or local ancestry, i.e., the number of alleles (zero, one, or two) derived from the African ancestral population. African local ancestry haplotypes overlapping the top B-ALL risk variants were not associated with B-ALL risk (*P* > 0.05, Supplementary Table 7). However, there were differences in local ancestry composition at previously identified versus novel risk loci (Fig. 3) and B-ALL EAFs stratified by African local ancestry background (Table 2). Trans-ancestral B-ALL risk variants (*IKZF1, ARID5B*) had decreasing EAFs in subgroups with increasing numbers of African local ancestry alleles but effect sizes were generally consistent across these subgroups. In comparison, African ancestry-specific B-ALL risk alleles were either rare or absent among individuals without correspondent local ancestry haplotypes. For many of the African ancestry-specific loci, the precision of effect estimates improved in subgroups with increasing African local ancestry haplotypes. For example, the *FAM174A* risk signal was strongly refined among children with homozygous (per-allele OR = 3.36, 95% CI: 2.49-4.53, *P* = 2.1x10^−15^) versus heterozygous (per-allele OR = 1.81, 95% CI: 0.99-3.32, *P* = 0.054) African local ancestry haplotypes.

**Table 2:** B-ALL risk variant associations in African American children, stratified by correspondent local African ancestry haplotypes

	0 local African ancestry haplotypes				1 local African ancestry haplotype				2 local African ancestry haplotypes			
rsid	EAF in cases (n)	EAF in controls (n)	OR	P	EAF in cases (n)	EAF in controls (n)	OR	P	EAF in cases (n)	EAF in controls (n)	OR	P
Known loci												
rs17133807	0.39 (69)	0.28 (275)	1.65 (1.11-2.46)	0.013	0.29 (332)	0.22 (1172)	1.46 (1.2-1.77)	1.4 x 10^−4^	0.25 (439)	0.16 (1913)	1.72 (1.44-2.06)	2.5 x 10^−9^
rs7090445	0.46 (67)	0.38 (258)	1.47 (0.97-2.22)	0.070	0.40 (319)	0.26 (1227)	1.97 (1.62-2.39)	6.9 x 10^−12^	0.26 (454)	0.18 (1875)	1.59 (1.34-1.88)	1.2 x 10^−7^
Novel loci												
rs77632976	0 (71)	0 (311)	–	–	0.06 (327)	0.04 (1060)	1.79 (1.20-2.69)	4.7 x 10^−3^	0.10 (442)	0.05 (1989)	1.96 (1.51-2.53)	3.0 x 10^−7^
rs112113758	0 (62)	<0.01 (368)	–	–	0.06 (322)	0.03 (1042)	1.95 (1.26-3.01)	2.6 × 10^−3^	0.11 (456)	0.06 (1950)	2.13 (1.66-2.73)	2.6 x 10^−9^
rs183221417	0 (86)	0 (337)	–	–	0.03 (283)	0.02 (1102)	1.81 (0.99-3.32)	0.054	0.09 (471)	0.03 (1921)	3.36 (2.49-4.53)	2.1 x 10^−15^
rs867166159	0 (70)	0 (233)	–	–	0.03 (283)	0.01 (1189)	4.28 (1.99-9.17)	1.9 x 10^−4^	0.05 (487)	0.02 (1938)	2.44 (1.7-3.51)	1.5 x 10^−6^
rs76135126	0 (63)	0 (270)	–	–	0.04 (301)	0.03 (1032)	1.43 (0.85-2.40)	0.17	0.09 (476)	0.04 (2058)	2.28 (1.73-3.01)	6.5 x 10^−9^
rs113299167	0 (77)	0 (292)	–	–	0.04 (252)	0.01 (1064)	3.70 (1.97-6.96)	4.7 x 10^−5^	0.09 (511)	0.05 (2004)	1.78 (1.37-2.30)	1.2 x 10^−5^
rs116677565	0.01 (70)	<0.01 (323)	–	–	0.04 (343)	0.01 (1178)	3.18 (1.85-5.46)	2.8 × 10^−5^	0.05 (427)	0.02 (1859)	2.17 (1.49-3.15)	4.9 x 10^−5^

*rsid* genetic variant identifier using dbSNP build 151, *CHR* chromosome, *BP* base position, GRCh38 (hg38) build, *EA* effect (risk) allele, *NEA* non-effect (reference) allel, *EAF* effect allele frequency, *COG* Children’s Oncology Group, *OR*, odds ratio, *CI* confidence interval, *P* p-value. Subgroup odds ratios are from logistic regression models adjusted for the first 3 African ancestry principal components; all presented p-values are two-sided and uncorrected for multiple testing.

### Annotation and validation of ancestry-specific B-ALL risk loci

We broadly annotated the possible molecular consequences of all index variants and their corresponding 99% credible intervals (Supplementary Table 8) at the ten novel candidate B-ALL risk loci (7 replicated loci, plus 3 genome-wide significant loci in discovery data but without replication in ADMIRAL). All 90 credible set variants were well-imputed (Rsq≥0.8). There were no reported hematological trait/disease associations among these variants in the NHGRI-EBI GWAS Catalog^27^. None of the credible set variants at African ancestry-specific B-ALL risk loci were evaluable in the predominantly European gene expression data generated by the GTEx Consortium^28^ (v8, with 85.3% European ancestry donors). However, four index risk alleles were significantly associated with differential whole blood gene expression (*cis-*eQTLs) in admixed populations analyzed by Kachuri et al.^29^: rs28568357 and expression of *CLDN22*; rs115636216 and *EPB41L4A* expression; rs867166159 and *KANK1* expression; rs76135126 and *SLC37A4* and *VPS11* expression. Among these, colocalization testing^30^ showed rs76135126 had some colocalization evidence with expression of divergent transcripts of *VPS11* in whole blood (posterior probability=0.05; Supplementary Table 9). We observed suggestive evidence of colocalization between index variant rs867166159 and expression of an antisense RNA transcript for *DOCK8* (dedicator of cytokinesis 8, *DOCK8-AS1*, posterior probability=0.16), index variant rs113299167 and *ADGRD1* expression (posterior probability=0.12), and index variant rs76135126 and *CD3D* expression (posterior probability=0.12).

Overall, seven of the ten candidate African ancestry-specific B-ALL risk loci contained variants with functional probability scores greater than 0.6 in RegulomeDB^31,32^ (v2), a threshold score that is nominally greater than the mean (0.4) across database variants. Among these, four loci contained variants with significantly greater RegulomeDB functional probability scores in blood and bone marrow cell types (compared to carcinoma-associated variants in the NHGRI/EBI GWAS Catalog^27^; Bonferroni-corrected *P* < 5.6x10^−4^). Six loci had variants that overlapped transcription factor ChIP-seq peaks in leukemia cell lines (BLaER1, K562 [acute myeloid leukemia lineage]) and three loci (*CNTN4*; *SLC37A4*; *CLDN24*) additionally had variants that overlapped DNase-seq peaks in blood cell lines, including in B-cell lymphoblastoid cells (e.g., GM12878) and Burkitt’s lymphoma-derived B lymphocytes (Namalwa). We also identified potential candidate target genes using three-dimensional chromatin interaction (e.g., capture Hi-C, ChIA-PET, HiChIP) data in relevant cell types (Supplementary Tables 10, 11). Chromatin interactions were observed for five out of the ten novel B-ALL risk loci in GM12878, CD34+ hematopoietic progenitor, or ALL (Nalm6) cell lines.

We interrogated novel candidate B-ALL risk variants in multi-ethnic B-ALL datasets from the California Cancer Records Linkage Project (CCRLP). Variants at two loci, *FAM174A* and *NDN*, were replicated (*P* < 0.05) among CCRLP Latino Americans (*n* = 10,450 with 1930 cases), despite having rarer risk allele frequencies (sample EAFs <0.01; Supplementary Table 12). In the smaller CCRLP sample of African Americans (*n* = 2191 with 124 cases), variants at the *NDN* locus were successfully replicated (*P* < 0.05), along with the index variant at the *NREP* locus (rs115636216) which had not replicated in ADMIRAL data (Supplementary Table 13). Geographic differences are an important consideration for interpretation; global African ancestry proportion estimates are lower on average in California African ancestry datasets^33^, e.g., ~74% in the Kaiser Permanente GERA cohort^34^ compared with national African ancestry samples^29^ (83%) and controls in these data (82%).

**Fig. 4:**
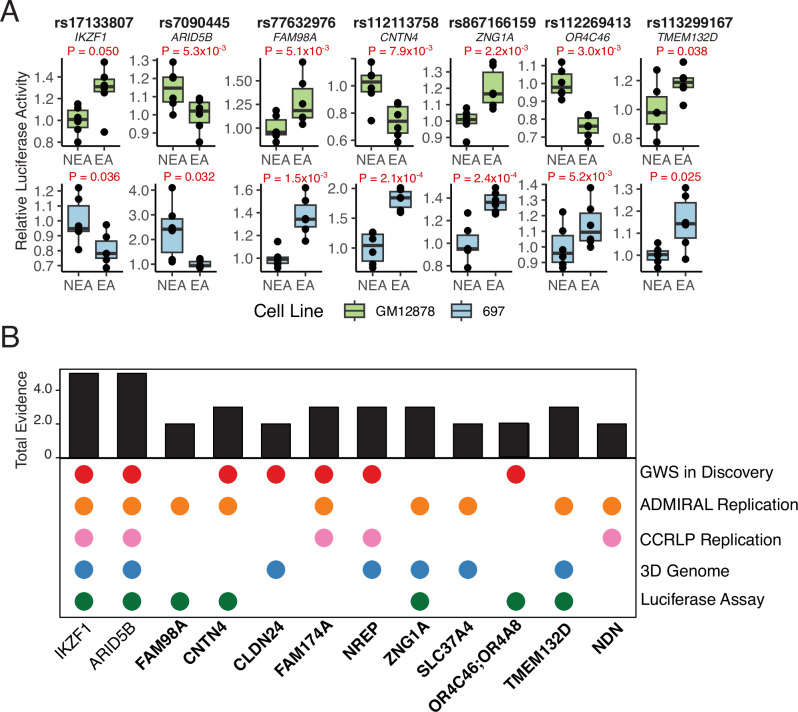
Dual-luciferase reporter assay activity for prioritized B-ALL GWAS variants and overall B-ALL risk loci evidence tally. Panel **A** shows relative dual-luciferase reporter assay activity comparing non-effect (NEA) and effect (EA) alleles at prioritized African ancestry-specific B-ALL GWAS risk variants and known B-ALL risk variants (*IKZF1*, *ARID5B*) with significant allele-specific differences in at least one cell line (ns=not statistically significant). Corresponding relative luciferase expression in B-cell lymphoblastoid (GM12878, green) and leukemia (697, blue) cell lines are shown. Boxplots show median, interquartile range, and minimum and maximum values. In total, 6 biological replicates representing independent transfections were performed for each SNP allele. Biological replicates for each SNP were tested using two independent 96-well plates and the activity for each biological replicate was calculated by taking the average of 3 technical replicates. Statistical significance from two-sided paired t-tests are annotated in each sub-panel. Panel **B** illustrates the overall tally of evidence across study resources for candidate B-ALL risk loci, where novel African-ancestry specific risk loci are bolded.

As an orthogonal source of preliminary evidence for the plausibility of our statistical findings, we performed dual-luciferase reporter assays in B-cell lymphoblastoid (GM12878) and leukemia (697) cell lines for nine out of ten prioritized African ancestry-specific B-ALL GWAS index variants and two known (control) index variants (*IKZF1*, *ARID5B*) with successfully engineered allele-specific plasmid constructs (Supplementary Table 14). Among the 99% credible set variants at each novel locus (Supplementary Table 8), the index variants at seven loci had majority posterior inclusion probabilities (>0.5). In general, these results, along with the lack of additional conditionally independent ALL risk signals, suggested functional experiments focusing on index variants were appropriate. Significant allele-specific changes in transcriptional activity in GM12878 or 697 cell lines were detected for lead variants at known B-ALL risk loci *IKZF1* and *ARID5B* (Fig. 4, Supplementary Table 15). Notably, the effect allele at rs7090445 (*ARID5B*) was associated with decreased transcriptional activity in GM12878 (*P* = 5.3 x 10^−3^) and 697 (*P* = 0.032) cell lines, aligning with previous associations observed between this allele and disrupted MEF2C transcription factor binding affinity and lower *ARID5B* expression^35^. The rs17133807 (*IKZF1*) effect allele was associated with decreased transcriptional activity in the 697 cell line (*P* = 0.036), consistent with the association between this allele and reduced enhancer activity in human pro-B cells^36^. In total, five out of nine candidate African ancestry-specific B-ALL risk variants showed significant allele-specific differences in transcriptional activity. This included variants at *FAM98A* (GM12878: *P* = 5.1 x 10^−3^; 697: *P* = 1.5 × 10^−3^), *CNTN4* (GM12878: *P* = 7.8 x 10^−3^; 697: *P* = 2.0 x 10^−4^), *TMEM132D* (GM12878: *P* = 0.038; 697: *P* = 0.025), and *OR4A8* (GM12878: *P* = 3.0 x 10^−3^; 697: *P* = 5.2 × 10^−3^), as well as index variant rs867166159 (*ZNG1A*) that was tested with neighboring variant rs558007269 as a haplotype (GM12878: 2.2 x 10^−3^, 697: 2.0 x 10^−4^).

Overall, all ten candidate African ancestry-specific B-ALL risk loci had chromatin interaction, secondary statistical, or functional validation evidence (Fig. 4). Four out of five loci achieving genome-wide significance in the discovery GWAS, including two not replicated in ADMIRAL data (*OR4A8*, *NREP*), had secondary statistical or functional validation evidence supporting their contribution to B-ALL risk.

### Greater heritability and familial risk of B-ALL in African Americans

We estimated the single nucleotide variant (SNV)-based heritability using the GREML-LDMS-I method^37^. The heritability on the liability scale was 0.36 (SE = 0.04) among African ancestry children in the ADMIRAL study, appreciably exceeding the most recent comparable estimate of heritability in a Non-Latino White sample with 12,391 participants^14^ (*h*^*2*^ = 0.20, SE = 0.03, with 2,391 childhood B-ALL cases) (Supplementary Table 16). Following published methods^14,38,39^, we found the top nine B-ALL risk variants after meta-analysis, including established B-ALL risk loci at *IKZF1* and *ARID5B*, were estimated to explain 59.2% of the familial relative risk (FRR) in these data (Supplementary Table 17) assuming an ALL familial risk estimate of 3.2 among first-degree relatives^40^. The African ancestry-specific B-ALL risk alleles were estimated to capture half of the FRR (50.4%) in African American children. While the percentage of FRR explained by risk loci identified in the present study exceeds the estimated 23-24% of the FRR explained by risk loci identified to date in Non-Latino Whites and Latino Americans^14^, this estimate should be interpreted with caution given this is based on, to our knowledge, the only available population-based estimates of FRR (from an non-African ancestral population) and may also reflect the effects of “winner’s curse”.

### B-ALL subtypes and overall survival

We assessed associations between index African ancestry-specific B-ALL risk variants and B-ALL biological subtypes in a subset of COG clinical trial participants (Supplementary Tables 18, 19). In these data, the most common subtypes were high hyperdiploidy (34%, *n* = 323, 109 positive), *ETV6-RUNX1* fusion (25%, *n* = 428, 108 positive), and *TCF3-PBX1* fusion (20%, *n* = 285, 57 positive). Specific novel B-ALL risk alleles were nominally associated with B-ALL subtypes, including with increased hyperdiploid (rs77632976, 48% versus 31%, *P* = 0.033), decreased *ETV6-RUNX1* (rs112113758, 13% versus 27%, *P* = 0.020; rs112269413, 9% versus 26%, *P* = 0.034), and increased *TCF3-PBX1* (rs115636216, 37% versus 18%, *P* = 0.039) subtype susceptibility. Carrying novel B-ALL risk alleles was associated with increased *TCF3-PBX1* ALL incidence (23% versus 12%, *P* = 0.036), with rs115636216, rs183221417, rs112269413, and rs76135126 contributing to the ~1.9-fold higher *TCF3-PBX1* incidence in risk allele carriers. A decreased trend with *ETV6-RUNX1* ALL incidence for risk allele carriers was observed, albeit without statistical significance (23% versus 29%, *P* = 0.18).

**Fig. 5:**
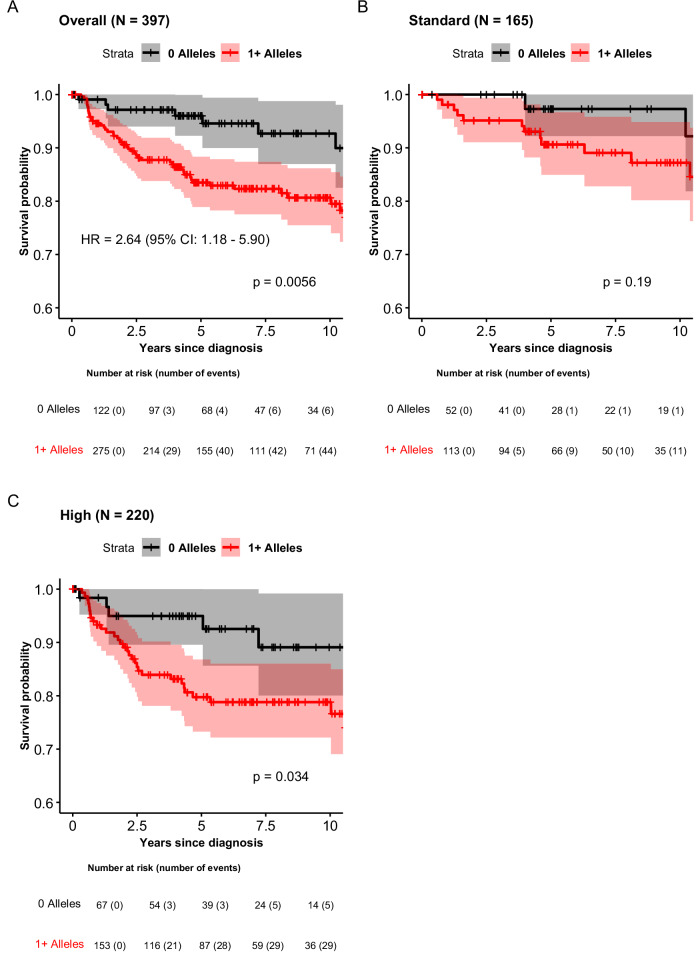
Overall survival after B-ALL diagnosis by novel risk allele carrier status among African American children in Children’s Oncology Group (COG) clinical trials. In all panels, survival curves for participants carrying at least one candidate African ancestry-specific risk allele are shown in red (line) while those who do not are shown in black (line), with censoring status (plus signs) and shaded areas in lighter red and gray representing corresponding 95% confidence intervals (CIs). Panel **A** shows all COG participants in the ADMIRAL study with clinical data, while panels **B** and **C** show subgroups stratified by enrollment in standard versus high risk treatment protocols, respectively. Differences in survival curves were evaluated with two-sided log-rank tests (p-value shown in the lower right quadrant for each panel). In the overall data, the hazard ratio (HR) adjusted for sex, genetic ancestry, and treatment risk stratification is provided along with corresponding 95% CIs.

We evaluated whether carrying candidate African ancestry-specific B-ALL risk alleles was associated with disease prognosis in a subset of COG clinical trial participants with available outcome data (*n* = 397; Supplementary Table 20). In this subset, 54 participants (13.6%) died. The five-year survival probability was significantly worse (*P* = 5.6 x 10^−3^) among individuals carrying ≥1 risk allele (0.83; 95% CI: 0.79-0.88) compared to individuals carrying no risk alleles (0.96; 95% CI: 0.92-1.00) (Fig. 5, Supplementary Table 21). Differences in overall survival by allelic carrier status was seen in subgroups stratified by their enrollment in standard or high risk treatment protocols. Overall, carrying at least one such B-ALL risk allele was associated with an adjusted 2.64-fold greater risk of mortality (95% CI: 1.18-5.90).

## Discussion

Although >90% of childhood ALL cases worldwide occur in low- and middle-income countries in Africa and Asia^41,42^, the majority of genomic studies of ALL that exist to date have been limited to children of European ancestry. Leveraging unique multi-institutional datasets with 840 African American children with B-ALL, this GWAS meta-analysis identified multiple novel pediatric B-ALL risk loci with striking African ancestry-specific risks. All candidate African ancestry-specific B-ALL risk loci were supported by either three-dimensional chromatin interaction data in relevant cell models or secondary statistical or functional validation evidence. These findings underscore the importance of integrative genomic studies of childhood B-ALL susceptibility in underrepresented and admixed ancestral populations.

An important representative finding from our study is the identification of novel African ancestry-specific B-ALL risk locus 3p26.3 (rs112113758). All credible set variants are located in intronic regions of *CNTN4*, a member of the contactin subgroup in the immunoglobulin superfamily whose best characterized associations are for risks for neurodevelopmental and neuropsychiatric disorders^43,44^. We additionally identified a credible set variant at this locus (rs116322842, LD r^2^ > 0.9) with significantly higher RegulomeDB functional probability scores in both blood and bone marrow cell types (*P* < 3 x 10^−9^) and that also overlaps a DNase-seq peak in B-cells and a ChIP-seq peak for a tumor suppressor transcription factor^45^, CEBPA, in BLaER1 leukemia cells. Dual luciferase reporter assays identified significant allele-specific effects in relevant cell types for the index variant. However, we note that additional functional studies are required, including studies considering multiple potentially causal signals at each locus as well as future investigations performed within the endogenous risk locus genomic landscape.

Another representative finding that highlights the strengths of our integrative approach is the identification of novel African-ancestry specific B-ALL risk locus 9p24.3 (rs867166159). We observed three-dimensional chromatin interactions between the genomic region overlapping rs867166159 and the promoter regions of dedicator of cytokinesis 8 (*DOCK8*) in hematopoietic tissue expressing CD34+ cells and B-cell lymphoblastoid cells, as well as suggestive colocalization evidence with *DOCK8-AS1*. Differential expression of *DOCK8* has been observed in specific blood cell types including B-cells, and DOCK8 deficiency has been linked to immunologic phenotypes including T-cell lymphoma-leukemia^46^. We also observed significant increases in relative luciferase activity associated with a haplotype containing the rs867166159 risk allele in B-cell precursor and leukemia cell lines, providing additional validation. While rs867166159 is significantly associated with *KANK1* expression (*P* = 1.8 x 10^−5^) in diverse admixed populations^29^, the contribution of this *cis*-eQTL to B-ALL was not further supported by other bioinformatics analyses. Discussion of the possible molecular consequences of other novel African ancestry-specific B-ALL risk variants are provided in the Supplementary Materials.

Further development of genomic resources among individuals of diverse genetic ancestry is needed (e.g., none of the novel African ancestry-specific B-ALL risk variants were evaluable in GTEx). Colocalization testing with whole blood eQTLs in admixed ancestry populations^29^ highlighted the need for studies of lymphoid precursors or B-cells in African genomes. Despite this limitation, potentially relevant colocalizations for three African ancestry-specific index variants were observed. Aside from the aforementioned *DOCK8-AS1* colocalization, *ADGRD1* encodes a member of the adhesion G protein-coupled receptor (GPCR) family which contributes to immune regulation^47^; more specifically, increased expression of *ADGRD1* has been associated with worse clinical outcomes and poorer survival in acute myeloid leukemia patients^48^. *CD3D* encodes the CD3δ protein; defects in *CD3D* impair T-cell development and is associated with early-onset severe combined immunodeficiency (SCID) with high mortality^49^.

Intriguing features in the known shared genetic risk architecture of childhood B-ALL were observed in the present study. First, consistent with previous analyses^14,24^, these results indicate *IKZF1* and *ARID5B* are trans-ancestral B-ALL risk loci. Second, among the evaluable B-ALL risk variants reported in primarily European populations^13^ or Latino Americans^14^, we found the majority (92%) bore the same direction of risk effects in African Americans. However, two-thirds of these variants had lower EAFs in reference African populations. Third, investigation of a PRS based on genome-wide significant variants from the largest European B-ALL meta-analysis to date^13^ revealed that these scores differed significantly by global African ancestry composition. Overall, it appears that the trans-ethnic polygenic risk for B-ALL may be driven by these ancestry-related risk allele frequency patterns, and are consistent with the historically lower incidence of B-ALL in sub-Saharan Africa and the African diaspora^50^. Further characterization of the trans-ancestral genetic basis of B-ALL risk with larger study samples with greater genomic diversity is needed.

The decreased transferability of a European allele-based B-ALL PRS to African American children is consistent with the broader literature describing the poor cross-population predictive performance of PRSs for other diseases^6,51^. However, this challenge will not be adequately addressed by trans-ancestral meta-analysis approaches alone. Among our major findings, we observed that the ancestry-limited B-ALL risk signals are contingent on finer scale African ancestry, which has implications for future analyses in African-admixed populations. Similar ancestral haplotype-type effects have been reported for alloimmunization response risk after transfusion for sickle cell disease in African ancestry patients^52^. We also observed the liability scale SNV-based heritability estimates of childhood B-ALL risk was appreciably higher in African Americans (*h*^*2*^ = 0.36) in these data compared with non-Latino White participants (*h*^*2*^ = 0.20). These findings can be attributed in part to the higher diversity and smaller LD blocks in African genomes compared to other ancestral populations^53^, but may also indicate GWAS/meta-analyses in African American children focusing on common variants with larger samples sizes will potentially identify additional B-ALL risk loci. On the other hand, we observed that all newly identified African ancestry-specific risk variants had relatively low effect allele frequencies (5-9% in the 1000 Genomes African populations), relatively large effect sizes (per allele ORs of ~2 to 3), and may account for up 50.4% of the familial relative risk of B-ALL. As such, the genetic architecture of B-ALL in African ancestry children may also include additional lower-frequency (or rare) variants yet to be discovered.

Given these African ancestry-specific B-ALL risk variants’ unique characteristics and the well-documented disparities in prognosis by race and ethnicity, we posited that these B-ALL risk alleles may also be associated with worse survival. We found that African American children who carried at least one such risk allele experienced significantly worse survival after B-ALL diagnosis compared to those who did not. Furthermore, we observed interesting patterns of association between the novel B-ALL risk variants and B-ALL subtype-specific susceptibility, particularly *TCF3-PBX1* and *ETV6-RUNX1* fusion ALL. Previous work has reported the higher incidence of *TCF3-PBX1* fusion ALL in African American children^54^ and its association with increasing global African ancestry^9^. The decreased *ETV6-RUNX1* subtype susceptibility associations are noteworthy given its typically excellent prognosis^55^. Along with additional analyses of other prognostic B-ALL indicators, comprehensive subtype-specific genetic association analyses with larger African ancestry sample sizes are planned.

In summary, this study represents, to our knowledge, the first comprehensive genome-wide investigation of childhood B-ALL risk among African Americans. While these results provide further support for the contribution of trans-ancestral loci, marked differences in the genetic architecture of B-ALL risk between African and European ancestral populations were observed, with the identification of multiple African ancestry-specific novel B-ALL loci with relatively large effect sizes. These novel risk variants were associated with specific B-ALL biological subtypes and overall survival. Preliminary functional experiments revealing multiple novel B-ALL risk loci with allele-specific differences in transcriptional activity in relevant cell lines supported the plausibility of statistical findings. More extensive functional validation will be pursued as future work to identify likely leukemogenic mechanisms. Further characterization of childhood B-ALL genetic risk among African Americans is essential to improve B-ALL prediction and may be informative for developing interventions to reduce disparities in prognosis.

## Methods

### Study populations

This study was approved by University of Minnesota institutional review board and by the institutional review boards at each site contributing data (Michigan Department of Health and Human Services, Baylor College of Medicine, University of Alabama at Birmingham, University of Texas Southwestern Medical School, Children’s Hospital of Philadelphia, Memorial Sloan Kettering Cancer Center). All participants or their proxies provided written informed consent for the use of their data in genetic studies of ALL.

#### ADMIxture and Risk of Acute Leukemia (ADMIRAL) Study

Children with a pathologically-verified diagnosis of B-ALL (ICD-O-3 histology 9811-9818, 9820, 9823, 9826, 9827, 9831-9837, 9940, 9948) at 0-25 years of age who identified as Black or African American from the ADMIRAL study were included. Germline DNA samples were obtained from either: (a) COG frontline ALL protocols; (b) the Michigan BioTrust for Health; or (c) multiple institutional academic hospital ALL biobanks (Supplementary Table 1). Remission blood or bone marrow samples and matching clinical information including sex, reported race/ethnicity, and age at diagnosis were obtained from COG protocols 9904, 9905, 9906, AALL0232, AALL1131, AALL15P1, AALL1621, and APEC14B1. Archived newborn dried blood spots (DBS) with matching birth certificate information held by the Michigan BioTrust for Health were obtained for cases and calendar birth year-, sex-, and race/ethnicity-matched (based on mother’s race/ethnicity) controls with no cancer history were obtained as a part of a larger population-based case-control study with linked cancer registry information through the Michigan Cancer Surveillance Program^56^. Buccal cell, saliva, or remission blood or bone marrow samples and matching sex, race/ethnicity, and age at diagnosis information were obtained for cases through ALL biobanks at the Children’s Hospital of Philadelphia (CHOP), Memorial Sloan Kettering Cancer Center (MSKCC), University of Texas Southwestern (UTSW), University of Alabama at Birmingham (UAB), and Baylor College of Medicine. Baylor College of Medicine additionally provided biobanked saliva-based DNA specimens from race/ethnicity-matched controls completing well-child or sports physical visits.

Germline DNA samples from peripheral blood mononuclear cells (PBMC), bone marrow cells, or buccal cells were extracted by the respective treating institutions or at the University of Minnesota by various methods including QIAGEN QIAamp or Puregene (QIAGEN, Germany) or Prepito (Chemagen, Germany). DNA from either frozen PBMC or bone marrow cells was extracted using the QIAGEN Puregene kit per manufacturer’s instructions. DNA from bone marrow slides was extracted using QIAGEN QIAamp kit methods. For newborn DBS, DNA from one 6-mm punch was extracted and purified using the GenTegra GenSolve DNA Complete kit (GenTegra LLC, USA) using standard methods^56^.

#### Additional public controls

Similar to previous GWAS of childhood ALL risk^13,14,16^, we incorporated additional external controls. External controls from a multi-ethnic cohort study of early childhood dental caries with North Carolina children aged 3-5 years who were enrolled between 2016 – 2019 (ZOE 2.0; dbGaP accession: phs002232.v1.p1)^57^ were included. Details related to study design, including specimen collection and genotyping, have been described previously^57^. In brief, data for a total of 6144 external controls genotyped using the same genotyping platform used for ADMIRAL study samples were available (Illumina Global Diversity Array [San Diego, CA]; described below). Any ZOE external control that did not identify as Black or African American, multiple, or other race/ethnicity was excluded from further analyses.

### Genotyping and pre-imputation quality control

All ADMIRAL study samples were genotyped in three rounds at the University of Minnesota using the Illumina Global Diversity Array 8v1 (San Diego, CA), assaying 1,904,599 single nucleotide variants (SNVs). Across rounds, 1,081,047 to 1,292,439 non-monomorphic autosomal variants were directly measured. Hard genotype calls were inferred using Illumina Genome Studio software (v2.0.5) and data were aligned to the GRCh37 human genome assembly. Samples with initial probe hybridization call rates <0.95 were not used for genotype clustering. Sex was inferred using measured array probe intensities on the sex chromosomes within Genome Studio.

The same pre-imputation quality control (QC) procedures were applied in parallel for each dataset, including external controls. PLINK v1.90b6.10^58^ and BCFtools v1.2^59^ were used to perform QC. Samples with >5% missingness, sex discordance, or excess heterozygosity (±3 SD from sample mean) were excluded. Among autosomal variants, criteria for exclusion included: >5% missingness; minor allele frequency (MAF) <1%; and deviation from Hardy-Weinberg equilibrium in controls (*P* < 1 x 10^−7^). Across datasets, 114,380 to 364,748 sites were filtered. A total of 7,838 study participants (1087 cases, 6751 controls) and 724,685 SNPs were present across all datasets after pre-imputation QC.

### Genetic ancestry inference

All participants included in analyses primarily reported Black or African American race/ethnicity. RFMix^25^ (v2.03-r0) was used to infer the number of alleles inherited from African (AFR), Admixed American (AMR), East Asian (EAS), European (EUR), and South Asian (SAS) ancestral populations at each locus. Genotype data that passed pre-imputation QC were pre-phased using SHAPEIT^60^. Default parameters were applied, including a window size of 0.2 cM and an assumption of 8 generations since admixture. The 1000 Genomes populations were used as the reference panel (*n* = 883 AFR, 535 AMR, 621 EAS, 562 EUR, 661 SAS). Phased alleles were assigned to the ancestral population with the maximum estimated posterior probability. Global ancestry proportion estimates were obtained by summing the proportions of local ancestry tracts assigned to each ancestral population. We excluded study participants with global AFR genetic ancestry proportions that were 5 SD below (16.9%) the mean among self-reported Black or African American ZOE controls (81.9%) (*n* = 135 cases). Eigenvector bi-plots from EIGENSTRAT-based principal component analysis^61^ (PCA, performed with PLINK^58^ v1.9) combining study samples meeting the global AFR genetic ancestry threshold with the 1000 Genomes populations were inspected. Evaluation of eigenvector bi-plots considering the first three ancestry principal components indicated all included study samples appeared to cluster consistently with 1000 Genomes AFR reference samples (Supplementary Fig. 2).

### Imputation and case-control matching

All datasets were imputed separately using the NHLBI Trans-Omics for Precision Medicine (TOPMed) multi-ancestry reference panel^62^ (version r2 with 97,256 samples, hg38 build), using Eagle v2.4^63^ for phasing and Minimac4^64^ for imputation. The choice of imputation panel was informed by recent work by Hanks et al.^65^ showing that genotyping with arrays with >700,000 SNVs followed by imputation with the TOPMed imputation reference panel can approximate deep whole-genome sequencing, leading to datasets where ≥90% of bi-allelic SNVs are well imputed (squared Pearson correlation coefficient r^2^ > 0.8 between the imputed genotype dosages and the sequence-based genotypes) down to MAFs of 0.14% in African ancestry populations. Genetic variants with a genotype posterior probability of ≥0.85 were retained, and variants with low imputation quality (r^2^ < 0.5) were removed, along with multi-allelic and insertion/deletion variants. Post-imputation, data were merged and variants with MAF < 1% and deviation from Hardy-Weinberg equilibrium in controls (*P* < 1x10^−7^) were excluded. Duplicates and samples showing evidence of cryptic relatedness (identity-by-descent proportions or IBD pi-hat>0.25) were excluded (*n* = 112 cases), prioritizing cases if included in sample pairs with controls. Consistent with methods to identify variants with potential batch effects^66^, we conducted pseudo-GWAS and conservatively removed variants with *P* < 5x10^−8^, considering all pairwise comparisons among batches within cases and internal versus external sources within controls. PCAMatchR^67^ was used to match up to four controls to each case by sex and finer-scale ancestry. To generate ancestry-informative PCs to include as covariates to control for cryptic population substructure, EIGENSTRAT-based PCA^61^ was performed with PLINK^58^ v1.9 in the final analytic sample.

### Discovery association analysis

We conducted a discovery GWAS in data where cases were from COG B-ALL frontline trials (*n* = 3280, with 656 cases), considering 11,876,503 autosomal SNVs available for analyses. Logistic regression models adjusted for the first three AFR ancestry PCs evaluated pediatric B-ALL risk associations with common genetic variants, assuming an additive genetic effect model. Variants with association test p-values < 5 x 10^−8^ were considered to be genome-wide significant. However, to facilitate the discovery of novel B-ALL risk loci in this unique study of African American children, we prioritized all variants with discovery association test p-values < 5 x 10^−6^. Independent loci were defined via the PLINK^58^ v1.9 LD clumping algorithm (--clump), where mutually exclusive LD-based clumps of variants within 500 kb of the index variant (defined by the smallest p-value in discovery) with an LD r^2^ > 0.01 were formed. Stepwise conditional analyses were conducted to identify additional independent associations, where association tests for variants at each prioritized locus were conditioned on the most significantly associated variant at that locus. A conditional association test p-value threshold of <5x10^−6^ was considered to prioritize secondary independent signals.

### Primary replication and meta-analysis

All variants at loci prioritized in the discovery GWAS were tested for evidence of replication (*P* < 0.05) in data where cases originated from non-COG study sources (*n* = 920, with 184 cases). Association statistics from discovery and replication analyses were meta-analyzed using the fixed-effects inverse variance-weighted method implemented in METAL^26^ for all variants at prioritized loci. Heterogeneity was examined using Cochran’s Q test^68^ and I^2^ index^69^. Meta-analysis for variants with evidence of allelic heterogeneity (P_het_<0.05) was performed using the Han-Eskin random-effects model (RE2) in METASOFT^70^.

Variants achieving genome-wide significance after meta-analysis (*P* < 5 x 10^−8^) that: (a) showed at least suggestive association in discovery data (*P* < 5 x 10^−6^); (b) had evidence of replication (*P* < 0.05); and (c) were >1 Mb from previously reported pediatric B-ALL risk variants (compiled by Jeon et al.^14^) were considered to be novel pediatric B-ALL susceptibility loci.

### Polygenic risk score (PRS)

For each study participant, we computed a childhood B-ALL PRS consisting of 17 genome-wide significant variants associated with B-ALL or common subtypes of B-ALL (*ETV6-RUNX1* fusion positive ALL and high-hyperdiploid ALL) from a previous meta-analysis conducted by Vijayakrishnan et al.^13^ in predominantly European ancestral samples (Supplementary Table 4). We implemented recommended best practices for PRS computation^71^ (PLINK v1.9^58^, --score), including common biallelic risk variants passing aforementioned QC procedures, and applied original publication weights (log[OR]) to support comparability across studies. We also computed a childhood B-ALL PRS including index variants achieving genome-wide significance after meta-analysis in ADMIRAL data (using meta-analyzed summary statistics, Table 1). To appropriately compare differences in risk associations between B-ALL PRSs derived from different ancestral samples, we assessed the Vijayakrishnan et al.^13^ European ancestry B-ALL PRS and the African ancestry B-ALL PRS developed with our discovery GWAS results (including 10 index variants at prioritized loci [*P* < 5x10^−6^] using log[ORs] from discovery, Supplementary Table 3), each omitting shared loci (variants at *IKZF1* and *ARID5B*), exclusively in the replication data. Associations between B-ALL and standard deviation increases in scaled PRS values or PRS deciles (reference: ≤median) were evaluated using the aforementioned statistical models.

### B-ALL variant and PRS risk associations considering global and local ancestry

Within an African American admixed population, B-ALL risk variant associations may vary with differing levels of global AFR ancestry. Furthermore, given the identification of multiple ancestry-specific childhood B-ALL risk variants, it is unclear whether local ancestry (i.e., carrying zero, one, or two AFR ancestral alleles in a genomic region) may be a better-powered proxy for identified risk variants or is otherwise informative for risk variant associations. Therefore, we assessed top B-ALL risk variant associations with global AFR ancestry, separately among B-ALL cases and controls, using linear regression models and a Bonferroni-corrected p-value threshold (*P* = 5.6x10^−3^ or 0.05/9 variants) to identify statistically significant associations. We also evaluated local ancestry allelic associations with B-ALL for genomic regions overlapping the top identified risk variants using logistic regression models, adjusting for global AFR ancestry and the first AFR ancestry PCs, with the same Bonferroni-corrected p-value threshold (*P* = 5.6 x 10^−3^ or 0.05/9 variants). Risk variant associations with B-ALL were further assessed among subgroups with zero, one, or two local ancestry alleles (correspondent with the genomic regions overlapping top risk variants), using the same logistic regression models applied in primary analyses. Risk associations between different ancestry-specific PRS and B-ALL were also assessed in subgroups stratified by varying levels of AFR global ancestry.

### Functional annotation of genetic credible sets and colocalization

Consistent with research practices described in previously published ALL GWAS meta-analyses^13,14,16,22^, we conducted preliminary fine mapping and broadly annotated the potential molecular consequences of putative risk variants, with the goal of providing comprehensive evidence identifying the most reasonable gene targets for each genetic variant of interest. We constructed 99% credible intervals, i.e., sets of variants at each locus with a 99% posterior probability of containing the causal variant, for each distinct B-ALL risk signal using a Bayesian approach^72^ to define genomic regions with 99% credible interval coverage and permit broader functional annotation of novel B-ALL risk loci. With meta-analysis summary statistics, we calculated approximate Bayes factors given by ${{BF}}_{k}=\sqrt{1-{R}_{k}}\exp \left(\frac{{R}_{k}{\beta }_{k}^{2}}{2{\sigma }_{k}^{2}}\right)$ where *β*_*k*_ and *σ*_*k*_ are the per allele log(OR) and standard error of variant *k*, respectively, and ${R}_{k}=0.04/({\sigma }_{k}^{2}+0.04)$ using a Gaussian prior *N*(0,2^2^)^72^. We then computed the posterior probability that the *k*^*th*^ variant is causal given by ${\pi }_{k}={{BF}}_{k}/{\sum }_{1}^{K}{{BF}}_{k}$. The 99% credible set was then constructed by ordering variants by their posterior probabilities from highest to lowest and including variants until the cumulative posterior probability was 0.99.

All variants included in 99% credible intervals at novel B-ALL risk loci were mapped to their nearest gene using ANNOVAR^73^. Detailed functional/regulatory consequences, including information related to ENCODE^74,75^ transcription factor binding sites and motifs, chromatin accessibility, and DNase hypersensitivity (e.g., transcription factor ChIP-seq, DNase-seq/ATAC-seq), were evaluated using information from RegulomeDB^31,32^ (v2.2). Specifically, for all credible set variants, we obtained: (a) RegulomeDB heuristic ranking scores; (b) RegulomeDB cell type-agnostic probabilistic scores for each variant’s potential to be functional in regulatory elements (ranging from 0 to 1); and (c) RegulomeDB blood- and bone marrow-specific functional probability scores (ranging from 0 to 1). Z-scores based on the distribution of cell type-agnostic functional probability scores for every variant in RegulomeDB were used to compute one-sided p-values to assess whether a given B-ALL risk variant’s functional probability score was greater than the mean. Considering the 4,586 SNPs associated with any carcinoma (i.e., non-hematological cancer) reported in the NHGRI-EBI GWAS Catalog^27^ as a comparison group, one-sided p-values indicating how extreme (i.e., greater) the blood and bone marrow functional probabilities are for each credible set variant were calculated based on Z-scores from the null distributions of corresponding blood and bone marrow RegulomeDB functional probability scores for the comparison SNPs (Bonferroni-corrected *P* < 0.05/90 = 5.6x10^−4^). We provided detailed RegulomeDB annotations in relevant cell types (e.g., hematopoietic multipotent progenitor cells, CD34+ hematopoietic progenitor cells, any primary B-cell, lymphoblastoid cell lines from 1000 Genomes [i.e., GM19238], K562, NAMALWA, BLaER1) for: (a) all index variants; and (b) credible set variants with nominally significant (*P* < 0.05) functional probability scores. All credible set variants were assessed for previously reported disease/trait associations in the NHGRI-EBI GWAS Catalog^27^. We also sought to identify significant allelic associations with gene expression and changes in chromatin structure (e.g., expression or *cis*-eQTLs and chromatin accessibility or caQTLs, FDR < 0.05) among all credible set variants in GTEx^76^. All credible set variants were also queried for significant associations with whole blood gene expression (*cis*-eQTLs; FDR < 0.05) in diverse ancestry samples previously analyzed by Kachuri et al.^29^ Putative chromatin state annotations for regulatory states including promoters (states 1,2) and enhancers (states 6, 7) based on the 15-state ChromHMM model trained on 12 epigenetic marks for 127 epigenomes^77^ from the Roadmap Epigenomics Consortium were obtained using bedtools.

To evaluate the effects of credible set variants on gene expression, we tested for colocalization between our B-ALL GWAS meta-analysis associations and the whole blood *cis*-eQTLs reported by Kachuri et al.^29^ in admixed ancestry populations using a Bayesian approach implemented through the coloc.abf function with default priors in the coloc R package^30^ (v5.2.3). We considered all variants within a 1-Mb region flanking the index variant and any gene that was a significant eQTL (FDR < 0.05) within the same window. Due to the lack of more directly relevant molecular/genomic datasets (e.g., lymphoid precursors or B-cells) among individuals of African ancestry, we considered colocalization posterior probabilities >0.1 as evidence of suggestive colocalization between credible set variants and whole blood gene expression.

### SNP-gene associations using chromatin looping data

SNPs were mapped to candidate target genes using promoter capture Hi-C, HiChIP (Hi-C with chromatin immunoprecipitation-sequencing) and CHIA-PET (chromatin interaction analysis by paired-end tag sequencing) data from primary cells (primary CD34+ hematopoietic progenitor cells [*n* = 1]^78^, primary total B-cells [*n* = 1]^79^, primary naïve B-cells [*n* = 1]^79^ and primary B-ALL cells [*n* = 10]^80^) and cell lines (GM12878 B-lymphocyte cells^74,75,78^ and 697^81,82^, BALL1^81^, Nalm6^81,82^, REH^81^, RS411^81^, SEM^81^ and SUPB15^81^ B-ALL cell lines; see Supplementary Table 10). Paired-end BED files were obtained from these datasets for intersections with SNP coordinates using bedtools (“*pairToBed -type either”* command) or chromatin looping data was manually visualized using the Yue lab Computational and Functional Genomics/Epigenomics website browser (http://3dgenome.fsm.northwestern.edu/index.html). Chromatin looping events were identified for both promoter-proximal and promoter-distal SNPs. Candidate target genes of promoter-distal SNPs were identified by three-dimensional chromatin interactions to gene promoters or gene bodies. Promoter-proximal SNPs were assigned to the proximal gene.

### Secondary validation in the California Cancer Records Linkage Project (CCRLP)

Data from a recent trans-ethnic meta-analysis^14^ were used for secondary validation/replication of candidate B-ALL risk variants. Details about study design and methods have been previously described^14,16^. In brief, given that most candidate variants were rare but not absent in Admixed American populations in the 1000 Genomes reference panel, we assessed CCRLP GWAS data in Latino Americans imputed with the TOPMed multi-ancestry reference panel^62^ including 1,930 cases and 8,520 controls (from CCRLP and the Genetic Epidemiology Research on Aging Cohort [GERA]) to evaluate 99% credible interval variants at each B-ALL risk locus. Logistic regression models tested variants meeting the imputation quality threshold (r^2^ ≥ 0.3) with MAFs >0.001 (minor allele count >50), adjusting for top 20 ancestry PCs. We also examined credible set variants in the CCRLP GWAS data in African Americans imputed with the Haplotype Reference Consortium (r1.1) reference panel, including 124 cases and 2,067 controls (from CCRLP and GERA). Some CCRLP cases in African Americans and cases in the ADMIRAL discovery data may potentially overlap; however, the impact is likely negligible given the vast majority of ADMIRAL discovery cases have birthplaces outside of California. Logistic regression models adjusting for top 20 ancestry PCs were used to test variant risk associations, considering variants meeting the imputation quality threshold (r^2^ ≥ 0.3) with MAFs >1%.

### Luciferase reporter assays

Dual-luciferase reporter assays remain well-established as a preliminary approach to assess the plausibility of GWAS meta-analysis findings^83–86^ and were used to test DNA sequence oligonucleotides centered on non-effect or effect alleles at each SNP in the same sequence orientation (oligonucleotide length range = 301-1001 bp; see Supplementary Table 14 for sequences). Because oligonucleotides could not be engineered for rs112269413 and rs876166159, a two-stage PCR was employed to amplify DNA centered on each SNP variant using genomic DNA from the Coriell Institute for Medical Research (#NA19704) heterozygous for each SNP (see Supplementary Table 14 for sequences and PCR primers). For rs867166159, two haplotypes containing alleles for rs558007269 and rs867166159 (reference haplotype= G,G; alternative haplotype= A,T) were tested. DNA oligonucleotides or amplified PCR products were cloned into the pGL4.23 plasmid vector (Promega, #E841A) upstream of the minimal promoter using an EcoRV restriction enzyme site. Following molecular cloning and verification using Sanger DNA sequencing (see Supplementary Table 14 for pGL4.23 plasmid backbone primer), 2.5x10^5^ cells per replicate GM12878 B-lymphoblastoid cells (Coriell Institute for Medical Research, #NA12878) or 697 B-cell precursor acute lymphoblastic leukemia cells (DSMG; #ACC 42) were co-transfected with oligonucleotide-containing pGL4.23 firefly luciferase and Renilla plasmid reporter gene constructs using the Neon transfection system (10 μL Neon tips, Thermo Fisher Scientific, MPK5000, 1 μg plasmid DNA and 0.1 μg pRL-TK control vector) and the following transfection parameters: GM12878 = 1200 V, 20 ms, 3p; 697 = 1600 V, 10 ms, 3p. Following a 24-hour incubation, firefly luciferase and Renilla activity was quantified using the Dual Luciferase Reporter Assay System (Promega, E1960) on a BioTek Cytation1 plate reader (Agilent). Luciferase activity was calculated as the ratio of firefly luciferase to Renilla luciferase activity. Relative luciferase activity was determined by normalizing to reference allele activity. In total, 6 biological replicates representing independent transfections were performed for each SNP allele. Biological replicates for each SNP were tested using two independent 96-well plates and the activity for each biological replicate was calculated by taking the average of 3 technical replicates. Statistical significance was calculated using two-sided paired Student’s t-tests. Given that both consistent and opposing luciferase activity between the two cell lines may plausibly reflect true cell-specific differences in regulatory elements that affect functional activity^81,87–90^, we considered consistent and opposing effects across the cell lines as evidence of functional validation (*P* < 0.05).

### Heritability

We estimated the multicomponent narrow-sense SNV-based heritability for B-ALL in African Americans using the combined imputed ADMIRAL data (MAF ≥ 0.05, *n* = 4200) with the individual LDMS GREML (GREML-LDMS-I) method^37^, as implemented in Genome-wide Complex Trait Analysis (GCTA) software^91^ (v1.94.1). GREML-LDMS-I has been shown to generate more accurate heritability estimates compared to other methods even when MAF-/LD-related assumptions about the genetic architecture are misspecified^37^. Regional LD scores (200 kb segments) were computed and SNVs were assigned to bins defined by LD score quartiles further stratified into 4 MAF categories, yielding 16 genetic relatedness matrices (GRMs). Restricted maximum likelihood (REML) estimates of heritability were obtained using the first 3 ancestry PCs as fixed effects and multiple GRMs as random effects in a mixed effect model and were converted to the liability scale using the population prevalence of ALL among African Americans (1.47x10^−4^) based on data from the US National Cancer Institute’s Surveillance, Epidemiology, and End Research Program (SEER; https://seer.cancer.gov/seerstat).

### Percentage of familial relative risk explained

The percentage of familial relative risk (FRR) of ALL explained by each of the top risk variants from this analysis was estimated using previously adopted methods^14,38,39^. In brief, the FRR due to locus *k* is estimated by ${\lambda }_{k}=\frac{{p}_{k}{r}_{k}^{2}+{q}_{k}}{{\left({p}_{k}{r}_{k}+{q}_{k}\right)}^{2}}$, where *p*_*k*_ is the ancestral risk allele frequency for locus *k*, ${q}_{k}={1-p}_{k}$, and *r*_*k*_ is the per-allele OR from meta-analysis. The contribution of the top B-ALL risk variants to the familial risk is $\frac{\mathop{\sum }_{k}\log {\lambda }_{k}}{{\lambda }_{0}}$, where *λ*_0_ is the observed familial relative risk among first-degree relatives of ALL cases (estimated to be 3.2 using Swedish/Finnish national registry data^40^).

### B-ALL subtype categorization and overall survival

To evaluate associations between African ancestry specific B-ALL risk variants and B-ALL biological subtypes, we evaluated a subset of COG clinical trial participants with subtype information based on fusion gene, expression profile, point mutation, karyotype data or fluorescence in situ hybridization (FISH) data. For participants who also had ISCN codes, the cytogenetic nomenclature from karyotype or FISH analysis along with the COG results for each case were reviewed to determine the ploidy status and the presence of recurrent rearrangements to support categorization of B-ALL subtypes. The main subtypes of B-ALL identified from these findings in this study include B-ALL with high hyperdiploidy (51-65 chromosomes with or without simultaneous trisomies 4 and 10), hypodiploidy (including near-haploidy, low-hypodiploidy and high hypodiploidy), intrachromosomal amplification of chromosome 21 (iAMP 21), *ETV6-RUNX1* fusion/t(12;21)(p13;q22), *BCR-ABL1* fusion/t(9;22)(q34;q11.2), *TCF3-PBX1* fusion/t(1;19)(q23;p13) or der(19)t(1;19), and *KMT2A* rearrangement/t(v;11q23.3)^92^. Other miscellaneous cytogenetic changes including complex karyotypes (3 structural abnormalities/clones) that did not fit in the above subtypes were recorded and were categorized as NOS (not otherwise specified) due to the limited information available. Rare subtypes (<15 patients) with insufficient sample size for further analysis included: hypodiploidy, *KMT2A* rearrangement, *BCR-ABL1* fusion, and iAMP21. Associations between specific risk variants of interest (carrying ≥1 risk allele versus none) and each subtype were tested using Fisher’s exact test (two-sided *P* < 0.05).

Outcome data were available for 397 COG participants in clinical trials not subject to current data embargoes. Participants were further classified their enrollment in COG standard (*n* = 165) versus high (*n* = 220) risk therapy protocols (based on their age at diagnosis, white blood cell count at presentation, and cytogenetics). We excluded 12 participants diagnosed during infancy (age <365 days) or with Philadelphia chromosome-positive disease since these individuals are treated on separate protocols and have different prognoses than other pediatric B-ALL subgroups. Overall survival was the endpoint of interest, with time at risk beginning at B-ALL diagnosis and ending at death or censoring at last follow-up. Overall survival probabilities were estimated with the Kaplan-Meier method for individuals carrying zero versus at least one candidate African ancestry-specific B-ALL risk allele and log-rank tests compared cumulative incidence curves. Cox proportional hazards regression compared mortality hazard rates between carriers and non-carriers overall, adjusting for sex, genetic ancestry, and enrollment in standard versus high risk COG treatment protocols.

### Reporting summary

Further information on research design is available in the Nature Portfolio Reporting Summary linked to this article.

## Supplementary information

Supplementary Information

Reporting Summary

Transparent Peer Review file

## Data Availability

Genotypes for cases and controls genotyped on the Global Diversity Array as part of this study are available for download at dbGaP (https://ncbi.nlm.nih.gov/gap/, data accession: phs004222.v1.p1). Access to individual-level data for ZOE 2.0 study participants^57^ are available through dbGaP (https://ncbi.nlm.nih.gov/gap/, data accession: phs002232.v1.p1). GWAS summary statistics are available in the NHGRI-EBI GWAS Catalog (https://www.ebi.ac.uk/gwas/, data accession: GCST90668017). Access to Childhood Cancer Record Linkage Project data are not publicly available due to California Department of Public Health regulations which restricts original data and summary statistics sharing; however, access may be granted by the Principal Investigators to bona fide researchers with the completion of data use agreements as previously described (https://www.nature.com/articles/s41375-021-01465-1). Data used in this study from the Children’s Oncology Group (COG) have been published previously; COG data may be requested following organizational procedures (https://childrensoncologygroup.org/data-sharing; as indicated on the website, decisions about data access are provided in writing within 6 weeks of receipt) by any investigator regardless of COG membership and accessed with a completed data use agreement, or through the NCTN Archive (https://nctn-data-archive.nci.nih.gov/). External chromatin looping datasets are publicly available and listed in Suppementary Table 10. Whole blood eQTL data published by Kachuri et al.^29^ were obtained from Zenodo (https://zenodo.org/, data accession: 7735723). All other data supporting the findings of this study are available in the article or the Supplementary Information files.

## Code availability

Software and analytical tools used in data analyses include: PLINK versions 1.9 and 2.0 (https://www.cog-genomics.org/plink/) as specified in the Methods for quality control, association testing, LD-clumping, and PRS calculations; RFMix^25^ (v2.03-r0, https://github.com/slowkoni/rfmix) for global and local ancestry inference; NHLBI Trans-Omics for Precision Medicine or TOPMed^62^ Imputation Server (https://imputation.biodatacatalyst.nhlbi.nih.gov) for imputation; PCAMatchR^67^ (https://cran.r-project.org/web/packages/PCAmatchR/index.html) for control matching; METAL^26^ (https://github.com/statgen/METAL) and METASOFT^70^ (http://genetics.cs.ucla.edu/meta_jemdoc/) for meta-analysis; ANNOVAR^73^ (https://annovar.openbioinformatics.org/en/latest/) for functional annotation; coloc R package^30^ (v5.2.3, https://github.com/chr1swallace/coloc) for colocalization analysis; GCTA (v1.94.1, https://yanglab.westlake.edu.cn/software/gcta/#GREMLanalysis) for heritability analysis; and R v.4.2.1 for all other statistical analyses and plotting (https://www.R-project.org).
