# Supplementary material for: Genome-wide association study of childhood B-cell acute lymphoblastic leukemia reveals novel African ancestry-specific susceptibility loci
Source: Nat Commun. 2025 Oct 22;16:8974. doi: 10.1038/s41467-025-64337-7 (PMC12546916; doi:10.1038/s41467-025-64337-7)
Supplement: Supplementary file 1 — Supplementary Information [file 41467_2025_64337_MOESM1_ESM.docx]

**SUPPLEMENTARY INFORMATION**

**SUPPLEMENTARY NOTE**

**Additional characterizations of index variants with any whole eQTL evidence**

Index variant rs115636216 showed associations in our discovery data (OR=2.90, P=2.2x10^-10^), consistent direction of association in the primary replication dataset (OR=1.53, P=0.28), and nominal replication in African American participants in the CCRLP study (OR=2.07, P=0.026). The index variant is an intronic SNV in the *NREP* (neuronal regeneration related protein) gene, which is reported to play a role in hematopoietic cell development.^1^ *NREP* expression has been identified as a predictor of pediatric acute myeloid leukemia blast-related prognostic features.^2^ The index variant risk allele is also associated with increased expression of *EPB41L4A* (erythrocyte protein band 4.1-like 4a) in whole blood in admixed populations.^3^ *EPB41L4A* is a target gene for the Wnt/β-catenin pathway, which plays a role in the survival of multiple myeloma (another hematological malignancy) cells.^4^

Index variant rs76135126 (discovery OR=2.03, P=2.5x10^-7^; replication OR=1.99, P=0.014) resides in the promoter region of *SLC37A4* (solute carrier family 37 member 4) and is also upstream of *VPS11* (vacuolar protein sorting 11). The rs76135126 risk allele is associated with increased expression of both of these genes in whole blood in admixed populations.^3^ Pathogenic variants in *SLC37A4* are linked to autosomal recessive glycogen storage disorder 1b (GSD1b, MIM: 232220), which is frequently accompanied by neutropenia and neutrophil dysfunction.^5^ *VPS11* has been implicated in leukemogenesis as a fusion partner of *KMT2a* in an insertional mutagenesis screen in a murine model.^6^ Furthermore, a credible set variant at this locus (rs77765196, pairwise LD with index variant in the 1000 Genomes, African population: r^2^=0.84) overlaps a DNase-seq peak in lymphoblastoid cells and a ChIP-seq peak for transcription factor Zeb2 in a leukemia cell line (K562). Zeb2 has been demonstrated to control hematopoietic differentiation and may contribute to blood disorders.^7^

The genomic region bearing index variant rs113299167 (discovery OR=2.00, P=5.2x10^-7^; replication OR=1.96, P=6.1x10^-3^) showed three-dimensional chromatin interactions with the promoter region of *TMEM132D* in hematopoietic tissue expressing CD34+ cells and B-cell lymphoblastoid cells (GM12878). In our own experiments, dual luciferase reporter assays identified significant allele-specific effects for this index variant with consistent directions of effect in GM12878 and leukemia (697) cell lines. This variant overlaps a ChIP-seq peak for zinc finger protein ZNF512 in a leukemia cell line (K562). ZNF512 has recently been reported to be sufficient to initiate pericentric heterochromatin formation, an essential biological function for maintaining genome stability.^8^

**SUPPLEMENTARY FIGURES**

COG cases (ADMIRAL)

Non-COG cases (ADMIRAL)

External controls (ZOE 2.0)

dbGaP phs002232.v1.p1

Study controls (ADMIRAL)

4:1 matched controls to cases

(sex, finer ancestry)

**Discovery data**

(N=3,280, 656 cases)

**Replication data**

(N=920, 184 cases)

**Evaluable genetic variants:**

11,876,503 (MAF ≥1%)

**Post-QC and imputation**

Non-COG case sources: Michigan BioTrust for Health, Baylor, UAB, UTSW, CHOP, MSKCC

Control sources:

- ZOE 2.0, N=6,144
- Study controls from Michigan BioTrust for Health, Baylor (N=574)

**Supplementary Figure 1**: Overview of sources of cases and controls for B-ALL GWAS meta-analysis.


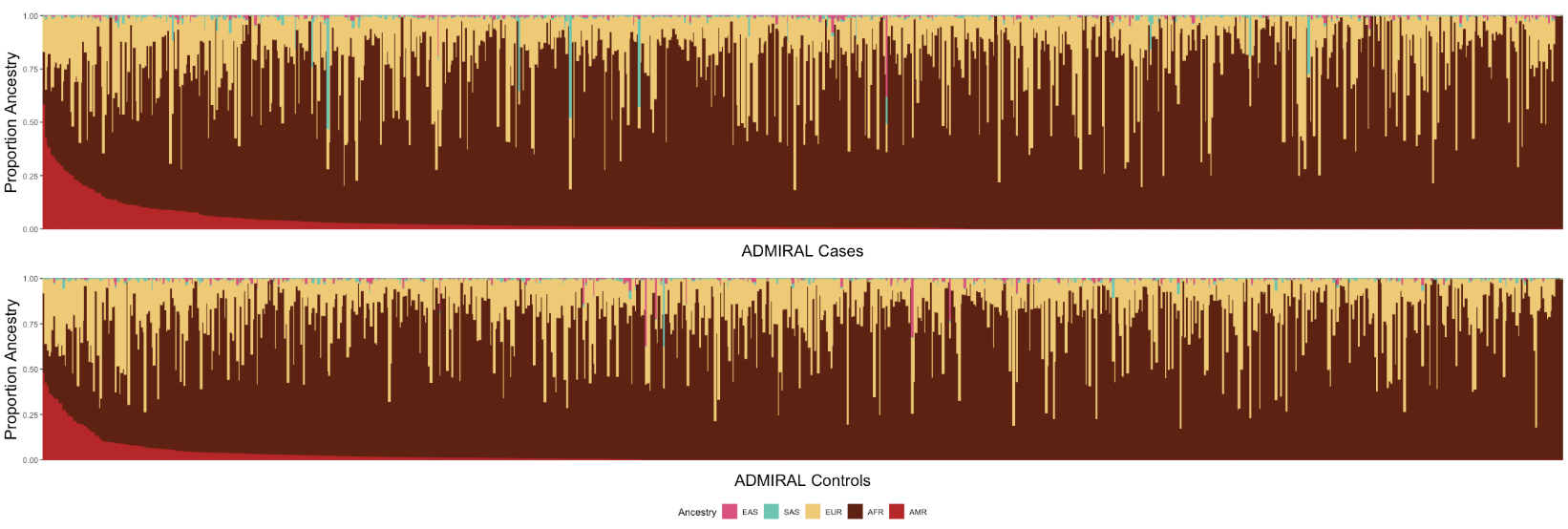

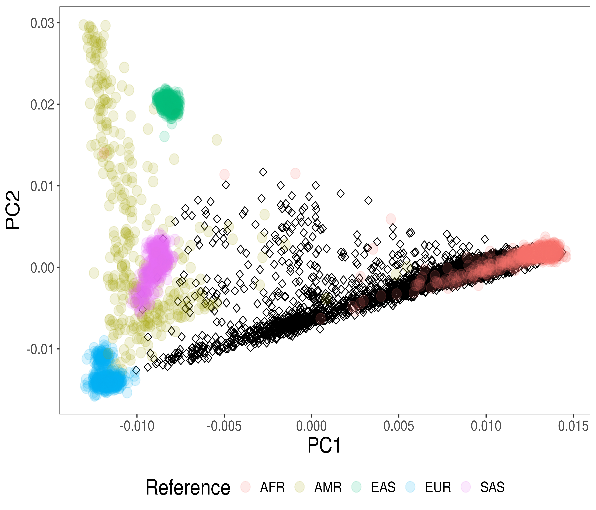

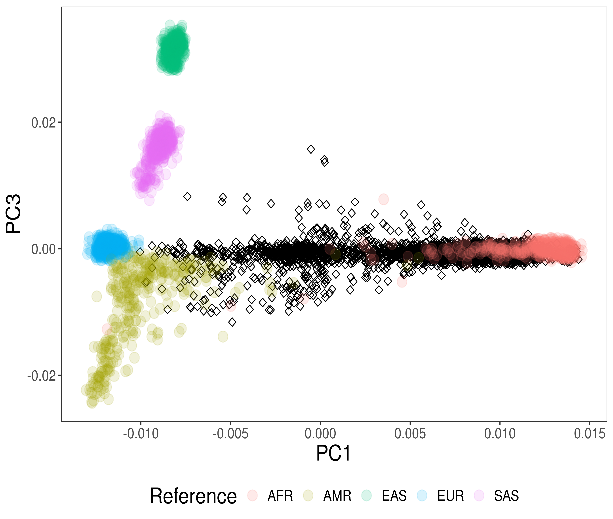

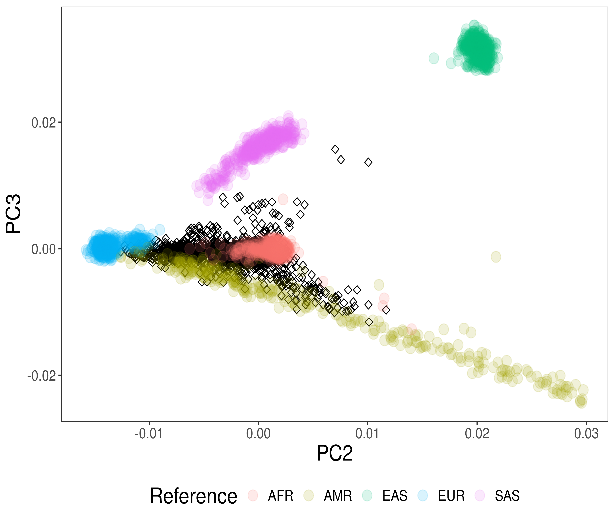


**Supplementary Figure 2:** Ancestry eigenvector biplot and inferred global ancestry in ADMIRAL cases (N=840) and controls (N=3,360). Above, plots of the first three principal components with 1000 Genomes ancestral reference populations (colored circles) with ADMIRAL study participants with African global ancestry values meeting the analysis threshold (black-outlined squares) are shown. Participants in the 1000 Genomes African ancestry (AFR) reference panel are shown in light-red circles. Below, RFMix global ancestry proportions in cases (top) and controls (bottom) are shown.


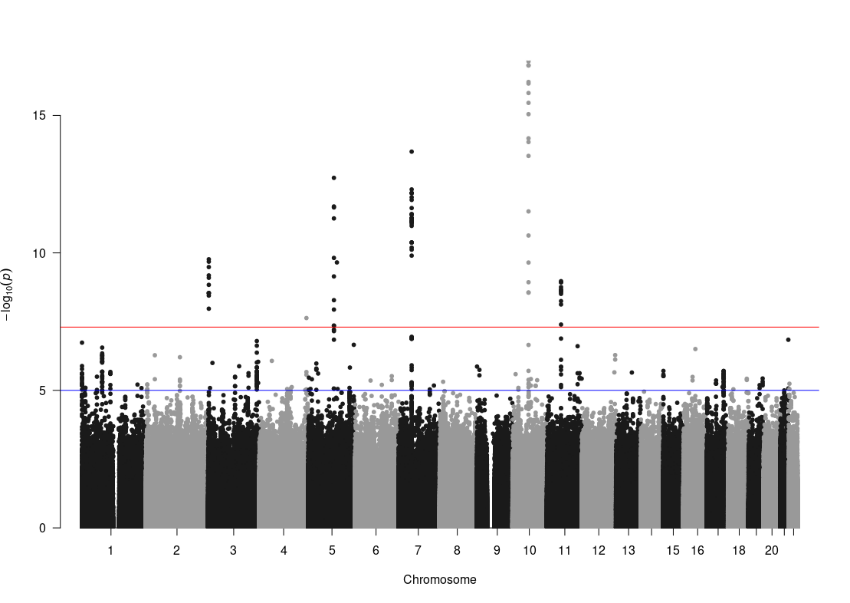

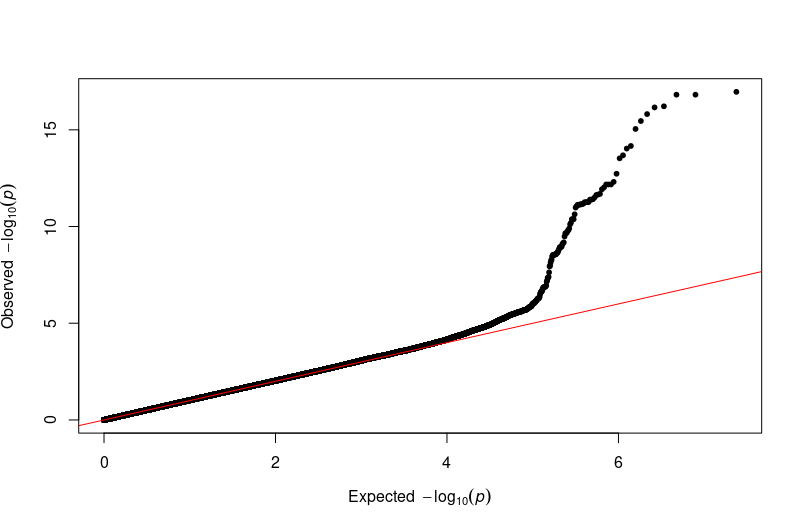


*CLDN24*

lambda_GC_=1.03

*IKZF1*

*ARID5B*

*ORC46*

*FAM174A NREP*

*CNTN4*

λ=1.03

**Supplementary Figure 3**: Manhattan plot and quantile-quantile (Q-Q) plot with variant association test p-values from the B-ALL discovery GWAS (N=3,280). The Manhattan plot (top) illustrates –log_10_ p-values for variant associations with B-ALL risk (y-axis) against variant genomic positions (x-axis). The solid red line signifies the genome-wide significance threshold (log-transformed P=5x10^-8^). The QQ plot shows observed –log10 p-values (y-axis) against those expected under the null distribution of no association (x-axis), as well as the genomic inflation factor (lambda).


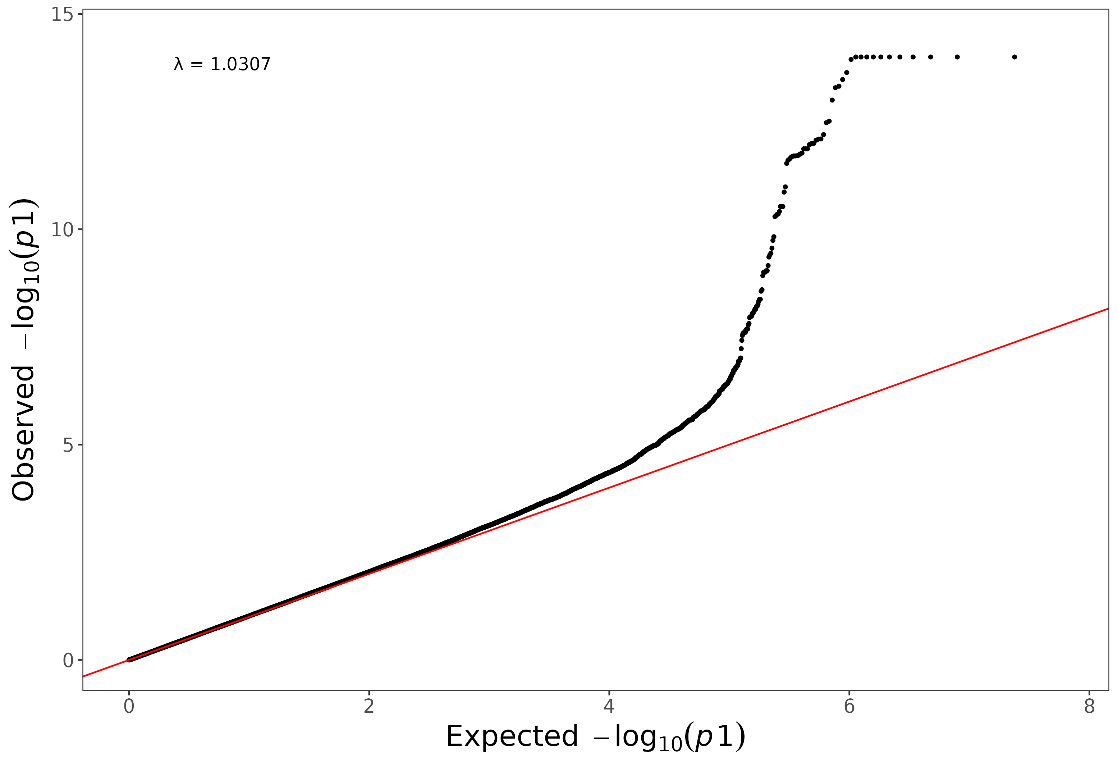


**Supplementary Figure 4**: Quantile-quantile (Q-Q) plot with variant association test p-values from the B-ALL GWAS meta-analysis (N=4,200). The Q-Q plot shows observed –log10 p-values (y-axis) against those expected under the null distribution of no association (x-axis), as well as the genomic inflation factor (lambda).

**Supplementary Figure 5**: LocusZoom plots of novel ancestry-specific childhood B-ALL risk loci with genome-wide significant associations after B-ALL GWAS meta-analysis in African American children. Single variant association test p-values from discovery analyses are shown (-log_10_ scale) for variants up to 3-megabases near the index variant at each locus (annotated in bottom right corner of each panel). SNPs are color coded by their magnitude of linkage disequilibrium (LD, measured as r^2^) with the most significantly associated variant in the window in the ADMIRAL discovery data set.


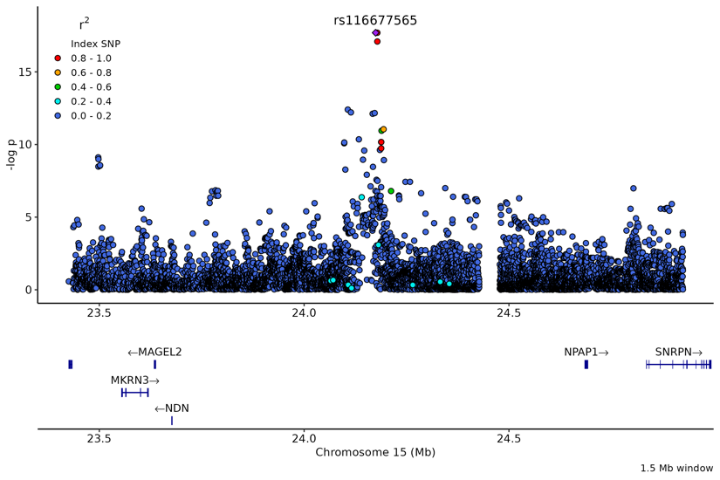

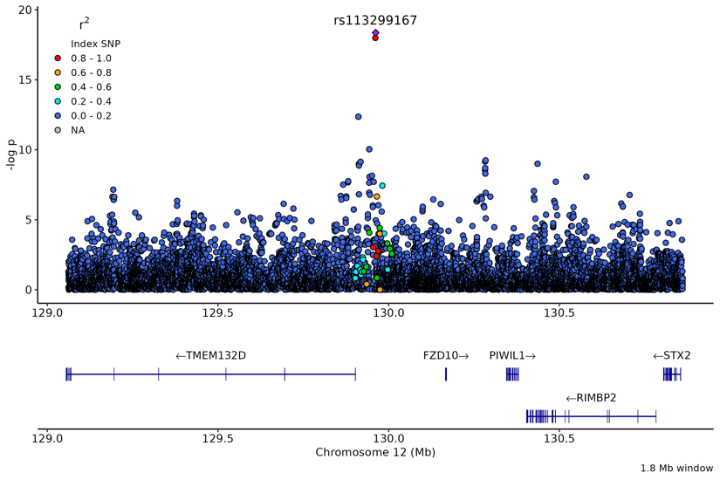

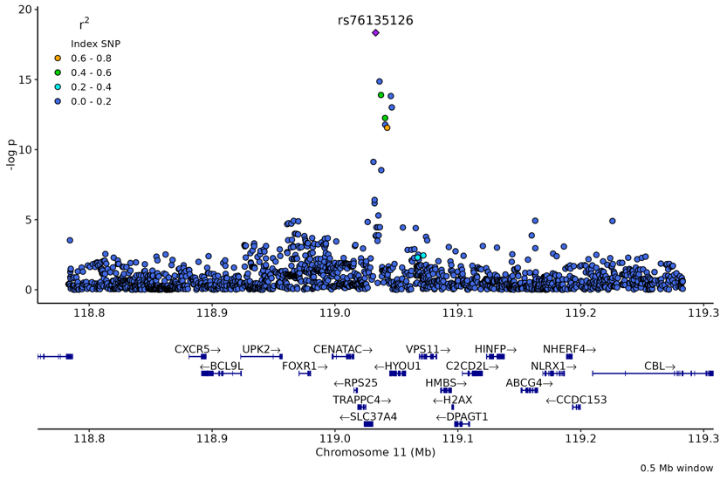

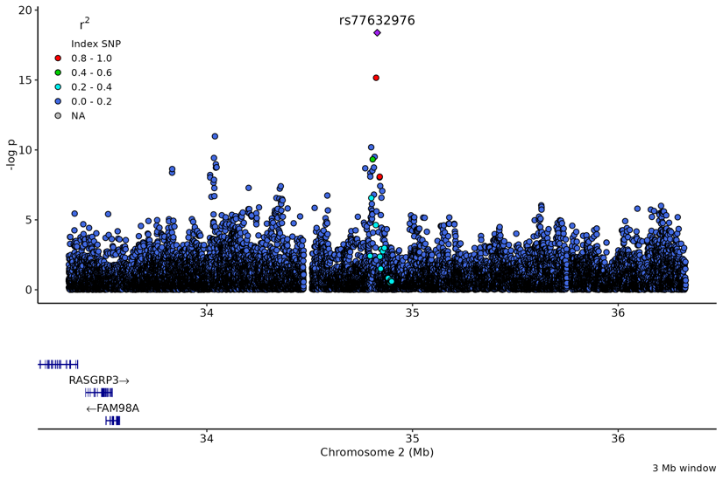

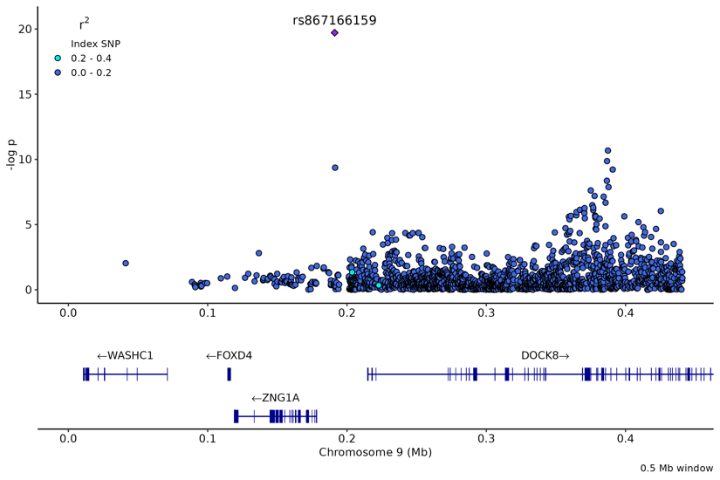

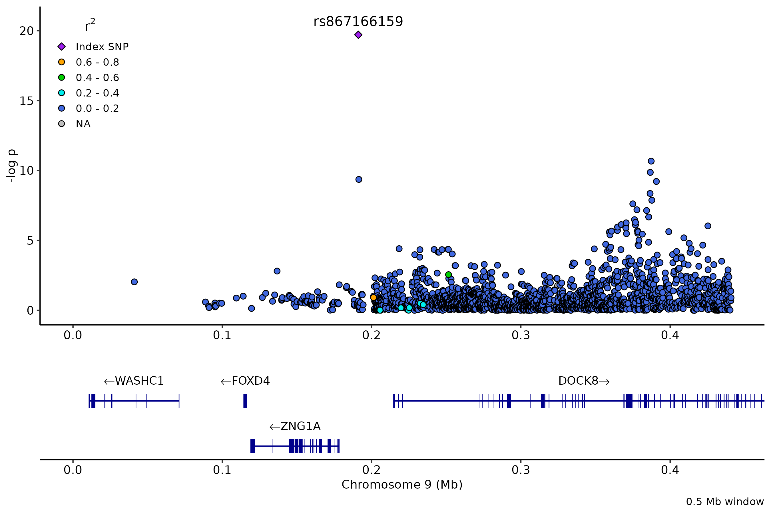

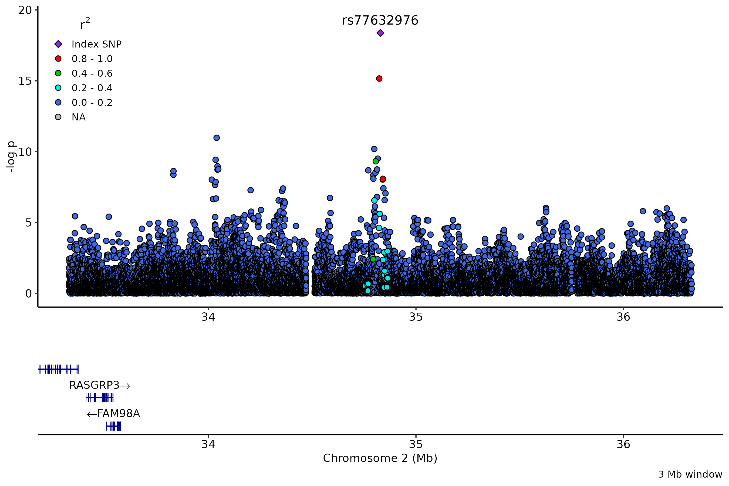


**Supplementary Figure 6**: LocusZoom plots of two novel ancestry-specific childhood B-ALL risk loci (rs77632976, chromosome 2) and (rs867166159, chromosome 9) with reference panel linkage disequilibrium values. Single variant association test p-values from discovery analyses are shown (-log_10_ scale) for variants up to 3-megabases near the index variant at each locus (annotated in bottom right corner of each panel). SNPs are color coded by their magnitude of linkage disequilibrium (LD, measured as r^2^) with the most significantly associated variant in the window in the 1000 Genomes African ancestry reference panel.

**SUPPLEMENTARY TABLES**

**Supplementary Table 1**: Description of sources for B-ALL cases and controls for the ADMIRAL study

| Source | Number of cases in analysis | Number of controls in analysis | DNA specimen type(s) |
| --- | --- | --- | --- |
| Children’s Oncology Group (COG)  Protocols: 9904, 9905, 9906, AALL1131, AALL15P1, AALL1621, AALL0232, APEC14B1 | 656 | 0 | Blood or bone marrow |
| Michigan Biotrust for Health | 112 | 362 | Newborn dried bloodspots |
| Baylor College of Medicine | 33 | 30 | Blood or saliva |
| University of Alabama at Birmingham (UAB) | 9 | 0 | Blood |
| University of Texas Southwestern (UTSW) | 5 | 0 | Blood or saliva |
| Children’s Hospital of Philadelphia (CHOP) | 18 | 0 | Blood or bone marrow |
| Memorial Sloan Kettering Cancer Center (MSKCC) | 7 | 0 | Blood or bone marrow |

**Supplementary Table 2**: Sex of African American children with B-ALL included in ADMIRAL GWAS

|  | Discovery data, with cases from COG (N=656) | Replication data, with cases from non-COG sources (N=184) |
| --- | --- | --- |
| Characteristic | % (n) | % (n) |
| Sex |  |  |
| Female | 45.9% (301) | 45.7% (84) |
| Male | 54.1% (355) | 54.3% (100) |

**Supplementary Table 3:** All variants with genome-wide significant p-values in discovery or after meta-analysis, including corresponding locus variants in linkage disequilibrium if replicated (r^2^>0.5, 100-kb window, P<1x10^-3^ in discovery)

|  |  |  |  |  |  |  |  | Meta-analysis | | | Discovery | | Replication | | | 1000 Genomes 30X | | | | |
| --- | --- | --- | --- | --- | --- | --- | --- | --- | --- | --- | --- | --- | --- | --- | --- | --- | --- | --- | --- | --- |
| SNP | CHR | BP | NEA | EA | EAF_case_ | EAF_ctrl_ | Mean Rsq | OR | P | Phet | OR | P | | OR | P | AFR | AMR | EAS | EUR | SAS |
| rs79191888 | 2 | 34823085 | G | A | 0.08 | 0.05 | 0.97 | 1.73  (1.53-1.94) | 2.6E-07 | 0.79 | 1.76  (1.39-2.24) | 3.9E-06 | | 1.65  (1.08-2.52) | 0.021 | 0.09 | 0.003 | 0 | 0 | 0 |
| **rs77632976** | 2 | 34828430 | C | T | 0.08 | 0.04 | 0.96 | 1.87  (1.66-2.09) | 1.0E-08 | 0.98 | 1.87  (1.47-2.39) | 5.3E-07 | | 1.86  (1.2-2.9) | 5.8E-03 | 0.08 | 0.003 | 0 | 0 | 0 |
| **rs112113758** | 3 | 2292232 | G | T | 0.08 | 0.04 | 0.90 | 2.09  (1.88-2.31) | 1.4E-11 | 0.38 | 2.20  (1.73-2.81) | 1.7E-10 | | 1.75  (1.1-2.77) | 0.017 | 0.09 | 0.009 | 0 | 0 | 0 |
| rs113085788 | 3 | 2293659 | G | C | 0.08 | 0.04 | 0.91 | 2.07  (1.86-2.29) | 3.9E-11 | 0.52 | 2.15  (1.69-2.75) | 8.0E-10 | | 1.81  (1.14-2.88) | 0.012 | 0.08 | 0.007 | 0 | 0 | 0 |
| rs112398377 | 3 | 2294705 | T | C | 0.08 | 0.04 | 0.91 | 2.12  (1.91-2.34) | 1.0E-11 | 0.45 | 2.22  (1.74-2.84) | 2.1E-10 | | 1.82  (1.15-2.88) | 0.011 | 0.08 | 0.007 | 0 | 0 | 0 |
| rs116156867 | 3 | 2298834 | T | C | 0.08 | 0.04 | 0.93 | 2.01  (1.8-2.23) | 1.5E-10 | 0.40 | 2.11  (1.66-2.69) | 1.5E-09 | | 1.70  (1.07-2.68) | 0.024 | 0.08 | 0.007 | 0 | 0 | 0 |
| **rs28568357** | 4 | 183347814 | T | C | 0.06 | 0.04 | 0.99 | ++ | 3.2E-07 | 0.013 | 2.15  (1.64-2.81) | 2.3E-08 | | 1.02  (0.60-1.72) | 0.95 | 0.05 | 0.009 | 0 | 0.02 | 0.007 |
| rs375754950 | 5 | 100055387 | G | A | 0.03 | 0.01 | 0.80 | 2.71  (2.34-3.08) | 1.1E-07 | 0.66 | 2.59  (1.7-3.94) | 8.6E-06 | | 3.15  (1.46-6.82) | 3.6E-03 | 0.05 | 0.001 | 0 | 0.001 | 0 |
| rs1172954145 | 5 | 100062811 | A | C | 0.03 | 0.01 | 0.80 | 2.71  (2.34-3.08) | 1.1E-07 | 0.66 | 2.59  (1.7-3.94) | 8.7E-06 | | 3.16  (1.46-6.83) | 3.5E-03 | 0.05 | 0.001 | 0 | 0.002 | 0 |
| rs9686653 | 5 | 100067680 | A | G | 0.03 | 0.01 | 0.80 | 2.41  (2.05-2.77) | 1.3E-06 | 0.48 | 2.25  (1.5-3.38) | 9.9E-05 | | 3.04  (1.46-6.37) | 3.1E-03 | 0.05 | 0.003 | 0 | 0.003 | 0 |
| rs182617065 | 5 | 100088462 | T | C | 0.05 | 0.02 | 0.86 | 2.87  (2.58-3.17) | 2.4E-12 | 0.73 | 2.80  (2.02-3.89) | 7.2E-10 | | 3.19  (1.62-6.29) | 7.7E-04 | 0.06 | 0.003 | 0 | 0.001 | 0 |
| rs7731907 | 5 | 100095464 | T | C | 0.06 | 0.02 | 0.89 | 2.82  (2.55-3.09) | 2.3E-14 | 0.69 | 2.90  (2.15-3.9) | 2.2E-12 | | 2.52  (1.38-4.6) | 2.5E-03 | 0.06 | 0.004 | 0 | 0.001 | 0 |
| **rs183221417** | 5 | 100102043 | T | C | 0.06 | 0.02 | 0.90 | 2.94  (2.68-3.21) | 1.2E-15 | 0.65 | 3.04  (2.26-4.08) | 1.9E-13 | | 2.60  (1.44-4.68) | 1.5E-03 | 0.06 | 0.004 | 0 | 0.001 | 0 |
| rs61526151 | 5 | 100109217 | C | A | 0.05 | 0.02 | 0.89 | 3.03  (2.74-3.32) | 5.2E-14 | 0.69 | 3.12  (2.26-4.32) | 5.6E-12 | | 2.70  (1.43-5.12) | 2.3E-03 | 0.06 | 0.003 | 0 | 0.001 | 0 |
| rs76291187 | 5 | 100127437 | C | T | 0.05 | 0.02 | 0.93 | 2.88  (2.60-3.16) | 1.0E-13 | 0.50 | 3.01  (2.21-4.09) | 2.1E-12 | | 2.34  (1.21-4.53) | 0.012 | 0.06 | 0.003 | 0 | 0.001 | 0 |
| **rs115636216** | 5 | 111791133 | G | A | 0.04 | 0.02 | 0.96 | 2.63  (2.32-2.93) | 3.9E-10 | 0.13 | 2.9  (2.09-4.04) | 2.2E-10 | | 1.53  (0.71-3.28) | 0.28 | 0.04 | 0.001 | 0 | 0 | 0 |
| -- | 7 | 50394709 | T | A | 0.30 | 0.21 | 0.99 | 1.56  (1.44-1.68) | 8.0E-13 | 0.21 | 1.62  (1.41-1.86) | 3.9E-12 | | 1.34  (1.03-1.75) | 0.031 |  |  |  |  |  |
| rs62447205 | 7 | 50398132 | A | G | 0.30 | 0.21 | 1.00 | 1.56  (1.44-1.68) | 8.5E-13 | 0.21 | 1.62  (1.41-1.86) | 4.1E-12 | | 1.34  (1.03-1.75) | 0.031 | 0.18 | 0.23 | 0.12 | 0.32 | 0.30 |
| rs11978267 | 7 | 50398606 | A | G | 0.30 | 0.21 | 1.00 | 1.56  (1.44-1.68) | 8.1E-13 | 0.22 | 1.62  (1.41-1.86) | 4.1E-12 | | 1.34  (1.03-1.75) | 0.029 | 0.18 | 0.23 | 0.12 | 0.32 | 0.32 |
| rs6973210 | 7 | 50398997 | G | A | 0.30 | 0.21 | 1.00 | 1.55  (1.43-1.67) | 1.1E-12 | 0.22 | 1.62  (1.41-1.85) | 5.4E-12 | | 1.34  (1.03-1.75) | 0.031 | 0.18 | 0.23 | 0.12 | 0.32 | 0.30 |
| rs6960400 | 7 | 50399099 | A | G | 0.30 | 0.21 | 1.00 | 1.55  (1.43-1.68) | 1.0E-12 | 0.22 | 1.62  (1.41-1.85) | 5.4E-12 | | 1.34  (1.03-1.75) | 0.029 | 0.18 | 0.23 | 0.12 | 0.32 | 0.30 |
| -- | 7 | 50401254 | G | T | 0.29 | 0.21 | 1.00 | 1.54  (1.42-1.66) | 3.0E-12 | 0.20 | 1.61  (1.4-1.84) | 1.0E-11 | | 1.32  (1.01-1.72) | 0.040 |  |  |  |  |  |
| rs11552047 | 7 | 50401853 | C | T | 0.29 | 0.21 | 1.00 | 1.54  (1.42-1.67) | 2.5E-12 | 0.19 | 1.61  (1.4-1.85) | 8.8E-12 | | 1.32  (1.01-1.72) | 0.040 | 0.18 | 0.23 | 0.11 | 0.32 | 0.29 |
| rs11980379 | 7 | 50402283 | T | C | 0.29 | 0.21 | 1.00 | 1.55  (1.43-1.67) | 2.0E-12 | 0.19 | 1.61  (1.41-1.85) | 7.5E-12 | | 1.32  (1.01-1.72) | 0.039 | 0.18 | 0.23 | 0.11 | 0.32 | 0.29 |
| rs4132601 | 7 | 50402906 | T | G | 0.29 | 0.21 | 1.00 | 1.55  (1.43-1.67) | 2.0E-12 | 0.19 | 1.61  (1.41-1.85) | 7.5E-12 | | 1.32  (1.01-1.72) | 0.039 | 0.18 | 0.23 | 0.11 | 0.32 | 0.29 |
| rs11980407 | 7 | 50403915 | G | A | 0.29 | 0.21 | 1.00 | 1.55  (1.43-1.67) | 2.0E-12 | 0.19 | 1.62  (1.41-1.85) | 6.8E-12 | | 1.32  (1.01-1.73) | 0.042 | 0.18 | 0.23 | 0.11 | 0.32 | 0.29 |
| rs62445866 | 7 | 50404626 | G | A | 0.29 | 0.21 | 0.99 | 1.55  (1.43-1.67) | 1.9E-12 | 0.18 | 1.62  (1.41-1.86) | 6.6E-12 | | 1.32  (1.01-1.73) | 0.042 | 0.18 | 0.23 | 0.11 | 0.32 | 0.29 |
| rs58923657 | 7 | 50405144 | C | T | 0.29 | 0.21 | 0.99 | 1.55  (1.43-1.67) | 2.2E-12 | 0.19 | 1.62  (1.41-1.85) | 7.2E-12 | | 1.32  (1.01-1.73) | 0.042 | 0.18 | 0.23 | 0.11 | 0.32 | 0.29 |
| rs6964969 | 7 | 50405553 | A | G | 0.29 | 0.21 | 1.00 | 1.55  (1.43-1.67) | 2.0E-12 | 0.20 | 1.62  (1.41-1.85) | 7.2E-12 | | 1.32  (1.01-1.73) | 0.039 | 0.18 | 0.23 | 0.11 | 0.32 | 0.29 |
| rs6956014 | 7 | 50405592 | T | C | 0.29 | 0.21 | 0.99 | 1.55  (1.43-1.67) | 1.7E-12 | 0.18 | 1.62  (1.41-1.86) | 5.6E-12 | | 1.32  (1.01-1.73) | 0.042 | 0.17 | 0.21 | 0.05 | 0.25 | 0.22 |
| rs114373434 | 7 | 50407233 | C | G | 0.22 | 0.14 | 1.00 | 1.71  (1.57-1.84) | 4.8E-14 | 0.27 | 1.77  (1.52-2.07) | 4.9E-13 | | 1.46  (1.07-1.99) | 0.016 | 0.09 | 0.23 | 0.11 | 0.31 | 0.29 |
| rs62445869 | 7 | 50409913 | G | A | 0.22 | 0.14 | 1.00 | 1.67  (1.53-1.81) | 3.4E-13 | 0.16 | 1.75  (1.50-2.05) | 9.5E-13 | | 1.37  (1.00-1.87) | 0.046 | 0.09 | 0.23 | 0.11 | 0.32 | 0.29 |
| **rs17133807** | 7 | 50409989 | G | A | 0.28 | 0.19 | 1.00 | 1.62  (1.49-1.74) | 3.3E-14 | 0.05 | 1.72  (1.5-1.98) | 2.1E-14 | | 1.27  (0.96-1.67) | 0.093 | 0.16 | 0.23 | 0.11 | 0.32 | 0.29 |
| **rs867166159** | 9 | 191139 | G | T | 0.04 | 0.02 | 0.84 | 2.69  (2.36-3.02) | 2.7E-09 | 0.46 | 2.51  (1.73-3.65) | 1.4E-06 | | 3.36  (1.72-6.55) | 3.9E-04 | 0.05 | 0.001 | 0 | 0 | 0 |
| rs7073837 | 10 | 61940136 | C | A | 0.39 | 0.29 | 1.00 | 1.56  (1.33-1.92) | 6.6E-15 | 0.069 | 1.67  (1.37-2.13) | 9.3E-15 | | 1.28  (1.00-1.64) | 0.045 | 0.26 | 0.51 | 0.36 | 0.39 | 0.63 |
| rs10740053 | 10 | 61942825 | T | C | 0.35 | 0.24 | 0.99 | ++ | 1.6E-17 | 0.044 | 1.79  (1.45-2.33) | 1.5E-17 | | 1.33  (1.03-1.72) | 0.025 | 0.22 | 0.47 | 0.33 | 0.31 | 0.51 |
| rs10740054 | 10 | 61942827 | A | T | 0.35 | 0.24 | 0.99 | ++ | 1.5E-17 | 0.045 | 1.79  (1.45-2.33) | 1.5E-17 | | 1.33  (1.04-1.72) | 0.024 | 0.22 | 0.47 | 0.33 | 0.31 | 0.51 |
| rs4948492 | 10 | 61959980 | T | C | 0.34 | 0.23 | 0.99 | 1.67  (1.39-2.08) | 1.9E-17 | 0.073 | 1.79  (1.43-2.33) | 6.9E-17 | | 1.37  (1.05-1.75) | 0.017 | 0.20 | 0.47 | 0.36 | 0.33 | 0.52 |
| **rs7090445** | 10 | 61961417 | T | C | 0.33 | 0.22 | 1.00 | 1.72  (1.41-2.17) | 3.1E-18 | 0.066 | 1.82  (1.45-2.44) | 1.1E-17 | | 1.37  (1.06-1.79) | 0.016 | 0.19 | 0.47 | 0.36 | 0.33 | 0.52 |
| rs4245595 | 10 | 61963136 | T | C | 0.34 | 0.24 | 1.00 | 1.67  (1.39-2.08) | 2.1E-17 | 0.066 | 1.79  (1.43-2.33) | 6.1E-17 | | 1.35  (1.05-1.75) | 0.019 | 0.20 | 0.47 | 0.36 | 0.33 | 0.52 |
| rs10821936 | 10 | 61963818 | T | C | 0.34 | 0.24 | 0.99 | 1.67  (1.39-2.08) | 6.7E-17 | 0.063 | 1.75  (1.43-2.33) | 1.5E-16 | | 1.33  (1.04-1.72) | 0.024 | 0.20 | 0.47 | 0.36 | 0.33 | 0.52 |
| rs10821937 | 10 | 61964150 | T | C | 0.34 | 0.23 | 0.99 | 1.67  (1.39-2.08) | 8.9E-17 | 0.084 | 1.75  (1.41-2.27) | 3.5E-16 | | 1.35  (1.05-1.75) | 0.018 | 0.19 | 0.47 | 0.36 | 0.33 | 0.52 |
| rs4245597 | 10 | 61966183 | A | G | 0.29 | 0.20 | 0.98 | 1.64  (1.37-2.08) | 1.1E-14 | 0.11 | 1.72  (1.39-2.33) | 3.0E-14 | | 1.35  (1.02-1.79) | 0.035 | 0.16 | 0.48 | 0.36 | 0.33 | 0.51 |
| rs4506592 | 10 | 61967428 | G | A | 0.40 | 0.31 | 0.99 | 1.49  (1.28-1.79) | 1.8E-12 | 0.35 | 1.54  (1.28-1.92) | 2.3E-11 | | 1.35  (1.06-1.72) | 0.014 | 0.30 | 0.49 | 0.35 | 0.35 | 0.52 |
| rs10761598 | 10 | 61980101 | A | T | 0.39 | 0.30 | 0.98 | 1.47  (1.25-1.75) | 5.1E-11 | 0.55 | 1.49  (1.25-1.85) | 1.2E-09 | | 1.37  (1.08-1.75) | 0.011 | 0.28 | 0.49 | 0.35 | 0.35 | 0.52 |
| **rs112269413** | 11 | 54651552 | G | A | 0.04 | 0.02 | 0.90 | 2.69  (2.38-3.00) | 4.4E-10 | 0.24 | 2.96  (2.09-4.20) | 1.1E-09 | | 1.86  (0.93-3.69) | 0.077 | 0.05 | 0.009 | 0 | 0 | 0 |
| **rs76135126** | 11 | 119033074 | T | C | 0.06 | 0.03 | 0.89 | 2.02  (1.78-2.26) | 1.1E-08 | 0.95 | 2.03  (1.55-2.66) | 2.5E-07 | | 1.99  (1.15-3.44) | 0.014 | 0.08 | 0.006 | 0 | 0 | 0 |
| rs77765196 | 11 | 119042468 | C | G | 0.05 | 0.03 | 0.92 | 1.78  (1.52-2.03) | 9.5E-06 | 0.68 | 1.73  (1.30-2.30) | 1.7E-04 | | 1.97  (1.13-3.45) | 0.017 | 0.06 | 0.006 | 0 | 0 | 0 |
| rs113399637 | 12 | 129960984 | C | T | 0.06 | 0.03 | 0.95 | 1.98  (1.74-2.22) | 1.5E-08 | 0.96 | 1.99  (1.51-2.61) | 7.6E-07 | | 1.96  (1.21-3.17) | 6.1E-03 | 0.07 | 0.003 | 0 | 0 | 0 |
| **rs113299167** | 12 | 129961841 | C | T | 0.06 | 0.03 | 0.95 | 1.99  (1.76-2.23) | 1.1E-08 | 0.94 | 2.00  (1.53-2.62) | 5.2E-07 | | 1.96  (1.21-3.17) | 6.1E-03 | 0.07 | 0.003 | 0 | 0 | 0 |
| **rs116677565** | 15 | 24174608 | T | C | 0.04 | 0.02 | 0.90 | 2.39  (2.08-2.69) | 2.1E-08 | 0.80 | 2.33  (1.63-3.33) | 3.0E-06 | | 2.54  (1.41-4.58) | 1.9E-03 | 0.05 | 0.003 | 0 | 0 | 0 |
| rs5002270 | 15 | 24178668 | G | A | 0.04 | 0.02 | 0.90 | 2.39  (2.08-2.69) | 2.1E-08 | 0.80 | 2.33  (1.63-3.33) | 3.0E-06 | | 2.54  (1.41-4.58) | 1.9E-03 | 0.05 | 0.003 | 0 | 0 | 0 |
| rs145683427 | 15 | 24178703 | C | A | 0.04 | 0.02 | 0.90 | 2.42  (2.11-2.74) | 3.8E-08 | 0.36 | 2.22  (1.54-3.2) | 2.0E-05 | | 3.11  (1.68-5.76) | 3.2E-04 | 0.05 | 0.003 | 0 | 0 | 0 |

Abbreviations: CHR, chromosome; BP, base position, GRCh38 (hg38) build; EA, effect (risk) allele; NEA, non-effect (reference) allele; EAF, effect allele frequency; Rsq, imputation quality metric; OR, odds ratio; CI, confidence interval; P, p-value. Index variants are bolded. For any variant showing meta-analysis allelic heterogeneity (P_het_<0.05), p-values were estimated using the Han-Eskin random-effects model (RE2) and directions of effect in discovery and replication data are provided. Odds ratios (ORs) and 95% confidence intervals (CIs) are from logistic regression models. All presented p-values are from two-sided statistical tests and are not adjusted for multiple testing.

**Supplementary Table 4**: Previously identified ALL risk variants and risk associations in ADMIRAL

|  |  |  |  | Vijayakrishnan et al.^9^ European meta-analysis (2019) | | Jeon et al.^10^ trans-ethnic meta-analysis (2022) | | ADMIRAL meta-analysis  (N=4200, 840 cases) | | | | 1000 Genomes 30X EAF | | | | |
| --- | --- | --- | --- | --- | --- | --- | --- | --- | --- | --- | --- | --- | --- | --- | --- | --- |
| SNP^a^ | Gene | Chr:BP (hg38) | EA | OR | P | OR | P | OR | P | EAF (cases) | EAF  (ctrl) | AFR | AMR | EAS | EUR | SAS |
| **rs17481869^9^** | *2q22.3* | 2:145366886 | A | 1.74 | 2.4x10^-9^ | - | - | 0.99 | 0.99 | 0.02 | 0.02 | 0.01 | 0.04 | 0.00 | 0.08 | 0.03 |
| **rs11750693** | *5q31.2* | 5:137678942 | C | 2.62 | 4.1x10^-9^ | - | - | - | - | - | - | <0.01 | <0.01 | 0.00 | 0.01 | 0.01 |
| **rs886285^9^** | *C5orf56* | 5:132429514 | C^b^ | 1.29 | 1.6x10^-8^ | 0.99 | 0.72 | 0.97 | 0.58 | 0.37 | 0.37 | 0.28 | 0.70 | 0.35 | 0.66 | 0.48 |
| **rs210143^9^** | *BAK1* | 6:33579153 | C | 1.30 | 2.2x10^-8^ | 1.20 | 4.7x10^-8^ | 1.04 | 0.52 | 0.75 | 0.74 | 0.76 | 0.76 | 0.83 | 0.73 | 0.87 |
| rs9376090^10^ | *MYB/HBS1L* | 6:135090090 | C | - | - | 1.27 | 8.2x10^-9^ | 0.92 | 0.48 | 0.05 | 0.06 | 0.02 | 0.14 | 0.45 | 0.26 | 0.11 |
| **rs17133805^9^** | *IKZF1* | 7:50409816 | G | 1.65 | 5.3x10^-71^ | - | - | 1.57 | 1.4x10^-12^ | 0.27 | 0.19 | 0.15 | 0.23 | 0.11 | 0.32 | 0.29 |
| rs4132601^11^ | *IKZF1* | 7:50402906 | G | - | - | 1.43 | 1.4x10^-33^ | 1.55 | 2.0x10^-12^ | 0.29 | 0.21 | 0.18 | 0.23 | 0.11 | 0.32 | 0.29 |
| **rs74452384** | *7q36.3* | 7:157099824 | G | 3.97 | 3.2x10^-8^ | - | - | 1.05 | 0.47 | 0.82 | 0.81 | 0.79 | 0.99 | 0.99 | 0.99 | 0.94 |
| **rs75777619^9^** | *8q24* | 8:129172930 | G | 1.26 | 2.3x10^-9^ | - | - | 1.25 | 0.01 | 0.11 | 0.09 | 0.07 | 0.07 | 0.04 | 0.12 | 0.19 |
| rs4617118^12^ | *8q24* | 8:129143897 | G | - | - | 1.29 | 2.7x10^-12^ | 1.11 | 0.07 | 0.31 | 0.29 | 0.30 | 0.12 | 0.09 | 0.16 | 0.26 |
| **rs113650570^9^** | *CDKN2A* | 9:21976403 | A | 2.32 | 8.1x10^-35^ | - | - | - | - | - | - | 0.00 | 0.01 | 0.00 | 0.03 | 0.00 |
| rs3731249^13^ | *CDKN2A* | 9:21970917 | T | - | - | 1.95 | 2.2x10^-18^ | - | - | - | - | <0.01 | 0.01 | 0.00 | 0.03 | 0.00 |
| **rs76925697^9^** | *TLE1* | 9:81132456 | T^c^ | 1.52 | 2.1x10^-8^ | 0.85 | 0.059 | 0.77 | 0.10 | 0.03 | 0.04 | 0.03 | 0.03 | 0.00 | 0.04 | 0.02 |
| **rs3824662^14^** | *GATA3* | 10:8062245 | A | 1.29 | 3.6x10^-14^ | 1.21 | 1.8x10^-9^ | 1.24 | 9.4x10^-3^ | 0.14 | 0.11 | 0.08 | 0.37 | 0.27 | 0.19 | 0.17 |
| **rs2296624^9^** | *PIP4K2A* | 10:22568017 | C | 1.25 | 2.8x10^-15^ | - | - | 1.17 | 0.02 | 0.77 | 0.74 | 0.74 | 0.84 | 0.63 | 0.64 | 0.82 |
| rs11591377^15^ | *BMI1* | 10:22134373 | A | - | - | 0.80 | 9.6x10^-10^ | 0.86 | 0.12 | 0.09 | 0.10 | 0.07 | 0.26 | 0.05 | 0.21 | 0.11 |
| rs7088318^16^ | *PIP4K2A* | 10:22564019 | A | - | - | 1.32 | 4.6x10^-19^ | 1.31 | 2.0x10^-6^ | 0.47 | 0.41 | 0.34 | 0.75 | 0.61 | 0.60 | 0.77 |
| **rs10821936^17^** | *ARID5B* | 10:61963818 | T^d^ | 1.80 | 1.2x10^-106^ | 0.61 | 7.9x10^-69^ | 0.60 | 6.7x10^-17^ | 0.66 | 0.77 | 0.80 | 0.53 | 0.64 | 0.67 | 0.49 |
| rs9415680^10^ | *NRBF2/JMJD1C* | 10:63261130 | A | - | - | 1.20 | 7.3x10^-8^ | 1.29 | 0.03 | 0.07 | 0.05 | 0.01 | 0.37 | 0.35 | 0.15 | 0.27 |
| rs10998283^10^ | *TET1* | 10:68569307 | A | - | - | 1.15 | 3.9x10^-8^ | 1.11 | 0.45 | 0.04 | 0.04 | 0.01 | 0.12 | 0.16 | 0.15 | 0.27 |
| **rs12779301^9^** | *LHPP* | 10:124604086 | C | 1.22 | 5.7x10^-13^ | - | - | 1.20 | 2.4x10^-3^ | 0.70 | 0.66 | 0.64 | 0.53 | 0.60 | 0.66 | 0.59 |
| rs35837782^18^ | *LHPP* | 10:124604740 | G | - | - | 1.12 | 3.6x10^-4^ | 1.23 | 4.0x10^-4^ | 0.70 | 0.65 | 0.64 | 0.53 | 0.60 | 0.64 | 0.57 |
| **rs4762284^18^** | *ELK3* | 12:96218984 | T | 1.15 | 3.8x10^-7^ | 1.10 | 1.2x10^-3^ | 1.03 | 0.58 | 0.46 | 0.45 | 0.45 | 0.49 | 0.60 | 0.30 | 0.31 |
| **rs2239630^9^** | *CEBPE* | 14:23120140 | A | 1.28 | 1.7x10^-21^ | - | - | - | - | - | - | 0.94 | 0.52 | 0.56 | 0.45 | 0.58 |
| rs2239633^11^ | *CEBPE* | 14:23119848 | A | - | - | 0.81 | 1.2x10^-13^ | - | - | - | - | 0.17 | 0.45 | 0.35 | 0.48 | 0.39 |
| rs2290400^11^ | *1KZF3* | 17:39909987 | T | - | - | 1.14 | 3.2x10^-6^ | 1.04 | 0.44 | 0.54 | 0.53 | 0.51 | 0.60 | 0.71 | 0.49 | 0.62 |
| **rs10853104^9^** | *IGF2BP1* | 17:49014714 | T | 1.33 | 1.8x10^-8^ | 1.06 | 0.054 | 1.05 | 0.42 | 0.64 | 0.64 | 0.69 | 0.42 | 0.12 | 0.47 | 0.40 |
| **rs9976326^9^** | *ERG* | 21:38404563 | T | 1.33 | 4.8x10^-9^ | - | - | 1.21 | 0.02 | 0.14 | 0.11 | 0.06 | 0.30 | 0.19 | 0.25 | 0.41 |
| rs8131436^19^ | *ERG* | 21:38417684 | C | - | - | 1.12 | 1.4x10^-4^ | 1.14 | 0.04 | 0.23 | 0.21 | 0.19 | 0.38 | 0.27 | 0.32 | 0.43 |

Abbreviations: CHR, chromosome; BP, base position, GRCh38 (hg38) build; EA, effect (risk) allele; EAF, effect allele frequency; OR, odds ratio; CI, confidence interval; P, p-value. Odds ratios (ORs) and 95% confidence intervals (CIs) are from logistic regression models. All presented p-values are from two-sided statistical tests and are not adjusted for multiple testing.

1. References are annotated in superscript. Bolded SNPs are those identified in Vijayakrishnan et al. (2019) and included in the European ancestry B-ALL PRS.
2. EA is T in Vijayakrishnan et al.
3. EA is A in Vijayakrishnan et al.
4. EA is C in Vijayakrishnan et al.

**Supplementary Table 5**: Ancestry-matched B-ALL polygenic risk score (PRS) associations in African American children in ADMIRAL

|  | Sample | PRS quantiles | OR (95% CI) | P |
| --- | --- | --- | --- | --- |
| ADMIRAL meta-analysis PRS | Combined (N=4,200) | <Median risk | Ref. |  |
| (index variants listed in Table 1) |  | 50 to 59% | 2.18 (1.72-2.76) | 9.4x10^-11^ |
|  |  | 60 to 69% | 1.94 (1.38-2.73) | 1.4x10^-4^ |
|  |  | 70 to 79% | 1.85 (1.39-2.48) | 3.1x10^-5^ |
|  |  | 80 to 89% | 4.43 (3.47-5.65) | 6.0x10^-33^ |
|  |  | Top decile | 7.87 (6.22-9.97) | 6.9x10^-66^ |
| ADMIRAL Discovery PRS (P<5x10^-6^) | Replication  N=920 | <Median risk | Ref. |  |
| (variants at known loci *ARID5B, IKZF1* omitted; |  | 50 to 59% | 0.81 (0.43-1.54) | 0.52 |
| index variants in Supplementary Table 3) |  | 60 to 69% | 1.10 (0.62-1.95) | 0.74 |
|  |  | 70 to 79% | 1.53 (0.91-2.59) | 0.11 |
|  |  | 80 to 89% | 2.39 (1.41-4.04) | 1.2x10^-3^ |
|  |  | Top decile | 2.84 (1.68-4.82) | 1.1x10^-4^ |
| EUR PRS (Vijayakrishnan et al.) | Replication  N=920 | <Median risk | Ref. |  |
| (variants at known loci *ARID5B, IKZF1* omitted; |  | 50 to 59% | 1.41 (0.84-2.36) | 0.20 |
| QCed index variants in Supplementary Table 4) |  | 60 to 69% | 0.73 (0.40-1.32) | 0.29 |
|  |  | 70 to 79% | 1.20 (0.70-2.06) | 0.50 |
|  |  | 80 to 89% | 1.27 (0.71-2.25) | 0.42 |
|  |  | Top decile | 1.56 (0.86-2.81) | 0.14 |

Odds ratios (ORs) and 95% confidence intervals (CIs) are from logistic regression models. All presented p-values are from two-sided statistical tests and are not adjusted for multiple testing.

**Supplementary Table 6**: Associations between lead ALL risk variants and global AFR ancestry proportion in the combined sample, cases, and controls

|  |  | Combined (N=4,200) | | Cases (N=840) | | Controls (N=3,360) | |
| --- | --- | --- | --- | --- | --- | --- | --- |
| rsid | EA | Beta | P | Beta | P | Beta | P |
| rs17133807 | A | -0.21 | 3.8x10^-6^ | -0.29 | 9.0x10^-3^ | -0.19 | 1.2x10^-4^ |
| rs7090445 | C | -0.44 | 3.7x10^-21^ | -0.48 | 2.2x10^-5^ | -0.43 | 1.8x10^-17^ |
| rs77632976 | T | 0.07 | 3.6x10^-3^ | 0.05 | 0.42 | 0.08 | 2.8x10^-3^ |
| rs112113758 | T | 0.15 | 4.4x10^-9^ | 0.22 | 7.5x10^-4^ | 0.13 | 1.1x10^-6^ |
| rs183221417 | C | 0.11 | 1.7x10^-8^ | 0.29 | 3.2x10^-7^ | 0.06 | 1.5x10^-3^ |
| rs867166159 | T | 0.07 | 2.0x10^-6^ | 0.15 | 1.8x10^-3^ | 0.06 | 2.8x10^-4^ |
| rs76135126 | C | 0.07 | 1.8x10^-3^ | 0.11 | 0.077 | 0.06 | 9.7x10^-3^ |
| rs113299167 | T | 0.14 | 1.0x10^-10^ | 0.28 | 2.3x10^-6^ | 0.11 | 3.8x10^-6^ |
| rs116677565 | C | 0.05 | 5.3x10^-3^ | 0.06 | 0.24 | 0.04 | 8.0x10^-3^ |

Abbreviations: rsid, variant identifier using dbSNP build 151; EA, effect allele. Betas are from linear regression models and all presented p-values are from two-sided statistical tests and are not adjusted for multiple testing. The Bonferroni-corrected p-value threshold is 0.05/9 = 5.6x10^-3^.

**Supplementary Table 7**: Local ancestry haplotype associations corresponding to top B-ALL risk variants in the combined ADMIRAL data (N=4,200, 840 cases)

| Locus | CHR | Start BP | End BP | Local ancestry tract size (kb) | # of variants | OR (95% CI) | P |
| --- | --- | --- | --- | --- | --- | --- | --- |
| *FAM98A* | 2 | 34664547 | 34905779 | 241.2 | 1418 | 0.87 (0.76-1.00) | 0.057 |
| *CNTN4* | 3 | 2290394 | 2293378 | 3.0 | 11 | 1.04 (0.91-1.20) | 0.56 |
| *FAM174A* | 5 | 99924626 | 100606241 | 681.6 | 3272 | 1.03 (0.89-1.19) | 0.69 |
| *IKZF1* | 7 | 50351864 | 50628551 | 276.7 | 1697 | 0.87 (0.76-1.00) | 0.051 |
| *ZNG1A* | 9 | 41114 | 401299 | 360.2 | 1749 | 1.01 (0.87-1.17) | 0.89 |
| *ARID5B* | 10 | 61923202 | 62019923 | 96.7 | 306 | 0.99 (0.86-1.15) | 0.94 |
| *SLC37A4* | 11 | 119027526 | 119344061 | 316.5 | 928 | 0.92 (0.80-1.07) | 0.26 |
| *TMEM132D* | 12 | 129930958 | 130010662 | 79.7 | 434 | 1.07 (0.93-1.24) | 0.34 |
| *NDN* | 15 | 24076578 | 24735636 | 659.1 | 5049 | 0.96 (0.84-1.11) | 0.60 |

Abbreviations: CHR, chromosome; BP, base position, GRCh38 (hg38) build; OR, odds ratio; CI, confidence interval; P, p-value. Odds ratios (ORs) and 95% confidence intervals (CIs) are from logistic regression models. All presented p-values are from two-sided statistical tests and are not adjusted for multiple testing. The Bonferroni-corrected p-value threshold is 0.05/9 = 5.6x10^-3^.

**Supplementary Table 8**: Functional annotation of variants in 99% credible sets for novel B-ALL loci

|  |  |  |  |  |  |  | **RegulomeDB** | | | | | | | | | **Whole blood *cis*-eQTLs (Kachuri et al.)** | |
| --- | --- | --- | --- | --- | --- | --- | --- | --- | --- | --- | --- | --- | --- | --- | --- | --- | --- |
| Set | Chr:BP | rsid^a^ | Mean Rsq | Cumulative probability | Location | Closest Ensembl gene  (distance in bp) | Overall functional probability | Rank | Blood functional probability^b^ | Blood  p-value^b^ | Bone marrow functional probability^c^ | Bone marrow  p-value^c^ | TF motifs | Chromatin accessibility peak  (cell line) | ChIP-seq | Admixed ancestry (AA, PR, MX, LA) | AA only |
| 1 | 2:34823085 | rs79191888 | 0.966 | 0.996 | ncRNA intronic | *AC012593.1* | 0.18 | 7 | 0.05 | 1.00 | 0.08 | 1.00 |  |  |  |  |  |
| 1 | 2:34828430 | **rs77632976** | 0.964 | 0.907 | ncRNA intronic | *AC012593.1* | 0.18 | 7 | 0.05 | 1.00 | 0.05 | 1.00 |  |  |  |  |  |
| 2 | 3:2291928 | rs140581766 | 0.898 | 0.995 | intronic | *CNTN4* | 0.13 | 5 | 0.04 | 1.00 | 0.04 | 1.00 |  |  |  |  |  |
| 2 | 3:2292232 | **rs112113758** | 0.904 | 0.734 | intronic | *CNTN4* | 0.18 | 7 | 0.05 | 1.00 | 0.05 | 1.00 |  |  |  |  |  |
| 2 | 3:2293659 | rs113085788 | 0.909 | 0.882 | intronic | *CNTN4* | 0.40 | 7 | 0.12 | 1.00 | 0.12 | 1.00 |  |  |  |  |  |
| 2 | 3:2294705 | rs112398377 | 0.908 | 0.387 | intronic | *CNTN4* | 0.18 | 7 | 0.05 | 1.00 | 0.05 | 1.00 |  |  |  |  |  |
| 2 | 3:2297462 | rs76518082 | 0.925 | 0.989 | intronic | *CNTN4* | 0.31 | 6 | 0.09 | 1.00 | 0.09 | 1.00 |  |  |  |  |  |
| 2 | 3:2297952 | rs114333116 | 0.925 | 0.965 | intronic | *CNTN4* | 0.59 | 5 | 0.17 | 0.68 | 0.17 | 0.70 |  |  |  |  |  |
| 2 | 3:2297987 | rs116322842 | 0.925 | 0.952 | intronic | *CNTN4* | 0.81 | 2b | 0.42 | 9.9E-116 | 0.24 | 5.4E-09 | ZNF135, ZNF460 | NAMALWA | CEBPA in BLaER1 |  |  |
| 2 | 3:2298175 | rs74526449 | 0.925 | 0.978 | intronic | *CNTN4* | 0.18 | 7 | 0.05 | 1.00 | 0.05 | 1.00 |  |  |  |  |  |
| 2 | 3:2298834 | rs116156867 | 0.926 | 0.940 | intronic | *CNTN4* | 0.18 | 7 | 0.05 | 1.00 | 0.05 | 1.00 |  |  |  |  |  |
| 3 | 4:182886589 | rs7692433 | 0.959 | 0.989 | intergenic | *RP11-188P17.2; DCTD* (4386; 3502) | 0.74 | 4 | 0.20 | 0.047 | 0.20 | 0.054 | CEBPA, DDIT3 |  | CEBPA in BLaER1 |  |  |
| 3 | 4:182890782 | rs7662393 | 0.961 | 0.989 | UTR3 | *DCTD* | 0.61 | 4 | 0.16 | 0.96 | 0.16 | 0.97 |  | HG03378 |  |  |  |
| 3 | 4:183022416 | rs28581406 | 0.961 | 0.987 | ncRNA intronic | *RP11-335L23.6* | 0.61 | 4 | 0.18 | 0.46 | 0.18 | 0.49 |  |  | CEBPA in BLaER1 |  |  |
| 3 | 4:183033353 | NA | 0.983 | 0.990 | ncRNA intronic | *RP11-335L23.6* | 0.18 | 7 | 0.05 | 1.00 | 0.05 | 1.00 |  |  |  |  |  |
| 3 | 4:183074252 | rs77871957 | 0.995 | 0.984 | intergenic | *RP11-335L23.6; RP11-335L23.4* (38152; 1228) | 0.69 | 3a | 0.20 | 7.9E-03 | 0.31 | 2.4E-36 |  |  | TAF9B in K562, CTCF in activated B cell |  |  |
| 3 | 4:183088406 | rs13136638 | 0.956 | 0.990 | intergenic | *RP11-335L23.4; RP11-335L23.5* (12373; 6017) | 0.59 | 5 | 0.17 | 0.68 | 0.17 | 0.70 |  |  |  |  |  |
| 3 | 4:183109811 | rs75085961 | 0.989 | 0.984 | intronic | *WWC2* | 0.61 | 4 | 0.18 | 0.46 | 0.18 | 0.49 |  |  |  |  |  |
| 3 | 4:183131644 | rs74328829 | 1.000 | 0.987 | intronic | *WWC2* | 0.13 | 5 | 0.04 | 1.00 | 0.06 | 1.00 |  |  |  |  |  |
| 3 | 4:183153203 | rs58271130 | 0.993 | 0.987 | intronic | *WWC2* | 0.70 | 4 | 0.21 | 2.5E-03 | 0.21 | 3.0E-03 | MXI1 | GM23338 |  |  |  |
| 3 | 4:183181330 | rs28479105 | 0.968 | 0.990 | intronic | *WWC2* | 0.61 | 4 | 0.18 | 0.46 | 0.18 | 0.49 |  |  |  |  |  |
| 3 | 4:183264286 | rs78720762 | 0.999 | 0.981 | intronic | *WWC2* | 0.61 | 4 | 0.20 | 0.056 | 0.18 | 0.49 |  |  |  |  |  |
| 3 | 4:183321375 | rs61272231 | 0.989 | 0.989 | downstream | *CLDN24; WWC2* (389) | 0.25 | 1d | 0.12 | 1.00 | 0.11 | 1.00 |  |  | CEBPA in BLaER1 | *CLDN22* |  |
| 3 | 4:183327946 | rs10027741 | 1 | 0.987 | intergenic | *CLDN24; CDKN2AIP* (5520; 116689) | 0.61 | 4 | 0.20 | 0.056 | 0.18 | 0.49 |  |  |  | *CLDN22* |  |
| 3 | 4:183328337 | rs2048472 | 1 | 0.990 | intergenic | *CLDN24; CDKN2AIP* (5911; 116298) | 0.51 | 7 | 0.15 | 1.00 | 0.15 | 1.00 |  |  |  | *CLDN22* | *CDKN2AIP* |
| 3 | 4:183330444 | rs10025123 | 0.996 | 0.967 | intergenic | *CLDN24; CDKN2AIP* (8018; 114191) | 0.13 | 5 | 0.04 | 1.00 | 0.06 | 1.00 |  |  |  | *CLDN22* |  |
| 3 | 4:183335952 | rs115880086 | 0.993 | 0.986 | intergenic | *CLDN24; CDKN2AIP* (13526; 108683) | 0.61 | 4 | 0.17 | 0.87 | 0.28 | 1.0E-23 |  | GM19043, GM18907 | ZNF175, ZNF584, FOSL1, ZNF589, ZNF354B, EP400 in K562 |  |  |
| 3 | 4:183336099 | rs28408355 | 0.996 | 0.899 | intergenic | *CLDN24; CDKN2AIP* (13673; 108536) | 0.71 | 5 | 0.21 | 2.0E-03 | 0.33 | 3.7E-47 | MEIS2, PBX2 |  | ZNF589, EP400 in K562 | *CLDN22* |  |
| 3 | 4:183338461 | rs4616804 | 1 | 0.976 | intergenic | *CLDN24; CDKN2AIP* (16035; 106174) | 0.13 | 5 | 0.04 | 1.00 | 0.06 | 1.00 |  |  |  | *CLDN22* |  |
| 3 | 4:183340371 | rs28790294 | 0.990 | 0.981 | intergenic | *CLDN24; CDKN2AIP* (17945; 104264) | 0.13 | 5 | 0.04 | 1.00 | 0.04 | 1.00 |  |  |  | *CLDN22* |  |
| 3 | 4:183341018 | rs28669318 | 0.993 | 0.973 | intergenic | *CLDN24; CDKN2AIP* (18592; 103617) | 0.13 | 5 | 0.04 | 1.00 | 0.04 | 1.00 |  |  |  | *CLDN22* |  |
| 3 | 4:183344391 | rs28417567 | 0.993 | 0.916 | intergenic | *CLDN24; CDKN2AIP* (21965; 100244) | 0.61 | 4 | 0.20 | 0.056 | 0.27 | 1.1E-19 |  |  | ZNF121 in K562 | *CLDN22* |  |
| 3 | 4:183344659 | rs112184916 | 0.987 | 0.979 | intergenic | *CLDN24; CDKN2AIP* (22233; 99976) | 0.61 | 4 | 0.20 | 0.056 | 0.35 | 1.9E-63 |  | HG03025 | CEBPA in BLaER1, ZNF121 in K562 |  |  |
| 3 | 4:183345820 | rs28377170 | 0.987 | 0.989 | intergenic | *CLDN24; CDKN2AIP* (23394; 98815) | 0.61 | 4 | 0.20 | 0.056 | 0.27 | 1.1E-19 |  |  | MGA in K562 |  |  |
| 3 | 4:183346205 | rs28407552 | 0.984 | 0.947 | intergenic | *CLDN24; CDKN2AIP* (23779; 98430) | 0.18 | 7 | 0.05 | 1.00 | 0.06 | 1.00 |  |  |  | *CLDN22* |  |
| 3 | 4:183347238 | rs112282213 | 0.993 | 0.961 | intergenic | *CLDN24; CDKN2AIP* (24812; 97397) | 0.36 | 5 | 0.12 | 1.00 | 0.16 | 0.96 |  |  |  |  |  |
| 3 | 4:183347814 | **rs28568357** | 0.991 | 0.748 | intergenic | *CLDN24; CDKN2AIP* (25388; 96821) | 0.61 | 4 | 0.17 | 0.91 | 0.27 | 1.1E-19 |  |  | ATF3 in K562, CEBPA in BLaER1 | *CLDN22* |  |
| 3 | 4:183348093 | rs10001023 | 0.985 | 0.988 | intergenic | *CLDN24; CDKN2AIP* (25667; 96542) | 0.73 | 3a | 0.17 | 0.85 | 0.34 | 1.9E-53 | RARG |  | CEBPA in BLaER1 |  |  |
| 3 | 4:183348123 | rs28573126 | 0.987 | 0.984 | intergenic | *CLDN24; CDKN2AIP* (25697; 96512) | 0.61 | 4 | 0.14 | 1.00 | 0.28 | 1.0E-23 |  |  | CEBPA in BLaER1 |  |  |
| 3 | 4:183348140 | rs28468468 | 0.980 | 0.881 | intergenic | *CLDN24; CDKN2AIP* (25714; 96495) | 0.67 | 2b | 0.25 | 1.3E-12 | 0.41 | 1.7E-112 | PLAGL1 |  | CEBPA in BLaER1 |  |  |
| 3 | 4:183348337 | rs78472980 | 0.986 | 0.978 | intergenic | *CLDN24; CDKN2AIP* (25911; 96298) | 0.76 | 2b | 0.29 | 4.2E-25 | 0.43 | 1.7E-125 | NRF1 | CD34+ cells | CEBPA in BLaER1 | *CLDN22* |  |
| 3 | 4:183348391 | rs74340863 | 0.986 | 0.986 | intergenic | *CLDN24; CDKN2AIP* (25965; 96244) | 0.76 | 2b | 0.29 | 6.4E-25 | 0.43 | 7.0E-125 | POU5F1/  SOX2 |  | CEBPA in BLaER1 |  |  |
| 3 | 4:183348446 | rs75590319 | 0.988 | 0.985 | intergenic | *CLDN24; CDKN2AIP* (26020; 96189) | 0.61 | 4 | 0.14 | 1.00 | 0.35 | 1.9E-63 |  |  | CEBPA in BLaER1, GTF2E2, ZFX in K562 |  |  |
| 3 | 4:183348698 | rs115644345 | 0.996 | 0.804 | intergenic | *CLDN24; CDKN2AIP* (26272; 95937) | 0.74 | 2b | 0.28 | 1.62E-21 | 0.43 | 1.15E-127 | SIX2 |  | 30+ Targets in K562 | *CLDN22* |  |
| 3 | 4:183349716 | rs76060227 | 1.000 | 0.933 | intergenic | *CLDN24; CDKN2AIP* (27290; 94919) | 0.61 | 4 | 0.35 | 1.25E-63 | 0.28 | 1.01E-23 |  | K562 | 22 Targets K562 | *CLDN22* |  |
| 3 | 4:183350178 | rs28461999 | 0.995 | 0.855 | intergenic | *CLDN24; CDKN2AIP* (27752; 94457) | 0.76 | 2b | 0.23 | 5.13E-06 | 0.23 | 6.69E-06 | ZNF135, ZNF460 |  | TFDP1, ZBTB33 in K562 |  |  |
| 3 | 4:183350474 | rs13122009 | 0.985 | 0.988 | intergenic | *CLDN24; CDKN2AIP* (28048; 94161) | 0.61 | 4 | 0.20 | 0.056 | 0.27 | 1.12E-19 |  |  | NFRKB in K562 |  |  |
| 3 | 4:183351154 | rs10010816 | 0.980 | 0.989 | intergenic | *CLDN24; CDKN2AIP* (28728; 93481) | 0.13 | 5 | 0.04 | 1.00 | 0.06 | 1.00 |  |  |  |  |  |
| 3 | 4:183353431 | rs57427622 | 0.978 | 0.955 | intergenic | *CLDN24; CDKN2AIP* (31005; 91204) | 0.48 | 2b | 0.14 | 1.00 | 0.14 | 1.00 |  |  |  |  |  |
| 3 | 4:183355524 | rs28654914 | 0.999 | 0.986 | intergenic | *CLDN24; CDKN2AIP* (33098; 89111) | 0.61 | 4 | 0.18 | 0.46 | 0.18 | 0.49 |  |  | PKNOX1, FOSL1, PBX2, MEIS2 in K562 |  |  |
| 3 | 4:183356978 | rs17074636 | 0.997 | 0.983 | intergenic | *CLDN24; CDKN2AIP* (34552; 87657) | 0.61 | 4 | 0.18 | 0.46 | 0.18 | 0.49 |  |  |  |  |  |
| 3 | 4:183388806 | rs6817330 | 0.965 | 0.988 | intergenic | *CLDN24; CDKN2AIP* (66380; 55829) | 0.61 | 4 | 0.20 | 0.056 | 0.20 | 0.064 |  |  |  |  |  |
| 3 | 4:183400588 | rs148653873 | 0.994 | 0.985 | intergenic | *CLDN24; CDKN2AIP* (78162; 44047) | 0.61 | 4 | 0.17 | 0.87 | 0.27 | 1.1E-19 |  |  | PRDM10 in K562 |  |  |
| 3 | 4:183400815 | NA | 0.979 | 0.988 | intergenic | *CLDN24; CDKN2AIP* (78389; 43820) | 0.70 | 4 | 0.23 | 2.8E-06 | 0.21 | 3.0E-03 | NR1H3, RXRA |  | EGR1, PRDM10 in K562 |  |  |
| 3 | 4:183401576 | NA | 0.991 | 0.983 | intergenic | *CLDN24; CDKN2AIP* (79150; 43059) | 0.61 | 4 | 0.20 | 0.056 | 0.18 | 0.49 |  |  | RBM25 in K562 |  |  |
| 3 | 4:183403906 | NA | 0.974 | 0.986 | intergenic | *CLDN24; CDKN2AIP* (81480; 40729) | 0.13 | 5 | 0.04 | 1.00 | 0.06 | 1.00 |  |  |  |  |  |
| 3 | 4:183405155 | NA | 0.970 | 0.988 | intergenic | *CLDN24; CDKN2AIP* (82729; 39480) | 0.24 | 5 | 0.07 | 1.00 | 0.07 | 1.00 |  |  |  |  |  |
| 3 | 4:183412975 | NA | 0.985 | 0.982 | intergenic | *CLDN24; CDKN2AIP* (90549; 31660) | 0.61 | 4 | 0.27 | 7.4E-20 | 0.27 | 1.1E-19 |  |  | MEIS2 in K562 |  |  |
| 4 | 5:100095464 | rs7731907 | 0.886 | 0.947 | intergenic | *Y RNA; RNU6-1119P* (73558; 58208) | 0.18 | 7 | 0.05 | 1.00 | 0.05 | 1.00 |  |  |  |  |  |
| 4 | 5:100102043 | **rs183221417** | 0.898 | 0.843 | intergenic | *Y RNA; RNU6-1119P* (80137; 51629) | 0.13 | 5 | 0.04 | 1.00 | 0.04 | 1.00 |  |  |  |  |  |
| 4 | 5:100109217 | rs61526151 | 0.885 | 0.993 | intergenic | *Y RNA; RNU6-1119P* (87311; 44455) | 0.61 | 4 | 0.18 | 0.46 | 0.16 | 0.97 |  |  | CEBPA in BLaER1 |  |  |
| 4 | 5:100127437 | rs76291187 | 0.933 | 0.971 | intergenic | *Y RNA; RNU6-1119P* (105531; 26235) | 0.18 | 7 | 0.05 | 1.00 | 0.05 | 1.00 |  |  |  |  |  |
| 5 | 5:111791133 | **rs115636216** | 0.956 | 0.996 | intronic | *NREP* | 0.00 | 6 | 0.00 | 1.00 | 0.00 | 1.00 |  |  |  | *EPB41L4A* |  |
| 6 | 9:191139 | **rs867166159** | 0.845 | 0.996 | intergenic | *ZNG1A; RP11-174M15.1* (11992; 5926) | 0.18 | 7 | 0.05 | 1.00 | 0.05 | 1.00 |  |  |  | *KANK1* |  |
| 7 | 11:54651552 | **rs112269413** | 0.903 | 0.741 | intergenic | *OR4A8; OR4A5* (9070; 13941) | 0.18 | 7 | 0.05 | 1.00 | 0.05 | 1.00 |  |  |  |  |  |
| 7 | 11:54671408 | rs1846272 | 0.922 | 0.383 | intergenic | *OR4A8; OR4A5* (9071; 13940) | 0.18 | 7 | 0.05 | 1.00 | 0.05 | 1.00 |  |  |  |  |  |
| 7 | 11:54673992 | rs111648967 | 0.917 | 0.479 | intergenic | *OR4C46; OR4A8* (67410; 11469) | 0.18 | 7 | 0.05 | 1.00 | 0.05 | 1.00 |  |  |  |  |  |
| 7 | 11:54679716 | rs1826995 | 0.922 | 0.656 | intergenic | *OR4C46; OR4A8* (69994; 8885) | 0.18 | 7 | 0.05 | 0.013 | 0.05 | 0.016 |  |  |  |  |  |
| 7 | 11:54684131 | rs188411320 | 0.912 | 0.986 | downstream | *OR4A8; OR4A5* (8235; 14776) | 0.18 | 7 | 0.05 | 1.00 | 0.05 | 1.00 |  |  |  |  |  |
| 7 | 11:54688345 | rs7936091 | 0.924 | 0.890 | intergenic | *OR4C46; OR4A8* (75718; 3161) | 0.18 | 7 | 0.05 | 1.00 | 0.05 | 1.00 |  |  |  |  |  |
| 7 | 11:54689226 | rs114133382 | 0.924 | 0.943 | intergenic | *OR4C46; OR4A8* (47554; 31325) | 0.68 | 6 | 0.20 | 1.00 | 0.20 | 1.00 | JUND |  |  |  |  |
| 7 | 11:54690419 | rs7949373 | 0.923 | 0.982 | intergenic | *OR4A8; OR4A5* (8980; 14031) | 0.18 | 7 | 0.05 | 1.00 | 0.05 | 1.00 |  |  |  |  |  |
| 7 | 11:54692056 | rs7949758 | 0.924 | 0.570 | intergenic | *OR4A8; OR4A5* (4524; 18487) | 0.18 | 7 | 0.05 | 1.00 | 0.05 | 1.00 |  |  |  |  |  |
| 7 | 11:54692801 | rs147033569 | 0.923 | 0.819 | intergenic | *OR4A8; OR4A5* (5405; 17606) | 0.18 | 7 | 0.05 | 0.68 | 0.05 | 0.70 |  |  |  |  |  |
| 7 | 11:54692891 | rs190558167 | 0.924 | 0.138 | intergenic | *OR4A8; OR4A5* (6598; 16413) | 0.18 | 7 | 0.05 | 1.00 | 0.05 | 1.00 |  |  |  |  |  |
| 7 | 11:54692892 | rs116297461 | 0.924 | 0.276 | intergenic | *OR4A8* (310) | 0.18 | 7 | 0.05 | 1.00 | 0.05 | 1.00 |  |  |  |  |  |
| 7 | 11:54716855 | rs146946515 | 0.923 | 0.990 | intergenic | *OR4A5; TRIM48* (8953; 545300) | 0.59 | 5 | 0.17 | 6.6E-24 | 0.17 | 1.0E-23 |  |  |  |  |  |
| 8 | 11:119033074 | **rs76135126** | 0.885 | 0.974 | intergenic | *SLC37A4; RP11-110I1.6* (2168; 11114) | 0.51 | 2b | 0.15 | 1.00 | 0.15 | 1.00 |  |  |  | *SLC37A4, VPS11* | *VPS11* |
| 8 | 11:119037428 | rs187532742 | 0.926 | 0.986 | intergenic | *SLC37A4; RP11-110I1.6* (6522; 6760) | 0.13 | 5 | 0.04 | 1.00 | 0.04 | 1.00 |  |  |  |  |  |
| 8 | 11:119042468 | rs77765196 | 0.924 | 0.993 | intergenic | *SLC37A4; RP11-110I1.6* (11562; 1720) | 0.61 | 4 | 0.28 | 1.00 | 0.28 | 1.00 |  | GM12878 | ZEB2 in K562 | *SLC37A4* |  |
| 9 | 12:129960984 | rs113399637 | 0.948 | 0.998 | intergenic | *TMEM132D; RP11-474D1.4* (57816; 62652) | 0.18 | 7 | 0.05 | 1.00 | 0.05 | 1.00 |  |  |  |  |  |
| 9 | 12:129961841 | **rs113299167** | 0.948 | 0.566 | intergenic | *TMEM132D; RP11-474D1.4* (56959; 63509) | 0.61 | 4 | 0.18 | 0.46 | 0.18 | 0.49 |  |  | ZNF512 in K562 |  |  |
| 10 | 15:24174608 | **rs116677565** | 0.899 | 0.762 | ncRNA intronic | *PWRN1* | 0.00 | 6 | 0.00 | 1.00 | 0.00 | 1.00 |  |  |  |  |  |
| 10 | 15:24178668 | rs5002270 | 0.899 | 0.381 | ncRNA intronic | *PWRN1* | 0.13 | 5 | 0.04 | 1.00 | 0.04 | 1.00 |  |  |  |  |  |
| 10 | 15:24178703 | rs145683427 | 0.898 | 0.972 | ncRNA intronic | *PWRN1* | 0.13 | 5 | 0.04 | 1.00 | 0.04 | 1.00 |  |  |  |  |  |
| 10 | 15:24188023 | rs114311981 | 0.893 | 0.990 | ncRNA intronic | *PWRN1* | 0.18 | 7 | 0.05 | 1.00 | 0.05 | 1.00 |  |  |  |  |  |
| 10 | 15:24188183 | rs12101485 | 0.894 | 0.992 | ncRNA intronic | *PWRN1* | 0.01 | 6 | 0.00 | 1.00 | 0.00 | 1.00 |  |  |  |  |  |
| 10 | 15:24188291 | rs28498194 | 0.893 | 0.987 | ncRNA intronic | *PWRN1* | 0.13 | 5 | 0.04 | 1.00 | 0.04 | 1.00 |  |  |  |  |  |
| 10 | 15:24188999 | rs28550112 | 0.905 | 0.979 | ncRNA intronic | *PWRN1* | 0.18 | 7 | 0.05 | 1.00 | 0.05 | 1.00 |  |  |  |  |  |
| 10 | 15:24193959 | rs147499604 | 0.898 | 0.984 | ncRNA intronic | *PWRN1* | 0.81 | 2b | 0.24 | 2.8E-09 | 0.47 | 4.7E-174 |  |  | CEBPA in BLaER1 |  |  |

1. Bolded rsids reflect index variants from discovery analyses.
2. RegulomeDB blood and bone marrow functional probabilities ranging from 0 to 1 are provided, based on RegulomeDB-based definitions of blood- and bone marrow-specific cell types. One-sided p-values indicating how extreme the blood and bone marrow functional probabilities are for each credible set variant were calculated (i.e., “blood p-value” and “bone marrow p-value”) based on Z-scores from null distributions of corresponding blood and bone marrow RegulomeDB functional probability scores for 4,586 SNPs associated with any carcinoma (non-hematological cancer) reported in the NHGRI-EBI GWAS Catalog. Presented p-values are not corrected for multiple testing. The Bonferroni-corrected P-value threshold is 0.05/90=5.6x10^-4^.

Abbreviations: rsid, genetic variant identifier using dbSNP build 151; Chr, chromosome; BP, base position, GRCh38 (hg38) build; AA, African American; PR, Puerto Rican; MX, Mexican American; LA, Latino American.

**Supplementary Table 9**: Colocalizations with posterior probabilities >0.01 for credible set SNVs

| Index variant (chr:BP) | Kachuri et al. eQTL Dataset | Gene | Posterior probability of colocalization | Best credible set SNP in region | Best credible set SNP probability of colocalization | Index SNP probability of colocalization |
| --- | --- | --- | --- | --- | --- | --- |
| 2:34828430 | AA | No significant eQTLs |  |  |  |  |
|  | All admixed | No credible set variants |  |  |  |  |
| 3:2292232 | AA | ENSG00000271870 | 0.039 | 3:2292232 | 0.378 | 0.378 |
|  | All admixed | ENSG00000271870 | 0.054 | 3:2294705 | 0.391 | 0.332 |
|  | All admixed | CNTN4-AS1 | 0.072 | 3:2292232 | 0.379 | 0.379 |
|  | All admixed | CRBN | 0.049 | 3:2294705 | 0.388 | 0.342 |
|  | All admixed | TRNT1 | 0.027 | 3:2294705 | 0.387 | 0.343 |
| 4:183347814 | AA | CDKN2AIP | 0.030 | 4:183347814 | 0.345 | 0.345 |
|  | AA | RWDD4 | 0.029 | 4:183347814 | 0.273 | 0.273 |
|  | AA | TRAPPC11 | 0.026 | 4:183347814 | 0.386 | 0.386 |
|  | All admixed | ENSG00000272744 | 0.027 | 4:183347814 | 0.414 | 0.414 |
|  | All admixed | ING2 | 0.028 | 4:183347814 | 0.457 | 0.457 |
| 5:100102043 | AA | MTCO3P22 | 0.042 | 5:100102043 | 0.842 | 0.842 |
|  | AA | ST8SIA4 | 0.028 | 5:100102043 | 0.834 | 0.834 |
|  | AA | FAM174A | 0.020 | 5:100102043 | 0.842 | 0.842 |
|  | All | MTCO3P22 | 0.049 | 5:100102043 | 0.841 | 0.841 |
|  | All admixed | MTND4P35 | 0.064 | 5:100102043 | 0.852 | 0.852 |
| 5:111791133 | AA | RPS3AP21 | 0.048 | 5:111791133 | 0.961 | 0.961 |
|  | All admixed | WDR36 | 0.068 | 5:111791133 | 0.955 | 0.955 |
|  | All admixed | RPS3AP21 | 0.054 | 5:111791133 | 0.965 | 0.965 |
| 9:191139 | AA | DOCK8-AS1 | 0.161 | 9:191139 | 0.939 | 0.939 |
|  | AA | WASHC1 | 0.017 | 9:191139 | 0.220 | 0.220 |
|  | All admixed | RPL12P25 | 0.028 | 9:191139 | 0.585 | 0.585 |
| 11:54651552 | AA | No significant eQTLs |  |  |  |  |
|  | All admixed | No significant eQTLs |  |  |  |  |
| 11:119033074 | AA | CD3D | 0.124 | 11:119033074 | 0.621 | 0.621 |
|  | AA | SMIM35 | 0.074 | 11:119033074 | 0.698 | 0.698 |
|  | AA | MCAM | 0.068 | 11:119033074 | 0.874 | 0.874 |
|  | AA | KMT2A | 0.062 | 11:119033074 | 0.545 | 0.545 |
|  | AA | HYOU1 | 0.051 | 11:119033074 | 0.490 | 0.490 |
|  | AA | VPS11-DT | 0.048 | 11:119033074 | 0.800 | 0.800 |
|  | AA | JAML | 0.046 | 11:119033074 | 0.692 | 0.692 |
|  | AA | CCDC153 | 0.040 | 11:119033074 | 0.692 | 0.692 |
|  | AA | CXCR5 | 0.039 | 11:119033074 | 0.706 | 0.706 |
|  | AA | UPK2 | 0.038 | 11:119033074 | 0.674 | 0.674 |
|  | AA | DPAGT1 | 0.038 | 11:119033074 | 0.764 | 0.764 |
|  | AA | NECTIN1 | 0.037 | 11:119033074 | 0.725 | 0.725 |
|  | AA | DDX6 | 0.025 | 11:119033074 | 0.581 | 0.581 |
|  | AA | HSPE1P18 | 0.018 | 11:119033074 | 0.809 | 0.809 |
|  | AA | TMEM25 | 0.010 | 11:119033074 | 0.477 | 0.477 |
|  | All admixed | NECTIN1 | 0.036 | 11:119033074 | 0.615 | 0.615 |
|  | All admixed | UPK2 | 0.032 | 11:119033074 | 0.545 | 0.545 |
|  | All admixed | TTC36-AS1 | 0.052 | 11:119033074 | 0.738 | 0.738 |
|  | All admixed | MCAM | 0.026 | 11:119033074 | 0.683 | 0.683 |
|  | All admixed | SMIM35 | 0.072 | 11:119033074 | 0.808 | 0.808 |
|  | All admixed | PHLDB1 | 0.034 | 11:119033074 | 0.752 | 0.752 |
|  | All admixed | CD3G | 0.051 | 11:119033074 | 0.718 | 0.718 |
|  | All admixed | CCDC153 | 0.025 | 11:119033074 | 0.690 | 0.690 |
|  | All admixed | UBE4A | 0.073 | 11:119033074 | 0.879 | 0.879 |
|  | All admixed | SCN4B | 0.090 | 11:119033074 | 0.774 | 0.774 |
|  | All admixed | HSPE1P18 | 0.015 | 11:119033074 | 0.672 | 0.672 |
|  | All admixed | TMEM25 | 0.016 | 11:119033074 | 0.572 | 0.572 |
|  | All admixed | JAML | 0.025 | 11:119033074 | 0.612 | 0.612 |
| 12:129961841 | AA | RAN | 0.049 | 12:129961841 | 0.489 | 0.489 |
|  | AA | STX2 | 0.029 | 12:129961841 | 0.487 | 0.487 |
|  | AA | RIMBP2 | 0.016 | 12:129961841 | 0.266 | 0.266 |
|  | All admixed | ADGRD1 | 0.120 | 12:129961841 | 0.564 | 0.564 |
| 15:24174608 | AA | ENSG00000261069 | 0.047 | 15:24174608 | 0.304 | 0.304 |
|  | AA | NDN | 0.032 | 15:24178668 | 0.257 | 0.248 |
|  | All admixed | PWAR5 | 0.043 | 15:24178668 | 0.289 | 0.284 |
|  | All admixed | ENSG00000261069 | 0.052 | 15:24174608 | 0.307 | 0.307 |
|  | All admixed | MKRN3 | 0.029 | 15:24178668 | 0.209 | 0.209 |
|  | All admixed | SNURF | 0.044 | 15:24174608 | 0.260 | 0.260 |
|  | All admixed | SNRPN | 0.052 | 15:24174608 | 0.252 | 0.252 |

Abbreviations: AA, African American.

**Supplementary Table 10**: List of 3D chromatin looping datasets

| **Cell Type** | **Data Type** | **Number** | **Publication** |
| --- | --- | --- | --- |
| CD34+ human hematopoietic progenitor cell | Promoter Capture Hi-C | n=1 | Mifsud et al.^20^ ***Nature Genetics*** 2015 |
| GM12878 | Promoter Capture Hi-C | n=1 | Mifsud et al.^20^ ***Nature Genetics*** 2015 |
| B-cells (total) | Promoter Capture Hi-C | n=1 | Javierre et al.^21^ ***Cell*** 2016 |
| B-cells (naïve) | Promoter Capture Hi-C | n=1 | Javierre et al.^21^ ***Cell*** 2016 |
| Primary B-ALL cell | Promoter Capture Hi-C | n=10 | Barnett et al.^22^ ***Cell Genomics*** 2023 |
| B-ALL cell line (697, BALL1, Nalm6, REH, RS411, SEM, SUPB15) | Promoter Capture Hi-C | n=7 | Bhattarai et al.^23^ ***Nature Communications*** 2024 |
| GM12878, cohesin | HiChIP | n=1 | Mumbach et al.^24^ ***Nature Methods*** 2016 |
| 697, H3K27ac | HiChIP | n=1 | Bergeron et al.^25^ ***Leukemia*** 2022 |
| Nalm6, H3K27ac | HiChIP | n=1 | Bergeron et al.^25^ ***Leukemia*** 2022 |
| B-ALL cell line (697, BALL1, Nalm6, REH, RS411, SEM, SUPB15), H3K27ac | HiChIP | n=7 | Bhattarai et al.^23^ ***Nature Communications*** 2024 |
| Adult B-cell, RNA polymerase II | ChIA-PET | n=2 | ENCODE consortium ***Nature*** 2012^26^; ENCODE consortium ***Nature*** 2020^27^ |
| Adult B-cell, CTCF | ChIA-PET | n=2 | ENCODE consortium ***Nature*** 2012^26^; ENCODE consortium ***Nature*** 2020^27^ |
| GM12878, RNA polymerase II | ChIA-PET | n=2 | ENCODE consortium ***Nature*** 2012^26^; ENCODE consortium ***Nature*** 2020^27^ |
| GM12878, CTCF | ChIA-PET | n=2 | ENCODE consortium ***Nature*** 2012^26^; ENCODE consortium ***Nature*** 2020^27^ |

**Supplementary Table 11**: Candidate target genes for novel B-ALL risk variants using 3D chromatin looping data in hematopoietic cells

| rsid | Chr:BP | Potential target genes (cell type) | Method |
| --- | --- | --- | --- |
| rs77632976 | chr2:34828430 | N/A | N/A |
| rs112113758 | chr3:2292232 | N/A | N/A |
| rs28568357 | chr4:183347814 | *WWC2* (CD34+, GM12878)  *CDKN2AIP* (CD34+, GM12878)  *TENM3* (GM12878)  *DCTD* (GM12878)  *CLDN22* (CD34+, GM12878)  *CLDN24* (CD34+, GM12878)  *ING2* (GM12878)  *RWDD4* (GM12878)  *TRAPPC11* (GM12878)  *STOX2* (GM12878)  *ENPP6* (GM12878) | Promoter Capture Hi-C (Mifsud et al.^20^) |
| rs183221417 | chr5:100102043 | N/A | N/A |
| rs115636216 | chr5:111791133 | *EPB41L4A* (GM12878)  *NREP* (Nalm6) | Promoter Capture Hi-C (Mifsud et al.^20^)  H3K27ac HiChIP (Bergeron et al.^25^) |
| rs17133807 | chr7:50409989 | *IKZF1* (GM12878, B total, B naïve, primary B-ALL)  *FIGNL1* (CD34+, GM12878)  *SPATA48* (GM12878, B total, B naïve, primary B-ALL, 697, BALL1, Nalm6, REH, RS411; SUPB15)  *ZPBP* (GM12878, primary B-ALL, 697, BALL1, Nalm6, REH)  COBL (Nalm6) | Promoter Capture Hi-C (Mifsud et al.^20^; Javierre et al.^21^; Barnett et al.^22^; Bhattarai et al.^23^)  H3K27ac HiChIP (Bergeron et al.^25^) |
| rs867166159 | chr9:191139 | *DOCK8* (CD34+, GM12878) | Promoter Capture Hi-C (Mifsud et al.^20^) |
| rs7090445 | chr10:61961417 | *ARID5B* (CD34+, GM12878, 697, Nalm6)  *ADO* (GM12878) | Promoter Capture Hi-C (Mifsud et al.^20^); H3K27ac HiChIP (Bergeron et al.^25^) |
| rs112269413 | chr11:54651552 | N/A | N/A |
| rs76135126 | chr11:119033074 | *SLC37A4* promoter region | N/A |
| rs113299167 | chr12:129961841 | *TMEM132D* (CD34+, GM12878) | Promoter Capture Hi-C (Mifsud et al.^20^) |
| rs116677565 | chr15:24174608 | N/A | N/A |

Abbreviations: rsid, genetic variant identifier using dbSNP build 151; Chr, chromosome; BP, base position, GRCh38 (hg38) build.

**Supplementary Table 12**: Replication of B-ALL associations identified in African American children (ADMIRAL meta-analysis) in a Hispanic/Latino population in CCRLP (P<0.05)

|  |  |  |  |  |  | ADMIRAL, AFR | | CCRLP, Hispanic/Latino | | | | 1000 Genomes 30X | | | | |
| --- | --- | --- | --- | --- | --- | --- | --- | --- | --- | --- | --- | --- | --- | --- | --- | --- |
| rsid | CHR | BP | Index (CHR:BP) | Other allele | Tested allele | Meta-analysis OR (95% CI) | P | Tested AF | OR (95% CI) | P | AFR  r2 | | AFR  D’ | AMR  r2 | AMR  D’ |  |
| rs76291187 | 5 | 100127437 | 5:100102043 | C | T | 2.88 (2.18-3.8) | 1.0E-13 | 0.004 | 1.74 (1.54-2.80) | 0.021 | 0.58 | | 0.80 | 0.67 | 1 |  |
| rs55736272 | 7 | 50394709 | 7:50409989 | T | A | 1.56 (1.38-1.76) | 8.0E-13 | 0.27 | 1.29 (1.19-1.40) | 9.5E-10 | 0.78 | | 0.94 | 0.98 | 0.99 |  |
| rs62447205 | 7 | 50398132 | 7:50409989 | A | G | 1.56 (1.38-1.76) | 8.5E-13 | 0.27 | 1.29 (1.19-1.40) | 8.3E-10 | 0.78 | | 0.94 | 0.98 | 0.99 |  |
| rs11978267 | 7 | 50398606 | 7:50409989 | A | G | 1.56 (1.38-1.76) | 8.1E-13 | 0.27 | 1.29 (1.19-1.40) | 8.2E-10 | 0.78 | | 0.94 | 0.98 | 0.99 |  |
| rs6973210 | 7 | 50398997 | 7:50409989 | G | A | 1.55 (1.38-1.75) | 1.1E-12 | 0.27 | 1.29 (1.19-1.40) | 8.3E-10 | 0.78 | | 0.94 | 0.98 | 0.99 |  |
| rs6960400 | 7 | 50399099 | 7:50409989 | A | G | 1.55 (1.38-1.75) | 1.0E-12 | 0.27 | 1.29 (1.19-1.40) | 8.3E-10 | 0.78 | | 0.94 | 0.98 | 0.99 |  |
| rs10278451 | 7 | 50401254 | 7:50409989 | G | T | 1.54 (1.37-1.74) | 3.0E-12 | 0.27 | 1.29 (1.19-1.40) | 9.4E-10 | NA | | NA | NA | NA |  |
| rs11552047 | 7 | 50401853 | 7:50409989 | C | T | 1.54 (1.37-1.74) | 2.5E-12 | 0.27 | 1.29 (1.19-1.40) | 1.7E-09 | 0.80 | | 0.95 | 0.98 | 0.99 |  |
| rs11980379 | 7 | 50402283 | 7:50409989 | T | C | 1.55 (1.37-1.75) | 2.0E-12 | 0.27 | 1.29 (1.19-1.40) | 1.6E-09 | 0.80 | | 0.95 | 0.98 | 0.99 |  |
| rs4132601 | 7 | 50402906 | 7:50409989 | T | G | 1.55 (1.37-1.75) | 2.0E-12 | 0.27 | 1.29 (1.19-1.40) | 1.6E-09 | 0.80 | | 0.95 | 0.98 | 0.99 |  |
| rs11980407 | 7 | 50403915 | 7:50409989 | G | A | 1.55 (1.37-1.75) | 2.0E-12 | 0.27 | 1.29 (1.19-1.40) | 1.5E-09 | 0.80 | | 0.95 | 0.98 | 0.99 |  |
| rs62445866 | 7 | 50404626 | 7:50409989 | G | A | 1.55 (1.37-1.75) | 1.9E-12 | 0.27 | 1.29 (1.19-1.40) | 1.4E-09 | 0.80 | | 0.95 | 0.98 | 0.99 |  |
| rs58923657 | 7 | 50405144 | 7:50409989 | C | T | 1.55 (1.37-1.75) | 2.2E-12 | 0.27 | 1.29 (1.19-1.40) | 9.4E-10 | 0.80 | | 0.95 | 0.98 | 0.99 |  |
| rs6964969 | 7 | 50405553 | 7:50409989 | A | G | 1.55 (1.37-1.75) | 2.0E-12 | 0.27 | 1.29 (1.19-1.40) | 9.7E-10 | 0.80 | | 0.95 | 0.98 | 0.99 |  |
| rs6956014 | 7 | 50405592 | 7:50409989 | T | C | 1.55 (1.37-1.75) | 1.7E-12 | 0.27 | 1.29 (1.19-1.40) | 1.3E-09 | 0.78 | | 0.93 | 0.87 | 0.99 |  |
| rs28462675 | 7 | 50406172 | 7:50409989 | A | G | 1.58 (1.39-1.78) | 6.4E-13 | 0.27 | 1.29 (1.19-1.41) | 9.0E-10 | 0.83 | | 0.95 | 0.98 | 0.99 |  |
| rs114373434 | 7 | 50407233 | 7:50409989 | C | G | 1.71 (1.48-1.96) | 4.8E-14 | 0.26 | 1.30 (1.19-1.41) | 7.8E-10 | 0.54 | | 1 | 0.94 | 0.99 |  |
| rs28696237 | 7 | 50408133 | 7:50409989 | C | G | 1.58 (1.4-1.8) | 1.0E-12 | 0.27 | 1.30 (1.19-1.41) | 7.2E-10 | 0.86 | | 0.96 | 0.99 | 1 |  |
| rs10230978 | 7 | 50409446 | 7:50409989 | G | A | 1.57 (1.39-1.78) | 1.4E-12 | 0.27 | 1.30 (1.19-1.41) | 9.1E-10 | 0.82 | | 0.93 | 0.98 | 0.99 |  |
| rs10272724 | 7 | 50409515 | 7:50409989 | T | C | 1.57 (1.39-1.78) | 1.4E-12 | 0.27 | 1.30 (1.19-1.41) | 8.4E-10 | 0.82 | | 0.93 | 0.98 | 0.99 |  |
| rs17133805 | 7 | 50409816 | 7:50409989 | T | G | 1.57 (1.39-1.78) | 1.4E-12 | 0.27 | 1.30 (1.19-1.41) | 9.4E-10 | 0.82 | | 0.93 | 0.98 | 0.99 |  |
| rs62445869 | 7 | 50409913 | 7:50409989 | G | A | 1.67 (1.45-1.92) | 3.4E-13 | 0.26 | 1.29 (1.19-1.41) | 1.1E-09 | 0.54 | | 1 | 0.97 | 1 |  |
| rs17133807 | 7 | 50409989 | 7:50409989 | G | A | 1.62 (1.43-1.83) | 3.3E-14 | 0.27 | 1.30 (1.19-1.41) | 9.0E-10 | 1 | | 1 | 1 | 1 |  |
| rs10740053 | 10 | 61942825 | 10:61961417 | C | T | 0.60 (0.53-0.67) | 9.2E-18 | 0.57 | 0.58 (0.54-0.63) | 4.0E-43 | 0.70 | | 0.91 | 0.90 | 0.96 |  |
| rs10740054 | 10 | 61942827 | 10:61961417 | T | A | 0.60 (0.53-0.67) | 8.8E-18 | 0.57 | 0.58 (0.54-0.63) | 4.0E-43 | 0.70 | | 0.91 | 0.90 | 0.96 |  |
| rs4948492 | 10 | 61959980 | 10:61961417 | C | T | 0.60 (0.53-0.67) | 1.9E-17 | 0.55 | 0.57 (0.53-0.62) | 1.9E-45 | 0.95 | | 0.99 | 0.99 | 0.99 |  |
| rs7090445 | 10 | 61961417 | 10:61961417 | C | T | 0.58 (0.52-0.66) | 3.1E-18 | 0.56 | 0.57 (0.53-0.62) | 4.7E-45 | 1 | | 1 | 1 | 1 |  |
| rs4245595 | 10 | 61963136 | 10:61961417 | C | T | 0.60 (0.53-0.67) | 2.1E-17 | 0.55 | 0.57 (0.53-0.62) | 3.9E-45 | 0.94 | | 0.99 | 0.99 | 1 |  |
| rs10821936 | 10 | 61963818 | 10:61961417 | C | T | 0.60 (0.54-0.68) | 6.7E-17 | 0.55 | 0.57 (0.53-0.62) | 4.1E-45 | 0.93 | | 0.98 | 0.98 | 0.99 |  |
| rs10821937 | 10 | 61964150 | 10:61961417 | C | T | 0.60 (0.54-0.68) | 8.9E-17 | 0.55 | 0.57 (0.53-0.62) | 3.4E-45 | 0.92 | | 0.96 | 0.98 | 0.99 |  |
| rs116677565 | 15 | 24174608 | 15:24174608 | T | C | 2.39 (1.76-3.23) | 2.1E-08 | 0.003 | 1.87 (1.03-3.40) | 0.041 | 1 | | 1 | 1 | 1 |  |
| rs5002270 | 15 | 24178668 | 15:24174608 | G | A | 2.39 (1.76-3.23) | 2.1E-08 | 0.003 | 1.88 (1.03-3.42) | 0.039 | 0.98 | | 1 | 1 | 1 |  |
| rs145683427 | 15 | 24178703 | 15:24174608 | C | A | 2.42 (1.77-3.32) | 3.8E-08 | 0.003 | 1.88 (1.03-3.42) | 0.039 | 0.89 | | 0.98 | 1 | 1 |  |

Abbreviations: CCRLP, California Cancer Records Linkage Project; rsid, genetic variant identifier using dbSNP build 151; CHR, chromosome; BP, base position, GRCh38 (hg38) build; OR, odds ratio; CI, confidence interval; P, p-value; AFR, African genetic ancestry; AMR, Admixed American genetic ancestry. Odds ratios (ORs) and 95% confidence intervals (CIs) are from logistic regression models. All presented p-values are from two-sided statistical tests and are not adjusted for multiple testing.

**Supplementary Table 13**: Replication of B-ALL associations identified in African American children (ADMIRAL meta-analysis) in an African American population in CCRLP (P<0.05)

|  |  |  |  | CCRLP, African American | | ADMIRAL, AFR | | | | | |
| --- | --- | --- | --- | --- | --- | --- | --- | --- | --- | --- | --- |
| CHR | BP | Other allele | Tested allele | OR (95% CI) | P | Meta-analysis OR (95% CI) | Meta-analysis P | Discovery OR (95% CI) | Discovery P | Replication OR (95% CI) | Replication P |
| 5 | 111791133 | G | A | 2.07 (1.09-3.93) | 0.026 | 2.63 (1.94-3.55) | 3.91E-10 | 2.9 (2.09-4.04) | 2.2E-10 | 1.53 (0.76-2.30) | 0.28 |
| 7 | 50394709 | T | A | 1.57 (1.17-2.11) | 2.9E-03 | 1.56 (1.38-1.76) | 8.00E-13 | 1.62 (1.41-1.86) | 3.9E-12 | 1.34 (1.08-1.60) | 0.031 |
| 7 | 50398132 | A | G | 1.57 (1.17-2.11) | 2.8E-03 | 1.56 (1.38-1.76) | 8.53E-13 | 1.62 (1.41-1.86) | 4.1E-12 | 1.34 (1.08-1.60) | 0.031 |
| 7 | 50398606 | A | G | 1.60 (1.19-2.15) | 1.7E-03 | 1.56 (1.38-1.76) | 8.07E-13 | 1.62 (1.41-1.86) | 4.1E-12 | 1.34 (1.08-1.60) | 0.029 |
| 7 | 50398997 | G | A | 1.57 (1.17-2.11) | 2.8E-03 | 1.55 (1.38-1.75) | 1.08E-12 | 1.62 (1.41-1.85) | 5.4E-12 | 1.34 (1.08-1.60) | 0.031 |
| 7 | 50399099 | A | G | 1.57 (1.17-2.11) | 2.8E-03 | 1.55 (1.38-1.75) | 1.02E-12 | 1.62 (1.41-1.85) | 5.4E-12 | 1.34 (1.08-1.60) | 0.029 |
| 7 | 50401254 | G | T | 1.59 (1.18-2.14) | 2.1E-03 | 1.54 (1.37-1.74) | 2.95E-12 | 1.61 (1.4-1.84) | 1.0E-11 | 1.32 (1.05-1.59) | 0.040 |
| 7 | 50401853 | C | T | 1.58 (1.18-2.13) | 2.4E-03 | 1.54 (1.37-1.74) | 2.50E-12 | 1.61 (1.4-1.85) | 8.8E-12 | 1.32 (1.05-1.59) | 0.040 |
| 7 | 50402283 | T | C | 1.58 (1.18-2.13) | 2.4E-03 | 1.55 (1.37-1.75) | 1.98E-12 | 1.61 (1.41-1.85) | 7.5E-12 | 1.32 (1.05-1.59) | 0.039 |
| 7 | 50402906 | T | G | 1.58 (1.18-2.13) | 2.4E-03 | 1.55 (1.37-1.75) | 1.98E-12 | 1.61 (1.41-1.85) | 7.5E-12 | 1.32 (1.05-1.59) | 0.039 |
| 7 | 50403915 | G | A | 1.55 (1.15-2.09) | 4.1E-03 | 1.55 (1.37-1.75) | 2.04E-12 | 1.62 (1.41-1.85) | 6.8E-12 | 1.32 (1.05-1.59) | 0.042 |
| 7 | 50404626 | G | A | 1.53 (1.14-2.06) | 5.1E-03 | 1.55 (1.37-1.75) | 1.91E-12 | 1.62 (1.41-1.86) | 6.6E-12 | 1.32 (1.05-1.59) | 0.042 |
| 7 | 50405144 | C | T | 1.55 (1.15-2.09) | 4.1E-03 | 1.55 (1.37-1.75) | 2.16E-12 | 1.62 (1.41-1.85) | 7.2E-12 | 1.32 (1.05-1.59) | 0.042 |
| 7 | 50405553 | A | G | 1.55 (1.15-2.09) | 4.1E-03 | 1.55 (1.37-1.75) | 1.97E-12 | 1.62 (1.41-1.85) | 7.2E-12 | 1.32 (1.05-1.59) | 0.039 |
| 7 | 50405592 | T | C | 1.55 (1.15-2.09) | 4.0E-03 | 1.55 (1.37-1.75) | 1.70E-12 | 1.62 (1.41-1.86) | 5.6E-12 | 1.32 (1.05-1.59) | 0.042 |
| 7 | 50406172 | A | G | 1.53 (1.14-2.07) | 5.2E-03 | 1.58 (1.39-1.78) | 6.36E-13 | 1.66 (1.44-1.9) | 1.2E-12 | 1.30 (1.03-1.57) | 0.059 |
| 7 | 50408133 | C | G | 1.48 (1.09-2.02) | 0.013 | 1.58 (1.4-1.8) | 1.01E-12 | 1.66 (1.44-1.92) | 2.3E-12 | 1.32 (1.04-1.60) | 0.054 |
| 7 | 50409446 | G | A | 1.49 (1.09-2.03) | 0.012 | 1.57 (1.39-1.78) | 1.36E-12 | 1.67 (1.45-1.92) | 6.7E-13 | 1.24 (0.96-1.52) | 0.14 |
| 7 | 50409515 | T | C | 1.49 (1.09-2.02) | 0.012 | 1.57 (1.39-1.78) | 1.36E-12 | 1.67 (1.45-1.92) | 6.7E-13 | 1.24 (0.96-1.52) | 0.14 |
| 7 | 50409816 | T | G | 1.49 (1.09-2.03) | 0.012 | 1.57 (1.39-1.78) | 1.36E-12 | 1.67 (1.45-1.92) | 6.7E-13 | 1.24 (0.96-1.52) | 0.14 |
| 7 | 50409989 | G | A | 1.51 (1.12-2.05) | 7.66E-03 | 1.62 (1.43-1.83) | 3.34E-14 | 1.72 (1.5-1.98) | 2.1E-14 | 1.27 (0.99-1.55) | 0.093 |
| 15 | 24178668 | G | A | 1.93 (1.04-3.57) | 0.037 | 2.39 (1.76-3.23) | 2.07E-08 | 2.33 (1.63-3.33) | 3.0E-06 | 2.54 (1.95-3.13) | 1.9E-03 |
| 15 | 24188999 | T | C | 2.13 (1.22-3.73) | 7.70E-03 | 1.89 (1.41-2.54) | 1.76E-05 | 1.94 (1.39-2.72) | 1.2E-04 | 1.76 (1.18-2.34) | 0.056 |

Abbreviations: CCRLP, California Cancer Records Linkage Project; CHR, chromosome; BP, base position, GRCh38 (hg38) build; OR, odds ratio; CI, confidence interval; P, p-value; AFR, African genetic ancestry. Odds ratios (ORs) and 95% confidence intervals (CIs) are from logistic regression models. All presented p-values are from two-sided statistical tests and are not adjusted for multiple testing.

**Supplementary Table 14**: Oligonucleotide sequences for dual luciferase assays

| rsid | Ref./Alt. | Oligonucleotide coordinates (hg38) | Size (bp) | Reference allele sequence (SNP allele) | Alternative allele sequence (SNP allele) | Plasmid backbone DNA sequencing primer |
| --- | --- | --- | --- | --- | --- | --- |
| rs77632976 | C/T | chr2:34828280-34828580 | 301 | CTATACATTCTTATGTGAATTGTTACCAACTTATTTCTCATTTTTGCCTATGCCTCTCTAGTCTACCATGCTTCAGCTATAGTAGCCTTCAATCAGTTTCATAATACTCTATATCGGTCACTTATGCCACAGGGCTGTTGGACATATTCTCGCTGCCTGGATTATACATCCTTCTCCTCTTTGTCTAACTTATTATCGTCCTTATAAGTCTCAGACACAGTTTTATTTTCTCAGAGAACACTTTTGACTCTTCTGAGAATGATGCCTCTGTTAAAAATGGCATTTAGTTAGTAATTAAGTG | CTATACATTCTTATGTGAATTGTTACCAACTTATTTCTCATTTTTGCCTATGCCTCTCTAGTCTACCATGCTTCAGCTATAGTAGCCTTCAATCAGTTTCATAATACTCTATATCGGTCACTTATGCCACAGGGCTGTTGGACATATTCTTGCTGCCTGGATTATACATCCTTCTCCTCTTTGTCTAACTTATTATCGTCCTTATAAGTCTCAGACACAGTTTTATTTTCTCAGAGAACACTTTTGACTCTTCTGAGAATGATGCCTCTGTTAAAAATGGCATTTAGTTAGTAATTAAGTG | CTAGCAAAATAGGCTGTCCC |
| rs112113758 | G/T | chr3:2292082-2292382 | 301 | TCCATCACATAATTAACCGTTAAAATGTCTCATGCCCATTCCCTGTCTCATTTGTAAAAAGTGGTACTTGTATTGTCACTTCTACGGCACTTATCTGTCCCACACATTTTGGCAACAGATTATTTTTCTTCTGGATTCTTCTCTAATAGTGTTATATTTATAAGCTTGTCTCTTGCACAACAGAATGAACTTCACAAGGGCATTGGCTTTTCTTCTATCTCTTAGTGAATAATTGTCACTTAGGATTTATTGAAACACAGTACCAAACATGTATTAGGTACCCCACTGATATTTGTTAACT | TCCATCACATAATTAACCGTTAAAATGTCTCATGCCCATTCCCTGTCTCATTTGTAAAAAGTGGTACTTGTATTGTCACTTCTACGGCACTTATCTGTCCCACACATTTTGGCAACAGATTATTTTTCTTCTGGATTCTTCTCTAATAGTTTTATATTTATAAGCTTGTCTCTTGCACAACAGAATGAACTTCACAAGGGCATTGGCTTTTCTTCTATCTCTTAGTGAATAATTGTCACTTAGGATTTATTGAAACACAGTACCAAACATGTATTAGGTACCCCACTGATATTTGTTAACT | CTAGCAAAATAGGCTGTCCC |
| rs28568357 | T/C | chr4:183347664-183347964 | 301 | GGTTCTAGGGCCTTTGGACTCTGGGGCTTGTATTAGAGCCCTTCCTCCAACCCCAGCCCCCAGGCCCCAGGGGTTGGGGTTCTCAGGGCTTCAGCCTCAGACTGAATTGCATCACCAGCATTCCTGGATCTTGAGCCGGCAGAGAGCAGATCATGGCACTTCTCAGCCTCCTTCTCATAACCCATGAGCCAAACAGTAGATCGCCTCTTCTCTATGCACAGAGAGCCTGGGGTTCTGTTTCTCCAGAGGACCCTAATACAGCTGCCATTCCACCAGACAGCGCCTTTCCTCTTGTCTAGCT | GGTTCTAGGGCCTTTGGACTCTGGGGCTTGTATTAGAGCCCTTCCTCCAACCCCAGCCCCCAGGCCCCAGGGGTTGGGGTTCTCAGGGCTTCAGCCTCAGACTGAATTGCATCACCAGCATTCCTGGATCTTGAGCCGGCAGAGAGCAGACCATGGCACTTCTCAGCCTCCTTCTCATAACCCATGAGCCAAACAGTAGATCGCCTCTTCTCTATGCACAGAGAGCCTGGGGTTCTGTTTCTCCAGAGGACCCTAATACAGCTGCCATTCCACCAGACAGCGCCTTTCCTCTTGTCTAGCT | CTAGCAAAATAGGCTGTCCC |
| rs183221417 | T/C | chr5:100101893-100102193 | 1001 | CTTTAAATGATGAACCAAGAAAGACACTAAGAGGGAAAATCTATGAATATCAACAGTGCCTGTCATTCAGTGAATGAACTGTAATTGCTGACACTTTCCCATTTGGAATAATGGGAATGTCAGCCTAAATACAGATCAGATTCAGACATCTTTCAACAAGATGTCTCTAAGGAAATACTATGTGGACATGTGTTCAGGAAGCAGTATTCAGTGGTAATGGCAGAAATTCATTCTGCAGTAAATGTTTAAAACGGTTGACAGTTTTAATATGTTTTACCTAAAGATACAGTCATTTTACTGAAGATTACTTACACTGTGGGAAATAAAAAATGTTCTTAGTAGACTGAAAAATCTATGTCCCCAAATCTCAGAGGAATCAATGAGCATTTTATTCAGCCTGCAATGAGCAGTTGTGTGAAAGAATTCCAGGACTTGCAGAGCGAATTAAACATTAATACAAATTCCCATCATGTCACTTTGTGTGTGTGTCTGTGTGTGTGTGTATGTGTATCTATATATATATATGTAAGTTTGACCTGATTTTCTTGATAGTGGGTAAATGTGCAACATTTGGAAGAATAAACTTAAACTGTTAATTTGATGAGTTACTATGAATATAGAAAAGAACAAAAACCAATCAAATTTATGGGATGATATTTCTCTTAAGACAGTTGAATAACACCTATAAAATATATCTGATGTGTAAGCTACATTTTTAGAAAGGTGGTGATTGTAGACACAGTGTTCTAAAACATAAAATTATGTGGCACTTCATTGAGTGTTTACCTTTTCAAGGGTCAGTGGTAACATTTTCATCAATCCTTAATGAACACACTGATTTTCCTAATTCATCAGGCATATTCTTAAATATCTTGGATACAAACTCACTAAACTTTTTTTCTTTCTATCATTTTATCTTTATCTTTGAGCTCATTTGAATTATGCCCATTTTGCTAGAAGCAGCTCAACATGGGAGATCAAGTTTTCATATAAGCAATAAA | CTTTAAATGATGAACCAAGAAAGACACTAAGAGGGAAAATCTATGAATATCAACAGTGCCTGTCATTCAGTGAATGAACTGTAATTGCTGACACTTTCCCATTTGGAATAATGGGAATGTCAGCCTAAATACAGATCAGATTCAGACATCTTTCAACAAGATGTCTCTAAGGAAATACTATGTGGACATGTGTTCAGGAAGCAGTATTCAGTGGTAATGGCAGAAATTCATTCTGCAGTAAATGTTTAAAACGGTTGACAGTTTTAATATGTTTTACCTAAAGATACAGTCATTTTACTGAAGATTACTTACACTGTGGGAAATAAAAAATGTTCTTAGTAGACTGAAAAATCTATGTCCCCAAATCTCAGAGGAATCAATGAGCATTTTATTCAGCCTGCAATGAGCAGTTGTGTGAAAGAATTCCAGGACTTGCAGAGCGAATTAAACATTAATACAAATTCCCATCATGTCACTTTGTGTGTGTGTCTGTGTGTGTGCGTATGTGTATCTATATATATATATGTAAGTTTGACCTGATTTTCTTGATAGTGGGTAAATGTGCAACATTTGGAAGAATAAACTTAAACTGTTAATTTGATGAGTTACTATGAATATAGAAAAGAACAAAAACCAATCAAATTTATGGGATGATATTTCTCTTAAGACAGTTGAATAACACCTATAAAATATATCTGATGTGTAAGCTACATTTTTAGAAAGGTGGTGATTGTAGACACAGTGTTCTAAAACATAAAATTATGTGGCACTTCATTGAGTGTTTACCTTTTCAAGGGTCAGTGGTAACATTTTCATCAATCCTTAATGAACACACTGATTTTCCTAATTCATCAGGCATATTCTTAAATATCTTGGATACAAACTCACTAAACTTTTTTTCTTTCTATCATTTTATCTTTATCTTTGAGCTCATTTGAATTATGCCCATTTTGCTAGAAGCAGCTCAACATGGGAGATCAAGTTTTCATATAAGCAATAAA | CTAGCAAAATAGGCTGTCCC |
| rs867166159^a^ | G/T | chr9:191001-191291 | 291 | GTATGTGGAAGCAATATGTTTGG**G**ATGATACACACCCTTGCAATAACATGAACTACCTCAGGGAGTACTATAGATGACAGAGACAATGGGGGAAGAGGAGCAATGTTACCTTAAAAAAAAACTTTGCATCCTATTACT**G**GTTACAAGAGGCATATAAAATTATTTTTAAAATTTTAAATAAATATGTTAGCAACCTTTGTATGGATGGAAAATTTTAAATATGTATGAAAATACTGATTGCGTATACCTTTTGAAATTCTACTGATAGGCATTTATCCTACATATTTACAT | GTATGTGGAAGCAATATGTTTGG**A**ATGATACACACCCTTGCAATAACATGAACTACCTCAGGGAGTACTATAGATGACAGAGACAATGGGGGAAGAGGAGCAATGTTACCTTAAAAAAAAACTTTGCATCCTATTACT**T**GTTACAAGAGGCATATAAAATTATTTTTAAAATTTTAAATAAATATGTTAGCAACCTTTGTATGGATGGAAAATTTTAAATATGTATGAAAATACTGATTGCGTATACCTTTTGAAATTCTACTGATAGGCATTTATCCTACATATTTACAT | CTAGCAAAATAGGCTGTCCC |
| rs112269413^b^ | G/A | chr11:54651352-54651752 | 401 | CAAACATGATCATGTCATCTACAGACATTGACAGTTTTATTTCTTCCTATCAGTATACTTCTTATTCTTTTTCTTGTCTTATTACATTAGCTAGGACTTCCAGGACAATGTTGACAAGAAGTGATGAGAAGAGACATTCTTGCCTTTTTCCTAGTCTTAGTAGGAAAGCTTCAAGTTTCTCACCATTAAGTGTGATGCGG**G**CTGTAGATTCTTACTTTACAGATTTTTTTTTAGAATCATAAATAGGTGTTAGATTTTTTTAAATGCTTTTTCTGCATCTATTAATATGGTAATTTGATTTTTTCCCTTTAACATTTCACACAATGGATTATGTTAATTGATTTTTGAATGTTGATCTCACCCTGCATACCTGGACTAAATTGTACACCAGGATCTACTGA | CAAACATGATCATGTCATCTACAGACATTGACAGTTTTATTTCTTCCTATCAGTATACTTCTTATTCTTTTTCTTGTCTTATTACATTAGCTAGGACTTCCAGGACAATGTTGACAAGAAGTGATGAGAAGAGACATTCTTGCCTTTTTCCTAGTCTTAGTAGGAAAGCTTCAAGTTTCTCACCATTAAGTGTGATGCGG**A**CTGTAGATTCTTACTTTACAGATTTTTTTTTAGAATCATAAATAGGTGTTAGATTTTTTTAAATGCTTTTTCTGCATCTATTAATATGGTAATTTGATTTTTTCCCTTTAACATTTCACACAATGGATTATGTTAATTGATTTTTGAATGTTGATCTCACCCTGCATACCTGGACTAAATTGTACACCAGGATCTACTGA | CTAGCAAAATAGGCTGTCCC |
| rs76135126 | T/C | chr11:119032924-119033224 | 351 | CATGCCCCACCATGCCCAGCTAATTTTGTAATTTTAGTAGGGGTAGGGTTTCTCCATGTTGGTCAGGCTGGTCTCAAACTCCTGACTTCAGGTGATCTGCCCCCCTTGGCCTCCCAAAGTGCTGGGATTACAGGCATGAACAACTGCACCCAGCCAACCCGGTTAATTTTTCTATTTTTTGTAGAGACAAGAGTTTCACCATATTGGCCAGGCTGGCCTCGAAATCCTGAGCTCAAGGGATCCACCTGCCTTGGTCTTCCAATATCCTGGAATTACAGGCATGAGCCACTGTGCCTGGTCGATGGTGTTTATTTGTTTGTTTGTTTGTTTTTGAGACAGAGTCTTGCTCTG | CATGCCCCACCATGCCCAGCTAATTTTGTAATTTTAGTAGGGGTAGGGTTTCTCCATGTTGGTCAGGCTGGTCTCAAACTCCTGACTTCAGGTGATCTGCCCCCCTTGGCCTCCCAAAGTGCTGGGATTACAGGCATGAACAACTGCACCCAGCCAACCCGGTTAATTTTTCTATCTTTTGTAGAGACAAGAGTTTCACCATATTGGCCAGGCTGGCCTCGAAATCCTGAGCTCAAGGGATCCACCTGCCTTGGTCTTCCAATATCCTGGAATTACAGGCATGAGCCACTGTGCCTGGTCGATGGTGTTTATTTGTTTGTTTGTTTGTTTTTGAGACAGAGTCTTGCTCTG | CTAGCAAAATAGGCTGTCCC |
| rs113299167 | C/T | chr12:129961691-129961991 | 301 | GACCATCAACCCTTGCTTAAGGGCAGGACACCAGACTGAATCATGTCAGCATCGCTTCCAGATTCGAGATTCTACAGTTTTCTAAAATGCTGGCAAATCTGAAATCTGAGCTCTTCTCCCCACAGACGCAGGAGTCCACGATGTGAGTGGCATCCACAGCTCCCCGTGGTGATTTGAAGGAATGCCCCTTGGCATCACCAGCCTCATCCTGACTGTGGCCATCACTGGAAATTCATATGCAAAGAAAAGAGTCTTTCTTTTCTTTCTTCCTTCCTGCCTTCCTTTCTTTTCTTTCTCTCTT | GACCATCAACCCTTGCTTAAGGGCAGGACACCAGACTGAATCATGTCAGCATCGCTTCCAGATTCGAGATTCTACAGTTTTCTAAAATGCTGGCAAATCTGAAATCTGAGCTCTTCTCCCCACAGACGCAGGAGTCCACGATGTGAGTGGTATCCACAGCTCCCCGTGGTGATTTGAAGGAATGCCCCTTGGCATCACCAGCCTCATCCTGACTGTGGCCATCACTGGAAATTCATATGCAAAGAAAAGAGTCTTTCTTTTCTTTCTTCCTTCCTGCCTTCCTTTCTTTTCTTTCTCTCTT | CTAGCAAAATAGGCTGTCCC |
| rs116677565 | T/C | chr15:24174458-24174758 | 1001 | ATTACTGTTTGTTTTCTGTTCTAGGATCTCTTCCAGGATAACACCTTTAGATATCATGTCTCCTTAAGCTTTTTTTGTCTCTGCAGTTTCTCTGAATTTCCATGTTTTTCGTGGCATTGACATCCTCCATTTAGGATTTCTATGATTGTTTTTACTAACAATTAGACTGAATTTGTAGATGTGGAAGAAGAAGAGACAGGAGTGAGTGTAATACTCATTATACCATGGGCATATATTCTCAAAATGCTTTTATCACTATTGATACTAATTTGAGCACCTGGATGAGGCAGTGTGTGTCCAGTTTCTCCACTGTAAAATTAATTTTCCCCCTATCCACGTCGTACTTTTTGAAAAAAACGTCACTATGCACAGCGCATACTTAATATTGGGGAATTATTCTTCACCTCCTTAGGGCAGAACTTGTATAGTATCGTTTGTATTTCATTATCATAGGCAATGTATCTCTTCTCCAGCATTTATTTAGTCAATTATTTATATTATATGGAATATTGGCAAATAAAAAATATTTATTTCCCCTGTGATTAGACTAGTTTATTGTATTGTTCAAATTGTGCTAGTGCTGGTCATTAGAAGGCCTTTCAGTCAGCTGCTTTACCACTTTGAAATACCCATATTATTGCTATTTGATTTGGTTTGTGTGGTGTGTTGTTTGGTTGTGTTGTTTAACTTTTTCTTACTTTCTGGCTCTACAAGGTACTTAAGCTTAATAGTGTTGATTCCCTTCCCAGCCATGCCATTTACCATTTTCCCCCAAATTCCTAGTTCCTTTTTTGGTGAATGGCATTAGAAACAAAGGTCTCGGCAATGGTGTTGTCATTTCTGCCAGGCGTCTGTTAATTCTATGTTTTGTCATCTGACAGAACAAAGGATTATATGTATGTATGTAATTCACACTCACATATGTATATGCATTTCTACAACATTTCTCCATTTATTCCTATGTAAAGTTCAGCATGAATTCTTATTAATGTTTCCAGCAC | ATTACTGTTTGTTTTCTGTTCTAGGATCTCTTCCAGGATAACACCTTTAGATATCATGTCTCCTTAAGCTTTTTTTGTCTCTGCAGTTTCTCTGAATTTCCATGTTTTTCGTGGCATTGACATCCTCCATTTAGGATTTCTATGATTGTTTTTACTAACAATTAGACTGAATTTGTAGATGTGGAAGAAGAAGAGACAGGAGTGAGTGTAATACTCATTATACCATGGGCATATATTCTCAAAATGCTTTTATCACTATTGATACTAATTTGAGCACCTGGATGAGGCAGTGTGTGTCCAGTTTCTCCACTGTAAAATTAATTTTCCCCCTATCCACGTCGTACTTTTTGAAAAAAACGTCACTATGCACAGCGCATACTTAATATTGGGGAATTATTCTTCACCTCCTTAGGGCAGAACTTGTATAGTATCGTTTGTATTTCATTATCATAGGCAATGTATCTCTTCTCCAGCATTTATTTAGTCAATTATTTATATTACATGGAATATTGGCAAATAAAAAATATTTATTTCCCCTGTGATTAGACTAGTTTATTGTATTGTTCAAATTGTGCTAGTGCTGGTCATTAGAAGGCCTTTCAGTCAGCTGCTTTACCACTTTGAAATACCCATATTATTGCTATTTGATTTGGTTTGTGTGGTGTGTTGTTTGGTTGTGTTGTTTAACTTTTTCTTACTTTCTGGCTCTACAAGGTACTTAAGCTTAATAGTGTTGATTCCCTTCCCAGCCATGCCATTTACCATTTTCCCCCAAATTCCTAGTTCCTTTTTTGGTGAATGGCATTAGAAACAAAGGTCTCGGCAATGGTGTTGTCATTTCTGCCAGGCGTCTGTTAATTCTATGTTTTGTCATCTGACAGAACAAAGGATTATATGTATGTATGTAATTCACACTCACATATGTATATGCATTTCTACAACATTTCTCCATTTATTCCTATGTAAAGTTCAGCATGAATTCTTATTAATGTTTCCAGCAC | CTAGCAAAATAGGCTGTCCC |
| rs17133807 (control) | G/A | chr7:50409839-50410139 | 301 | TCCAGCTCCAGGAGTGGCCCTGCAGGTCCTGGAGCAACAGCCTGGCCCCTGCCCACGCCCCCTCCTCCCTGGCCGCAGGCCCTGGGCCTGGGCTGAGCAGATGGGCCCTGGCAACTCGGTGAATCGGAACTATGGGAAGCAGATGCACCCGCCATGGGTCCCCGGGCACAACTGGAACCTGCTGGGGGAGGGAATTTGCATAATAAGGATAATTTGGTACCAAAACTGTCCCCTGAAGTGAACTGCATGGATCTCGATACAGTTAACAAATTTCACTTCTTCTTTCCCATGTTTTGTCAAA | TCCAGCTCCAGGAGTGGCCCTGCAGGTCCTGGAGCAACAGCCTGGCCCCTGCCCACGCCCCCTCCTCCCTGGCCGCAGGCCCTGGGCCTGGGCTGAGCAGATGGGCCCTGGCAACTCGGTGAATCGGAACTATGGGAAGCAGATGCACCCACCATGGGTCCCCGGGCACAACTGGAACCTGCTGGGGGAGGGAATTTGCATAATAAGGATAATTTGGTACCAAAACTGTCCCCTGAAGTGAACTGCATGGATCTCGATACAGTTAACAAATTTCACTTCTTCTTTCCCATGTTTTGTCAAA | CTAGCAAAATAGGCTGTCCC |
| rs7090445^c^ (control) | C/T | chr10:61961267-61961567 | 301 | TAATGGTGAAGGTAAATATATAATCCTTAAAATAAAAAATATGTTTATCCATGTACCACCTAACATTGACTTGGGTACCGCTAGTGTATACCACAGTTAAGTCATTTCCTTTGAGGCTTTTCTCTTGGGCAACAGCCTAACCTAGGTTATCGATAGCTTTGAGACCTTCTGAGACCATGCAAGCCTCGTGTCAACAACAAGCTAGAAAGGGTTGGAACCGGTTGCGCTGAATTAATCTATAGGCTCAGAAGTGTAACTGCCACACAGATGTGAAGTCTGAAAATAACCCACCGTGCACCTG | TAATGGTGAAGGTAAATATATAATCCTTAAAATAAAAAATATGTTTATCCATGTACCACCTAACATTGACTTGGGTACCGCTAGTGTATACCACAGTTAAGTCATTTCCTTTGAGGCTTTTCTCTTGGGCAACAGCCTAACCTAGGTTATTGATAGCTTTGAGACCTTCTGAGACCATGCAAGCCTCGTGTCAACAACAAGCTAGAAAGGGTTGGAACCGGTTGCGCTGAATTAATCTATAGGCTCAGAAGTGTAACTGCCACACAGATGTGAAGTCTGAAAATAACCCACCGTGCACCTG | CTAGCAAAATAGGCTGTCCC |

Abbreviations: rsid, variant identifier using dbSNP build 151; bp, base pairs.

1. Two-stage PCR was employed to amplify DNA centered the variant using genomic DNA from the Coriell Institute for Medical Research (#NA19704) heterozygous for the variant. PCR primer stage 1: ACATGCTTGTATGTGCATGGG (F); TTTACCAGGTGCGTGCTCTG (R). PCR primer stage 2: ATGCTGATATATGTATGTGGAAGC (F); GTAAATATGTAGGATAAATGCCTATCAGTAG (R). Variants rs558007269 (G/A) and rs867166159 (G/T) variants were tested together as a haplotype.
2. Two-stage PCR was employed to amplify DNA centered the variant using genomic DNA from the Coriell Institute for Medical Research (#NA19704) heterozygous for the variant. PCR primer stage 1: TCTGGCACGTATGAGCTTCC (F); GTGATACTTAGCCGAGGTCCG (R). PCR primer stage 2: CAAACATGATCATGTCATCTACAGAC (F); TCAGTAGATCCTGGTGTACAATTTAG (R).
3. Reference/alternative alleles for all variants correspond to non-effect and effect alleles described throughout the manuscript, respectively, except for rs7090445 where reference=effect allele and alternative=non-effect allele.

**Supplementary Table 15**: Relative allele-specific luciferase assay reporter activity in 697 and GM12878 cell lines

| Cell line | rsid | Mean (NEA) | Mean (EA) | Mean of difference (EA - NEA) | 95% CI LB | 95% CI UB | P |
| --- | --- | --- | --- | --- | --- | --- | --- |
| 697 | rs77632976 | 1.00 | 1.37 | 0.37 | 0.22 | 0.52 | 1.5x10^-3^ |
|  | rs112113758 | 1.00 | 1.82 | 0.82 | 0.60 | 1.04 | 2.1x10^-4^ |
|  | rs116677565 | 1.00 | 0.87 | -0.13 | -0.34 | 0.09 | 0.19 |
|  | rs183221417 | 1.00 | 1.08 | 0.08 | -0.14 | 0.31 | 0.38 |
|  | rs76135126 | 1.00 | 1.04 | 0.04 | -0.27 | 0.36 | 0.73 |
|  | rs28568357 | 1.00 | 0.92 | -0.08 | -0.21 | 0.05 | 0.17 |
|  | rs7090445 | 2.36 | 1.00 | -1.36 | -2.55 | -0.17 | 0.032 |
|  | rs17133807 | 1.00 | 0.81 | -0.19 | -0.36 | -0.02 | 0.036 |
|  | rs113299167 | 1.00 | 1.15 | 0.15 | 0.03 | 0.27 | 0.025 |
|  | rs112269413 | 1.00 | 1.14 | 0.14 | 0.06 | 0.22 | 5.2x10^-3^ |
|  | rs867166159 | 1.00 | 1.37 | 0.37 | 0.27 | 0.47 | 2.4x10^-4^ |
| GM12878 | rs77632976 | 1.00 | 1.28 | 0.28 | 0.13 | 0.43 | 5.1x10^-3^ |
|  | rs112113758 | 1.00 | 0.74 | -0.26 | -0.41 | -0.10 | 7.9x10^-3^ |
|  | rs116677565 | 1.00 | 0.93 | -0.07 | -0.36 | 0.22 | 0.56 |
|  | rs183221417 | 1.00 | 1.13 | 0.13 | -0.05 | 0.30 | 0.12 |
|  | rs76135126 | 1.00 | 1.12 | 0.12 | -0.05 | 0.28 | 0.14 |
|  | rs28568357 | 1.00 | 1.11 | 0.11 | -0.07 | 0.29 | 0.16 |
|  | rs7090445 | 1.14 | 1.00 | -0.14 | -0.22 | -0.06 | 5.3x10^-3^ |
|  | rs17133807 | 1.00 | 1.28 | 0.28 | 0.00 | 0.57 | 0.050 |
|  | rs113299167 | 1.00 | 1.18 | 0.18 | 0.02 | 0.35 | 0.038 |
|  | rs112269413 | 1.00 | 0.76 | -0.24 | -0.36 | -0.13 | 3.0x10^-3^ |
|  | rs867166159 | 1.00 | 1.20 | 0.20 | 0.11 | 0.29 | 2.2x10^-3^ |

Abbreviations: rsid, variant identifier using dbSNP build 151; EA, effect (risk) allele; NEA, non-effect (reference) allele; CI, confidence interval; LB, lower bound; UB, upper bound. Note that for rs7090445, reference=effect allele and alternative=non-effect allele and alternative allele activity was normalized to reference allele activity. P-values from two-sided paired t-tests are provided and are not corrected for multiple testing.

**Supplementary Table 16**: Estimates of SNP-based heritability of B-ALL using the GREML-LDMS-I method in African and European descent populations

| Sample | h^2^ | SE |
| --- | --- | --- |
| Common variants (MAF>0.05) |  |  |
| African ancestry (ADMIRAL) | 0.360 | 0.042 |
| Non-Latino White or European descent (Jeon *et al.*^10^) | 0.203 | 0.032 |
| Common variants (MAF>0.05), excluding all known B-ALL risk loci |  |  |
| African ancestry (ADMIRAL) | 0.342 | 0.042 |

Abbreviations: MAF, minor allele frequency

**Supplementary Table 17**: Familial relative risk (FRR) explained by known and novel B-ALL risk loci in AFR children

| SNP | CHR | BP | EA | Closest gene | EAF (1KG) | Meta-analysis OR | Percentage of FRR explained |
| --- | --- | --- | --- | --- | --- | --- | --- |
| rs17133807 | 7 | 50409989 | A | *IKZF1* | 0.16 | 1.62 | 3.60% |
| rs7090445 | 10 | 61961417 | C | *ARID5B* | 0.19 | 1.72 | 5.15% |
| rs77632976 | 2 | 34828430 | T | *FAM98A* | 0.08 | 1.87 | 4.09% |
| rs112113758 | 3 | 2292232 | T | *CNTN4* | 0.09 | 2.09 | 6.67% |
| rs183221417 | 5 | 100102043 | C | *FAM174A* | 0.06 | 2.94 | 13.52% |
| rs867166159 | 9 | 191139 | T | *ZNG1A* | 0.05 | 2.69 | 9.39% |
| rs76135126 | 11 | 119033074 | C | *SLC37A4* | 0.08 | 2.02 | 5.45% |
| rs113299167 | 12 | 129961841 | T | *TMEM132D* | 0.07 | 1.99 | 4.67% |
| rs116677565 | 15 | 24174608 | C | *NDN* | 0.05 | 2.39 | 6.64% |
|  |  |  |  |  |  | **Total:** | 59.17% |

Abbreviations: rsid, genetic variant identifier using dbSNP build 151; CHR, chromosome; BP, base position, GRCh38 (hg38) build; EA(F), effect allele (frequency); OR, odds ratio.

**Supplementary Table 18**: B-ALL biological subtypes among COG clinical trial participants included in the ADMIRAL study

| **B-ALL subtype** | **n / N (%)** |
| --- | --- |
| Hypodiploidy |  |
| Negative | 426 / 440 (97%) |
| Positive | 14 / 440 (3%) |
| Missing | 14 |
| *BCR-ABL1* |  |
| Negative | 434 / 446 (97%) |
| Positive | 12 / 446 (3%) |
| Missing | 8 |
| *KMT2AR* |  |
| Negative | 409 / 423 (97%) |
| Positive | 14 / 423 (3%) |
| Missing | 31 |
| iAMP21 |  |
| Negative | 363 / 367 (99%) |
| Positive | 4 / 367 (1%) |
| Missing | 87 |
| Hyperdiploidy |  |
| Negative | 214 / 323 (66%) |
| Positive | 109 / 323 (34%) |
| Missing | 131 |
| *ETV6-RUNX1* |  |
| Negative | 320 / 428 (75%) |
| Positive | 108 / 428 (25%) |
| Missing | 26 |
| *TCF3-PBX1* |  |
| Negative | 228 / 285 (80%) |
| Positive | 57 / 285 (20%) |
| Missing | 169 |

**Supplementary Table 19**: Associations between African ancestry-specific B-ALL risk variants and B-ALL subtypes

|  |  | **Hyperdiploidy**  **(N=323, 109 positive)** | | | ***ETV6-RUNX1***  **(N=428, 108 positive)** | | | ***TCF3-PBX1***  **(N=285, 57 positive)** | | |
| --- | --- | --- | --- | --- | --- | --- | --- | --- | --- | --- |
| Risk variant | Alleles | Negative  n / N(%) | Positive  n / N(%) | P | Negative  n / N(%) | Positive  n / N(%) | P | Negative  n / N(%) | Positive  n / N(%) | P |
| rs77632976 | 0 risk alleles | 179/261 (69%) | 82/261 (31%) | 0.033 | 260/351 (74%) | 91/351 (26%) | 0.27 | 184/232 (79%) | 48/232 (21%) | 0.69 |
|  | ≥1 risk alleles | 26/50 (52%) | 24/50 (48%) |  | 52/64 (81%) | 12/64 (19%) |  | 38/46 (83%) | 8/46 (17%) |  |
|  | Missing | 9 | 3 |  | 8 | 5 |  | 6 | 1 |  |
| rs112113758 | 0 risk alleles | 171/256 (67%) | 85/256 (33%) | 0.27 | 252/344 (73%) | 92/344 (27%) | 0.020 | 180/225 (80%) | 45/225 (20%) | 0.85 |
|  | ≥1 risk alleles | 31/53 (58%) | 22/53 (42%) |  | 59/68 (87%) | 9/68 (13%) |  | 42/51 (82%) | 9/51 (18%) |  |
|  | Missing | 12 | 2 |  | 9 | 7 |  | 6 | 3 |  |
| rs28568357 | 0 risk alleles | 172/269 (64%) | 97/269 (36%) | 0.12 | 269/360 (75%) | 91/360 (25%) | 0.74 | 190/241 (79%) | 51/241 (21%) | 0.38 |
|  | ≥1 risk alleles | 33/43 (77%) | 10/43 (23%) |  | 43/55 (78%) | 12/55 (22%) |  | 32/37 (86%) | 5/37 (14%) |  |
|  | Missing | 9 | 2 |  | 8 | 5 |  | 6 | 1 |  |
| rs183221417 | 0 risk alleles | 185/275 (67%) | 90/275 (33%) | 0.31 | 278/364 (76%) | 86/364 (24%) | 0.10 | 199/247 (81%) | 48/247 (19%) | 0.80 |
|  | ≥1 risk alleles | 17/30 (57%) | 13/30 (43%) |  | 28/44 (64%) | 16/44 (36%) |  | 21/27 (78%) | 6/27 (22%) |  |
|  | Missing | 12 | 6 |  | 14 | 6 |  | 8 | 3 |  |
| rs115636216 | 0 risk alleles | 189/288 (66%) | 99/288 (34%) | 1.00 | 284/381 (75%) | 97/381 (25%) | 0.31 | 206/252 (82%) | 46/252 (18%) | 0.039 |
|  | ≥1 risk alleles | 16/24 (67%) | 8/24 (33%) |  | 29/35 (83%) | 6/35 (17%) |  | 17/27 (63%) | 10/27 (37%) |  |
|  | Missing | 9 | 2 |  | 7 | 5 |  | 5 | 1 |  |
| rs867166159 | 0 risk alleles | 184/280 (66%) | 96/280 (34%) | 0.64 | 285/379 (75%) | 94/379 (25%) | 0.65 | 199/252 (79%) | 53/252 (21%) | 0.38 |
|  | ≥1 risk alleles | 16/22 (73%) | 6/22 (27%) |  | 19/27 (70%) | 8/27 (30%) |  | 17/19 (89%) | 2/19 (11%) |  |
|  | Missing | 14 | 7 |  | 16 | 6 |  | 12 | 2 |  |
| rs112269413 | 0 risk alleles | 187/283 (66%) | 96/283 (34%) | 0.84 | 280/380 (74%) | 100/380 (26%) | 0.034 | 205/254 (81%) | 49/254 (19%) | 0.29 |
|  | ≥1 risk alleles | 18/28 (64%) | 10/28 (36%) |  | 30/33 (91%) | 3/33 (9%) |  | 17/24 (71%) | 7/24 (29%) |  |
|  | Missing | 9 | 3 |  | 10 | 5 |  | 6 | 1 |  |
| rs76135126 | 0 risk alleles | 180/269 (67%) | 89/269 (33%) | 0.27 | 271/357 (76%) | 86/357 (24%) | 0.60 | 189/236 (80%) | 47/236 (20%) | 0.67 |
|  | ≥1 risk alleles | 21/37 (57%) | 16/37 (43%) |  | 36/50 (72%) | 14/50 (28%) |  | 30/39 (77%) | 9/39 (23%) |  |
|  | Missing | 13 | 4 |  | 13 | 8 |  | 9 | 1 |  |
| rs113299167 | 0 risk alleles | 183/283 (65%) | 100/283 (35%) | 0.30 | 280/373 (75%) | 93/373 (25%) | 1.00 | 198/248 (80%) | 50/248 (20%) | 1.00 |
|  | ≥1 risk alleles | 22/29 (76%) | 7/29 (24%) |  | 33/43 (77%) | 10/43 (23%) |  | 25/31 (81%) | 6/31 (19%) |  |
|  | Missing | 9 | 2 |  | 7 | 5 |  | 5 | 1 |  |
| rs116677565 | 0 risk alleles | 192/285 (67%) | 93/285 (33%) | 0.085 | 281/377 (75%) | 96/377 (25%) | 0.31 | 201/254 (79%) | 53/254 (21%) | 0.43 |
|  | ≥1 risk alleles | 13/26 (50%) | 13/26 (50%) |  | 30/36 (83%) | 6/36 (17%) |  | 21/24 (88%) | 3/24 (13%) |  |
|  | Missing | 9 | 3 |  | 9 | 6 |  | 6 | 1 |  |
| Combined, all variants | 0 risk alleles | 64/98 (65%) | 34/98 (35%) | 1.00 | 90/127 (71%) | 37/127 (29%) | 0.18 | 67/76 (88%) | 9/76 (12%) | 0.036 |
|  | ≥1 risk alleles | 141/214 (66%) | 73/214 (34%) |  | 223/289 (77%) | 66/289 (23%) |  | 156/203 (77%) | 47/203 (23%) |  |

All p-values are from two-sided Fisher’s exact tests and not corrected for multiple testing.

**Supplementary Table 20**: Deaths among COG study participants in ADMIRAL discovery data in standard versus high risk treatment protocols

| Risk stratification | Study ID | Total N | Deaths  N (%) | Median follow-up (days) |
| --- | --- | --- | --- | --- |
| Standard | 9904 | 54 | 3 (21.4%) | 3920 |
|  | 9905 | 57 | 8 (57.1%) | 3438 |
|  | APEC14B1 | 54 | 3 (21.4%) | 1503 |
|  | Total | 165 | 14 (8.5%) | 2256 |
| High | 9906 | 16 | 4 (11.1%) | 2453 |
|  | AALL0232 | 164 | 29 (80.6%) | 2588 |
|  | AALL1131 | 10 | 1 (2.8%) | 1627 |
|  | APEC14B1 | 30 | 2 (5.6%) | 1183 |
|  | Total | 220 | 36 (16.4%) | 2187 |
| Overall | 9904 | 54 | 3 (5.6%) | 3920 |
|  | 9905 | 57 | 8 (14.8%) | 3438 |
|  | 9906 | 16 | 4 (7.4%) | 2453 |
|  | AALL0232 | 172 | 32 (59.3%) | 2565 |
|  | AALL1131 | 11 | 1 (1.9%) | 1626 |
|  | APEC14B1 | 87 | 6 (11.1%) | 1262 |
|  | Total | 397 | 54 (13.6%) | 2172 |

**Supplementary Table 21**: Five-year overall survival probability among carriers and non-carriers of novel African ancestry-specific B-ALL risk alleles by COG risk-stratified protocol groups

| Risk stratification | Risk alleles | Survival probability  (95% CI) |
| --- | --- | --- |
| Overall (N=397) | 0 alleles | 0.96 (0.92-1.00) |
|  | ≥1 alleles | 0.83 (0.79-0.88) |
| Standard (N=165) | 0 alleles | 0.97 (0.92-1.00) |
|  | ≥1 alleles | 0.91 (0.85-0.97) |
| High (N=220) | 0 alleles | 0.95 (0.90-1.00) |
|  | ≥1 alleles | 0.80 (0.73-0.87) |

**References**

1. Periasamy, P., Tran, V. & O’Neill, H.C. Identification of genes which regulate stroma-dependent in vitro hematopoiesis. *PLoS One* **13**, e0205583 (2018).

2. Mumme, H. *et al.* Single-cell analysis reveals altered tumor microenvironments of relapse-and remission-associated pediatric acute myeloid leukemia. *Nature Communications* **14**, 6209 (2023).

3. Kachuri, L. *et al.* Gene expression in African Americans, Puerto Ricans and Mexican Americans reveals ancestry-specific patterns of genetic architecture. *Nature genetics* **55**, 952-963 (2023).

4. Spaan, I., Raymakers, R.A., van de Stolpe, A. & Peperzak, V. Wnt signaling in multiple myeloma: a central player in disease with therapeutic potential. *Journal of hematology & oncology* **11**, 1-18 (2018).

5. Veiga-da-Cunha, M. *et al.* Failure to eliminate a phosphorylated glucose analog leads to neutropenia in patients with G6PT and G6PC3 deficiency. *Proceedings of the National Academy of Sciences* **116**, 1241-1250 (2019).

6. Bergerson, R.J. *et al.* An insertional mutagenesis screen identifies genes that cooperate with Mll-AF9 in a murine leukemogenesis model. *Blood, The Journal of the American Society of Hematology* **119**, 4512-4523 (2012).

7. Li, J. *et al.* The EMT transcription factor Zeb2 controls adult murine hematopoietic differentiation by regulating cytokine signaling. *Blood, The Journal of the American Society of Hematology* **129**, 460-472 (2017).

8. Ma, R. *et al.* Targeting pericentric non-consecutive motifs for heterochromatin initiation. *Nature* **631**, 678-685 (2024).

9. Vijayakrishnan, J. *et al.* Identification of four novel associations for B-cell acute lymphoblastic leukaemia risk. *Nature communications* **10**, 5348 (2019).

10. Jeon, S. *et al.* Genome-wide trans-ethnic meta-analysis identifies novel susceptibility loci for childhood acute lymphoblastic leukemia. *Leukemia* **36**, 865-868 (2022).

11. Papaemmanuil, E. *et al.* Loci on 7p12. 2, 10q21. 2 and 14q11. 2 are associated with risk of childhood acute lymphoblastic leukemia. *Nature genetics* **41**, 1006-1010 (2009).

12. Wiemels, J.L. *et al.* GWAS in childhood acute lymphoblastic leukemia reveals novel genetic associations at chromosomes 17q12 and 8q24. 21. *Nature communications* **9**, 286 (2018).

13. Vijayakrishnan, J. *et al.* The 9p21. 3 risk of childhood acute lymphoblastic leukaemia is explained by a rare high-impact variant in CDKN2A. *Scientific reports* **5**, 15065 (2015).

14. Perez-Andreu, V. *et al.* Inherited GATA3 variants are associated with Ph-like childhood acute lymphoblastic leukemia and risk of relapse. *Nature genetics* **45**, 1494-1498 (2013).

15. de Smith, A.J. *et al.* BMI1 enhancer polymorphism underlies chromosome 10p12. 31 association with childhood acute lymphoblastic leukemia. *International journal of cancer* **143**, 2647-2658 (2018).

16. Xu, H. *et al.* Novel susceptibility variants at 10p12. 31-12.2 for childhood acute lymphoblastic leukemia in ethnically diverse populations. *Journal of the National Cancer Institute* **105**, 733-742 (2013).

17. Treviño, L.R. *et al.* Germline genomic variants associated with childhood acute lymphoblastic leukemia. *Nature genetics* **41**, 1001-1005 (2009).

18. Vijayakrishnan, J. *et al.* A genome-wide association study identifies risk loci for childhood acute lymphoblastic leukemia at 10q26. 13 and 12q23. 1. *Leukemia* **31**, 573-579 (2017).

19. de Smith, A.J. *et al.* Heritable variation at the chromosome 21 gene ERG is associated with acute lymphoblastic leukemia risk in children with and without Down syndrome. *Leukemia* **33**, 2746-2751 (2019).

20. Mifsud, B. *et al.* Mapping long-range promoter contacts in human cells with high-resolution capture Hi-C. *Nature genetics* **47**, 598-606 (2015).

21. Javierre, B.M. *et al.* Lineage-specific genome architecture links enhancers and non-coding disease variants to target gene promoters. *Cell* **167**, 1369-1384. e19 (2016).

22. Barnett, K.R. *et al.* Epigenomic mapping reveals distinct B cell acute lymphoblastic leukemia chromatin architectures and regulators. *Cell Genomics* **3**(2023).

23. Bhattarai, K.R. *et al.* Investigation of inherited noncoding genetic variation impacting the pharmacogenomics of childhood acute lymphoblastic leukemia treatment. *Nature communications* **15**, 3681 (2024).

24. Mumbach, M.R. *et al.* HiChIP: efficient and sensitive analysis of protein-directed genome architecture. *Nature methods* **13**, 919-922 (2016).

25. Bergeron, B.P. *et al.* Epigenomic profiling of glucocorticoid responses identifies cis-regulatory disruptions impacting steroid resistance in childhood acute lymphoblastic leukemia. *Leukemia* **36**, 2374-2383 (2022).

26. ENCODE Project Consortium. An integrated encyclopedia of DNA elements in the human genome. *Nature* **489**, 57 (2012).

27. ENCODE Project Consortium. Expanded encyclopaedias of DNA elements in the human and mouse genomes. *Nature* **583**, 699-710 (2020).
