# Supplementary material for: Genome-wide association study of childhood B-cell acute lymphoblastic leukemia reveals novel African ancestry-specific susceptibility loci
Source: Nat Commun. 2025 Oct 22;16:8974. doi: 10.1038/s41467-025-64337-7 (PMC12546916; doi:10.1038/s41467-025-64337-7)
Supplement: Supplementary file 2 — Reporting Summary [file 41467_2025_64337_MOESM2_ESM.pdf]

nature portfolio

Corresponding author(s): Cindy Im, Logan G. Spector

Last updated by author(s): Aug 6, 2025

Reporting Summary

Nature Portfolio wishes to improve the reproducibility of the work that we publish. This form provides structure for consistency and transparency in reporting. For further information on Nature Portfolio policies, see our Editorial Policies and the Editorial Policy Checklist.

Statistics

For all statistical analyses, confirm that the following items are present in the figure legend, table legend, main text, or Methods section.

n/a	Confirmed	
☐	☒	The exact sample size (n) for each experimental group/condition, given as a discrete number and unit of measurement
☐	☒	A statement on whether measurements were taken from distinct samples or whether the same sample was measured repeatedly
☐	☒	The statistical test(s) used AND whether they are one- or two-sided<br>*Only common tests should be described solely by name; describe more complex techniques in the Methods section.*
☐	☒	A description of all covariates tested
☐	☒	A description of any assumptions or corrections, such as tests of normality and adjustment for multiple comparisons
☐	☒	A full description of the statistical parameters including central tendency (e.g. means) or other basic estimates (e.g. regression coefficient) AND variation (e.g. standard deviation) or associated estimates of uncertainty (e.g. confidence intervals)
☐	☒	For null hypothesis testing, the test statistic (e.g. F, t, r) with confidence intervals, effect sizes, degrees of freedom and P value noted<br>*Give P values as exact values whenever suitable.*
☒	☐	For Bayesian analysis, information on the choice of priors and Markov chain Monte Carlo settings
☒	☐	For hierarchical and complex designs, identification of the appropriate level for tests and full reporting of outcomes
☐	☒	Estimates of effect sizes (e.g. Cohen's d, Pearson's r), indicating how they were calculated

Our web collection on statistics for biologists contains articles on many of the points above.

Software and code

Policy information about availability of computer code

Data collection	No open source or custom code was used to collect these data.
Data analysis	Software and analytical tools used in data analyses include: PLINK versions 1.9 and 2.0 (https://www.cog-genomics.org/plink/) as specified in the Methods for quality control, association testing, LD-clumping, and PRS calculations; RFMix25 (v2.03-r0, https://github.com/slowkoni/rfmix) for global and local ancestry inference; NHLBI Trans-Omics for Precision Medicine or TOPMed62 Imputation Server (https://imputation.biodatacatalyst.nhlbi.nih.gov) for imputation; PCAMatchR67 (https://cran.r-project.org/web/packages/PCAmatchR/index.html) for control matching; METAL26 (https://github.com/statgen/METAL) and METASOFT70 (http://genetics.cs.ucla.edu/meta_jemdoc/) for meta-analysis; ANNOVAR73 (https://annovar.openbioinformatics.org/en/latest/) for functional annotation; coloc R package30 (v5.2.3, https://github.com/chr1swallace/coloc) for colocalization analysis; GCTA (v1.94.1, https://yanglab.westlake.edu.cn/software/gcta/#GREMLanalysis) for heritability analysis; and R v.4.2.1 for all other statistical analyses and plotting (https://www.R-project.org).

For manuscripts utilizing custom algorithms or software that are central to the research but not yet described in published literature, software must be made available to editors and reviewers. We strongly encourage code deposition in a community repository (e.g. GitHub). See the Nature Portfolio guidelines for submitting code & software for further information.

Data

Policy information about availability of data

All manuscripts must include a data availability statement. This statement should provide the following information, where applicable:

- Accession codes, unique identifiers, or web links for publicly available datasets
- A description of any restrictions on data availability
- For clinical datasets or third party data, please ensure that the statement adheres to our policy

Genotypes for cases and controls genotyped on the Global Diversity Array as part of this study are available for download at dbGaP (https://ncbi.nlm.nih.gov/gap/, data accession: phs004222.v1.p1). Access to individual-level data for ZOE 2.0 study participants57 are available through dbGaP (https://ncbi.nlm.nih.gov/gap/, data accession: phs002232.v1.p1). Access to Childhood Cancer Record Linkage Project data are not publicly available due to California Department of Public Health regulations; however, access may be granted by the Principal Investigators to bona fide researchers with the completion of data use agreements. Data from the Children's Oncology Group may be requested following organizational procedures (https://childrensoncologygroup.org/data-sharing). Whole blood eQTL data published by Kachuri et al. 29 were obtained from Zenodo (https://zenodo.org/, data accession: 7735723).

Research involving human participants, their data, or biological material

Policy information about studies with human participants or human data. See also policy information about sex, gender (identity/presentation), and sexual orientation and race, ethnicity and racism.

Reporting on sex and gender	Sex as a biological attribute was considered in this analysis. Information about socially constructed/relevant gender was unavailable. Cases were matched by sex to controls to support GWAS inference. Analyses stratified by sex to identify genetic variants with effects modified by sex were considered to be outside the scope of this analysis.
Reporting on race, ethnicity, or other socially relevant groupings	In this analysis, we distinguish between genetic ancestry and race and ethnicity as a social construct. For clarity, we chose to limit corresponding abbreviations and explicitly state which concept was relevant at each of the specified data collection and analysis stages throughout the manuscript (e.g., African ancestry versus African American race and ethnicity). All ancestry and race and ethnicity terms used in published work referenced by this manuscript were described in a manner that was consistent with corresponding publications to retain the accuracy of the original authors' descriptions of ancestry and race/ethnicity in their respective studies. Methods to infer genetic ancestry and related control for potential confounding are described in detail in the manuscript methods.
Population characteristics	Sex; age at B-cell acute lymphoblastic leukemia diagnosis; race and ethnicity; inferred genetic ancestry.
Recruitment	Participants with childhood acute lymphoblastic leukemia were recruited through the multi-institutional Children's Oncology Group (COG) frontline trials and at state cancer registry or academic institution biobanks. Potential selection bias due to sampling at academic versus private institutions is possible, but unlikely given the exposure of interest is germline genetics.
Ethics oversight	University of Minnesota Institutional Review Board (IRB) approval was received.

Note that full information on the approval of the study protocol must also be provided in the manuscript.

Field-specific reporting

Please select the one below that is the best fit for your research. If you are not sure, read the appropriate sections before making your selection.

[x] Life sciences [ ] Behavioural & social sciences [ ] Ecological, evolutionary & environmental sciences

For a reference copy of the document with all sections, see nature.com/documents/nr-reporting-summary-flat.pdf

Life sciences study design

All studies must disclose on these points even when the disclosure is negative.

Sample size	GWAS statistical power was not assessed because this is an observational study with a fixed sample size. However, our sample size is patently sufficient for the detection of larger per-allele odds ratios (ORs>2).
Data exclusions	This analysis was limited to individuals who reported Black or African American race and ethnicity. No other study data exclusions were applied.
Replication	As described in the manuscript, we identified a replication sample and present all replication results.
Randomization	This is an observational study (GWAS). Randomization is not applicable.
Blinding	See above. Blinding is not applicable to this analysis.

Reporting for specific materials, systems and methods

We require information from authors about some types of materials, experimental systems and methods used in many studies. Here, indicate whether each material, system or method listed is relevant to your study. If you are not sure if a list item applies to your research, read the appropriate section before selecting a response.

Materials & experimental systems

n/a	Involved in the study	
☒	☐	Antibodies
☐	☒	Eukaryotic cell lines
☒	☐	Palaeontology and archaeology
☒	☐	Animals and other organisms
☐	☒	Clinical data
☒	☐	Dual use research of concern
☒	☐	Plants

Methods

n/a	Involved in the study	
☒	☐	ChIP-seq
☒	☐	Flow cytometry
☒	☐	MRI-based neuroimaging

Eukaryotic cell lines

Policy information about cell lines and Sex and Gender in Research

Cell line source(s)	GM12878 B-lymphoblastoid cells (Coriell Institute for Medical Research, #NA12878) or 697 B-cell precursor acute lymphoblastic leukemia cells (DSMG; #ACC 42) were analyzed.
Authentication	STR profiling was utilized authenticate 697 and GM12878 cells.
Mycoplasma contamination	All cell lines tested were negative for mycoplasma contamination.
Commonly misidentified lines (See ICLAC register)	*Name any commonly misidentified cell lines used in the study and provide a rationale for their use.*

Clinical data

Policy information about clinical studies

All manuscripts should comply with the ICMJE guidelines for publication of clinical research and a completed CONSORT checklist must be included with all submissions.

Clinical trial registration	Data from COG protocols 9904, 9905, 9906, AALL0232, AALL1131, AALL15P1, AALL1621, and APEC14B1 were included.
Study protocol	This is an observational study, therefore a clinical trial protocol is not applicable.
Data collection	These methods have been described in the current manuscript. All COG protocols above have been described in other publications.
Outcomes	Primary outcome was acute lymphoblastic leukemia (WHO ICD-O-3 histology 9811-9818, 9820, 9823, 9826, 9827, 9831-9837, 9940, 9948) diagnosed at 0-25 years of age. A secondary outcome was overall survival (time at risk starting at diagnosis with censoring at last follow up).

Plants

Seed stocks	*Report on the source of all seed stocks or other plant material used. If applicable, state the seed stock centre and catalogue number. If plant specimens were collected from the field, describe the collection location, date and sampling procedures.*
Novel plant genotypes	*Describe the methods by which all novel plant genotypes were produced. This includes those generated by transgenic approaches, gene editing, chemical/radiation-based mutagenesis and hybridization. For transgenic lines, describe the transformation method, the number of independent lines analyzed and the generation upon which experiments were performed. For gene-edited lines, describe the editor used, the endogenous sequence targeted for editing, the targeting guide RNA sequence (if applicable) and how the editor was applied.*
Authentication	*Describe any authentication procedures for each seed stock used or novel genotype generated. Describe any experiments used to assess the effect of a mutation and, where applicable, how potential secondary effects (e.g. second site T-DNA insertions, mosiacism, off-target gene editing) were examined.*
