# Supplementary material for: Genome-wide association study of childhood B-cell acute lymphoblastic leukemia reveals novel African ancestry-specific susceptibility loci
Source: Nat Commun. 2025 Oct 22;16:8974. doi: 10.1038/s41467-025-64337-7 (PMC12546916; doi:10.1038/s41467-025-64337-7)
Supplement: Supplementary file 3 — Transparent Peer Review file [file 41467_2025_64337_MOESM3_ESM.pdf]

nature portfolio

Peer Review File

Genome-wide association study of childhood B-cell acute lymphoblastic leukemia reveals novel African ancestry-specific susceptibility loci

Corresponding Author: Dr Cindy Im

This manuscript has been previously reviewed at another journal. This document only contains information relating to versions considered at Nature Communications.

This file contains all reviewer reports in order by version, followed by all author rebuttals in order by version.

Version 1:

Reviewer comments:

Reviewer #3

(Remarks to the Author)
This is a study on risk loci associated with ALL in children of African genetic ancestry. The study addresses a knowledge gap considering that most previous GWAS studies have been done on individuals of European descent. The study is well carried out and the resulting data are of good quality, supporting the conclusions.

Open Access This Peer Review File is licensed under a Creative Commons Attribution 4.0 International License, which permits use, sharing, adaptation, distribution and reproduction in any medium or format, as long as you give appropriate credit to the original author(s) and the source, provide a link to the Creative Commons license, and indicate if changes were made.
In cases where reviewers are anonymous, credit should be given to 'Anonymous Referee' and the source.

The images or other third party material in this Peer Review File are included in the article's Creative Commons license, unless indicated otherwise in a credit line to the material. If material is not included in the article's Creative Commons license and your intended use is not permitted by statutory regulation or exceeds the permitted use, you will need to obtain permission directly from the copyright holder.
To view a copy of this license, visit https://creativecommons.org/licenses/by/4.0/

RESPONSE TO REVIEWERS

Re: NCOMMS-25-35400A

Manuscript title: “Genome-wide association study of childhood B-cell acute lymphoblastic leukemia reveals novel African ancestry-specific susceptibility loci”

Responses to Reviewer #3

(1) This is a study on risk loci associated with ALL in children of African genetic ancestry. The study addresses a knowledge gap considering that most previous GWAS studies have been done on individuals of European descent. The study is well carried out and the resulting data are of good quality, supporting the conclusions.

Response: We thank the reviewer for their feedback!
